# Pick Your Own Adventure: Orthogonal Methods for Analyzing Polyphenols

**DOI:** 10.1021/acs.jchemed.5c01459

**Published:** 2026-07-17

**Authors:** Amanda Rae Buchberger, Kristel M. Forlano, Madeline J. Lund, Pamela S. Doolittle, Dominic M. Colosi, Thao U. Duong, Brian J. Esselman, Kyoung-Shin Choi, Robert J. Hamers

**Affiliations:** † Department of Chemistry, 5228 University of Wisconsin-Madison, 1101 University Avenue, Madison, Wisconsin 53706, United States

**Keywords:** Second-Year Undergraduate, Analytical Chemistry, Laboratory Instruction, Inquiry-Based/Discovery Learning, Chromatography, Phenols, Quantitative Analysis, UV−vis Spectroscopy, Titration/Volumetric Analysis

## Abstract

The analytical chemistry classroom is naturally poised to demonstrate the use of orthogonal techniques to quantify different molecules such as polyphenols. Using food science as a lens and a molecule-centric, inquiry-based approach, this article describes a semester-long laboratory experience for students to adapt and use potentiometric titration, spectrophotometric, and chromatographic methods to analyze polyphenols (*i.e*., catechin and gallic acid) in instructor-provided samples. Students then are given the agency to select a sample of interest which (theoretically) contains the target analytes (or not) and justify the appropriate method for that sample. The implementation of this experience provides students with many opportunities to authentically engage in scientific practices, such as designing experiments, evaluating their results, and making evidence-based decisions about how to modify procedures and what samples to study. After completing this laboratory experience, students reported significant gains in learning laboratory techniques and moderate gains in designing experiments and analyzing data, while engaging in authentic analyses (including variability) in a supportive environment. Peer evaluations also indicated that students had positive experiences working in groups. Furthermore, the experience is incredibly flexible. Each standard method module (titration, spectrophotometry, chromatography) can be completed independently, or more orthogonal techniques (*e.g*., gas chromatography) could be added to expand the molecule-centric semester experience.

## Graphical abstract

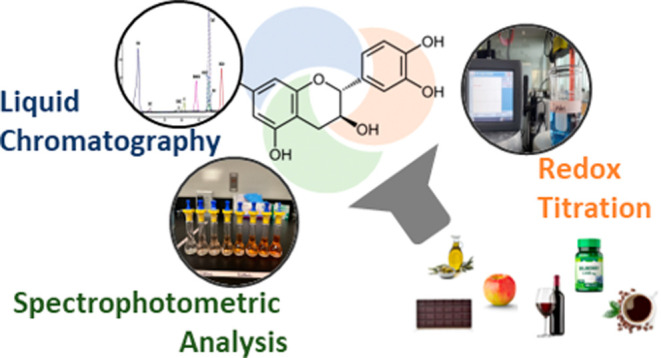

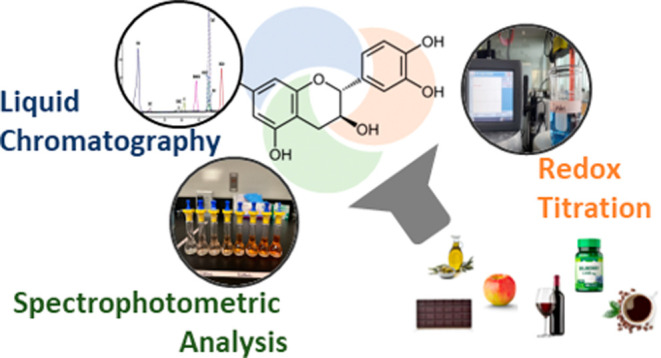

## Introduction

Analytical chemistry is well positioned to offer students an authentic opportunity to engage in the scientific practices^1–3^ while using techniques and instrumentation consistent with the day-to-day activities of many industrial and academic analytical chemists.^4,5^ Many traditional analytical chemistry courses use inauthentic laboratory exercises, where students focus on collecting data on instructor-prepared or instructor-chosen samples (rather than a sample of their choosing). The assessment goal is to obtain results that match the instructor’s expectation only (i.e., correct), and thus earn the best grade,^5,6^ rather than participate in or engage with scientific practices (no matter if the results are accurate or inaccurate).^7–11^ Additionally, traditional curricula often do not allow students to perform measurements on the same sample using multiple techniques to assess the “best” technique for the sample being analyzed. Unless a curriculum is explicitly designed with practical, authentic decision-making opportunities (either via choosing their own study/technique or investigating what an expert would study) or to address/experience experimental fault points, students will likely develop an inaccurate perception of what analytical chemists do.

Inquiry-based project laboratories or course-based undergraduate research experiences (CUREs) can provide opportunities for students to engage in authentic research experiences and reasoning.^5,11–19^ The recent popularity of these courses is also due to the high demand for traditional research experiences and the associated difficulty to obtain them due to a lack of funding, space, or opportunities for all interested students. While a standardized analytical chemistry curriculum can give breadth of content, it is desirable to give students more time to engage in the scientific practices^1^ (and increase the depth to their learning) using an inquiry-based framework for a project within a course.^7–9,20,21^ This can be done by embedding stand-alone projects or even replacing more standardized, confirmatory experiments with lower-level inquiry-based experiments, such as a μCUREs.^22^ One way to approach this is by selecting a molecule-of-interest that has accessible unknowns within the current literature. Furthermore, because no single experiment can cover all learning objectives or goals, it is important to diversify and invest in inquiry-based experiments over an entire course or program.^23^

Herein, a semester-long inquiry-based experience is described where two polyphenols, gallic acid and catechin, shown in Figure 1, are used to demonstrate the importance of orthogonal analyses, using the context of food science. Naturally found in many fruits, vegetables, and teas, polyphenols are a large, complex family of biological molecules that contain many phenol (C_6_H_5_–OH) groups.^24^ The same natural source can contain multiple polyphenols that are often present in different amounts in different locations within the source. For example, the pulp of fruits/vegetables is rich in nonflavonoids (single-ring polyphenols or polyphenol derivatives), while the skins, seeds, and stems are rich in flavonoids (two-ring polyphenols or polyphenol derivatives with structures similar to flavone).^24^ In biological systems, polyphenols behave as antioxidants.^24^ Uncontrolled oxidation inside living cells (e.g., of DNA, proteins) results in cellular damage, and antioxidants can react with these oxidizing species to prevent them from reacting with important biological molecules. Thus, consuming antioxidants has been associated with a variety of positive health effects.^24^ In this semester-long experience, teams of students gain experience in three orthogonal methods (meaning measuring the same quality (polyphenol concentration) by using different physical properties) to analyze the concentration of polyphenols in a variety of samples: (a) potentiometric/redox titration, (b) UV–vis spectrophotometry, and (c) liquid chromatography. Subsequently, students select a personal, accessible sample to analyze by one of those methods, justifying their choice of method with appropriate chemical reasoning. Within the literature, a student-selected sample and food stuff/beverage analysis are not unique.^23^ Instead, the teaching of key analytical techniques in this molecule-centric approach (meaning looking at methods to apply to a molecule of interest, rather than find a problem for a method of interest to analyze), along with justifying the best method for the sample of interest, is uncommon.^25,26^ Within this lab experience, students gain experience with analysis techniques and laboratory skills in high demand in industry, learn industry-prioritized skills,^4,5^ as well as engage with scientific practices.^8,9^

**Figure 1:**
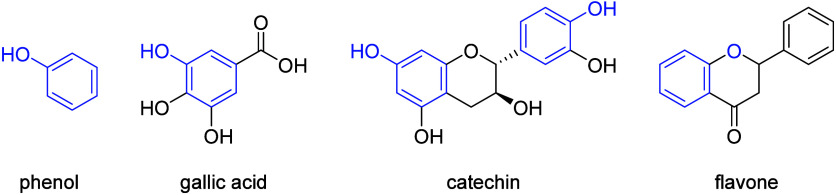
Phenol and two polyphenols, gallic acid (nonflavonoid) and catechin (flavonoid), with their phenol cores highlighted in blue. Flavone is provided as a complementary example.

## Laboratory Design & Implementation

### Classroom Context

This experience was implemented in the University of Wisconsin-Madison’s CHEM 329 (Fundamentals of Analytical Science, Honors) class. CHEM 329, which is designed for chemistry and chemical engineering majors, teaches the fundamentals of chemical measurement in chemistry, biology, engineering, geology, and the medical sciences. Topics include equilibria of complex systems, spectroscopy, electrochemistry, separations, and quantitative laboratory techniques. The learning objectives are provided in the Instructor Notes (in the Supporting Information), and this experience focuses on #5 (“Design and execute independent analytical chemistry experiments using methods sourced from the scientific literature.”). CHEM 329 has 4 h lab periods twice a week, with some proportion (usually 6–9 lab periods total) dedicated to an inquiry-based project lab in a normal semester. The other laboratory periods are dedicated to standard laboratories to give student fundamental skills in a technique they will perform in the inquiry-based lab experience (e.g., titrations, spectrophotometry, and chromatography) and expose students to different techniques (e.g., pH measurements, electrochemistry), explicitly assessing quantitative accuracy. Students also have two 50 min lecture periods and one 50 min discussion period each week, the latter being used to support either lab (experiment design, planning, and preparation) or lecture (practice problems, content review).

A preliminary version of this lab experience was run during the Fall 2023 semester (81 students), which included only 3 of the 4 lab modules. Fall 2023 and Spring 2024 had different instructors but used the same conditions and equipment. The remainder of this discussion will focus on the implementation in Spring 2024 (66 students), which included all 4 lab modules described below. The course had 4 sections of up to 18 students and 4 teaching assistants (TAs), with one TA assigned per section. The TAs were the primary supervisors of lab work and implemented planned activities during the discussion periods, while the instructors helped with lab observations, presented content during the lecture periods, and designed the curriculum (laboratory, lecture, and discussion). At the beginning of the semester, students formed groups of 3–4 and filled out group contracts (i.e., the Group Charter). Three lab periods were reserved per lab module (12 of 27 laboratory periods total, with two additional periods throughout the semester being used for preparation or assessment of the semester-long lab experience), which is uncommon compared to other lab activities.^23^ Students received a primer on necessary concepts for each lab module via a discussion worksheet focused on a relevant literature article, while other discussion periods gave time for students to work as a group on an assessment item (7 total of 14 over the semester; e.g., final presentation). Figure 2 shows a visual of the lab experience schedule. The full course schedule and activities/assessments (mapped onto both the scientific practices^1^ and industry prioritized skills^4^) can be found in the Instructor Notes (in the Supporting Information).

**Figure 2:**
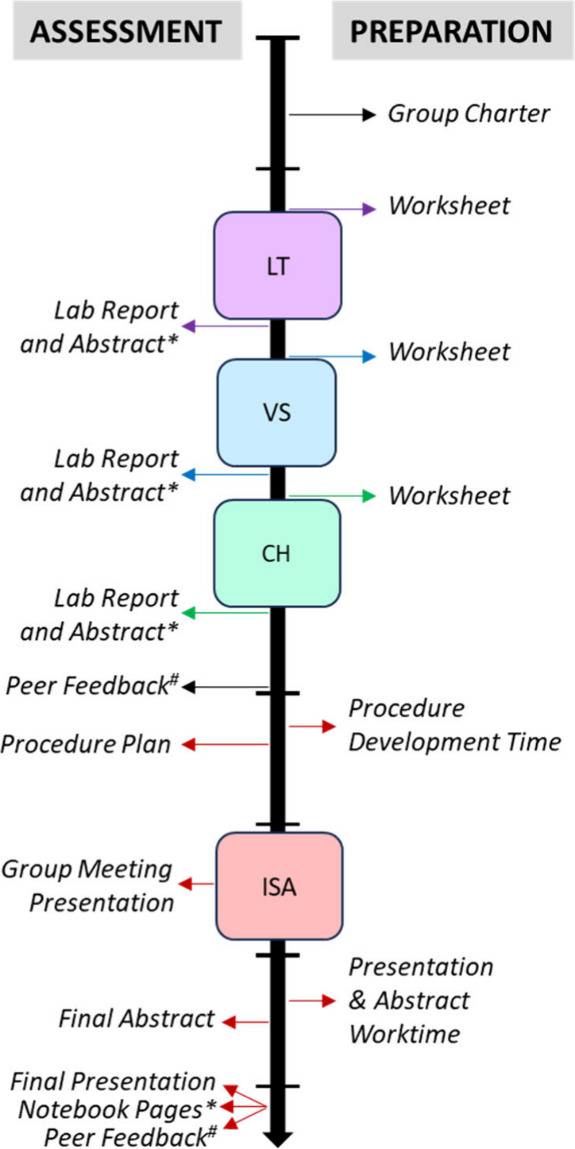
Timeline of the project lab experience (with each colored box representing a lab module), preparatory activities, and assessments. The black, horizontal lines denote 2-week intervals to demonstrate the timing of the experience across the semester. Assessments marked with an asterisk (*) were graded by the TA, while assessments marked with a pound sign (#) were graded by peers. Otherwise, a staff member/instructor graded the assessment. Of note, final presentations were graded by a mix of instructors and TAs. In total, the Spring 2024 semester was 14 weeks. The preparation and assessments are spaced to denote their general timing in the semester, and arrows (colored to match lab module; black if general) are used to connect to a particular lab module. LT = Lowenthal Titration. VS = Vanillin Spectrophotometry. CH = Chromatography. ISA = Independent Sample Analysis. See Instructor Notes (in the Supporting Information) for analysis of each activity and assessment for their intent to have students engage in the scientific practices^1^ and industry-prioritized skills.^4^

Student survey data were obtained to assess students’ perceptions of the skills gained during the experience (end-of-semester) and obtain demographic information (beginning of semester). The skill assessment survey questions (e.g., Likert, free-response, *etc*.) are included in the Instructor Notes (in the Supporting Information) and were adapted from the SURE survey^27^ with no additional validation. The survey respondents (62 of 66 students) were primarily comprised of first year students (78%) that were chemical engineering majors (58%). The other common majors were chemistry (24%) and biochemistry (13%). All surveyed students (*n* = 62) had taken or tested out of general chemistry (with a majority taking advanced, one-semester general chemistry (CHEM 109, 81%)), and some students (*n* = 15) had taken organic chemistry.^28,29^ Importantly, at the time of their CHEM 329 enrollment, only 14 students had participated in undergraduate research. A large number (44 students) had expressed a desire to do so in the future. The large number of students wishing to engage in research demonstrates the need for performing inquiry-based projects or CUREs. These data were collected for the purposes of program improvement, and thus IRB approval was not required. All student comments or choices were anonymized by an individual not involved with class before being returned to the main author.

### Classroom Design

The semester-long experience has the following learning outcomes for students:

1. Recognize and compare/contrast authentic modern analytical methods used to analyze polyphenols.
2. Construct and justify a method to analyze and statistically test a hypothesis focused on a sample of personal interest.
3. Analyze and validate results with appropriate statistical methods and quality assurance and control metrics, including detection limits, controls, and percent recovery of a spike.
4. Summarize and communicate findings in an oral presentation and written abstract.

Within this experience, the four lab modules follow the Laboratory Action-Based (LAB) theory^30^ as well as guidelines from Seery et al.^31^ The first three modules were developed based upon polyphenol reactivity: (1) Lowenthal titration (LT), (2) vanillin spectrophotometry (VS), and (3) chromatography (CH). Of note, spectrophotometry is a common methodology in inquiry-based lab experiments in analytical chemistry, but redox titrations and chromatography (specifically liquid chromatography with UV–vis and fluorescence detection) are more rare.^23^ These three modules were additionally designed/chosen due to ease of implementation with our class size and equipment availability, but they do not represent an exhaustive list of techniques that can be used for polyphenol analysis. The final module (Independent Sample Analysis (ISA)) has students select a method and sample of interest (usually a food, which is common in experiments in the literature^23^) to quantify polyphenols and test their hypothesis of interest. Each lab module has another set of general learning outcomes (with the lab-specific learning outcomes listed with each lab module below), including:

1. Adapt a previously published analytical method for 2 standard polyphenols (catechin and gallic acid) to our laboratory setting.
2. Create a coherent procedure by exploring undefined procedural steps and connecting appropriate conceptual knowledge.

Polyphenols of interest in all laboratories are gallic acid and catechin, identified for their (1) molecular simplicity compared to some other polyphenols,^24^ (2) low cost and availability, and (3) solubility relative to other polyphenols tested (e.g., quercetin and curcumin). The presentation in the students’ lab manual, written like a conversation between the lab staff and the students, is structured to model the method development process of adapting a literature procedure, including mathematical considerations, relevant quality assurance checks (i.e., detection limit, recovery of a spike, and controls), and practical implementation notes. Industrial and academic perspectives from experts on food analyses are also included. If relevant, students are provided with appendices at the end of the lab manual to provide deeper background or highlight nuances of these techniques. The entire experimental procedure for each analysis is designed to be completed in a single laboratory period by an experienced individual, but multiple class periods are provided for the student groups (2–4 people), similar to other inquiry-based experiments in the literature,^23^ to allow chances for the (natural) failures that occur when performing new and inquiry-based experiments.

Brief overviews of each experimental module will be discussed. All equipment, key experimental parameters, and example result(s) for each lab module are included in the (i.e., appropriate lab manual and Instructor Notes). Within the context of this lab experience, considering our learning outcomes (and the inquiry-based nature of the experiment and not knowing the “correct” answer), the assessments focused on correct interpretation rather than accurate results. Interestingly, this approach is common in the analytical chemistry lab experiment literature,^23^ where “inaccurate” (if known) or unexpected (if analytically valid, meaning no known issues in the method to cause inaccuracies) results are not utilized to deduct points as long as the issues and future steps are discussed.^32^ Finally, any data variations found in the below modules can (and should) be used by instructors as a conversation point to discuss the importance of careful lab skills and critical interpretation of the data with the students.

### Lowenthal Titration (LT)

The LT (also known as the Lowenthal permanganate titration) is a reduction–oxidation titration that is used in industrial cider analysis to determine the quantity of polyphenols (i.e., tannins^33^) in a sample.^34^ The LT module-specific learning outcomes for students are:

1. Evaluate a method’s reproducibility and repeatability.
2. Apply statistical methods to frame results and determine if sample populations are equivalent.

A visual abstract of the LT analysis is shown in Figure 3. Most of our students are familiar with simple titrations from previous chemistry courses, which include a single colorimetric end point and a well-defined mole ratio.^35^ But, in citations and references for the LT, both the end-point color and the mole-to-mole ratio are ambiguous.^34,36,37^ The expected mole ratio of polyphenol (PP)-to-permanganate is 2.5 (eq 1), based upon the redox reaction^38^ (and derived in the lab manual for students).

1 $$2.5PP\left(\mathrm{Reduced}\right)+8H^{+}+Mn{O_{4}}^{-}⇌2.5PP\left(Oxidized\right)+Mn^{2+}+4H_{2}O$$

**Figure 3:**
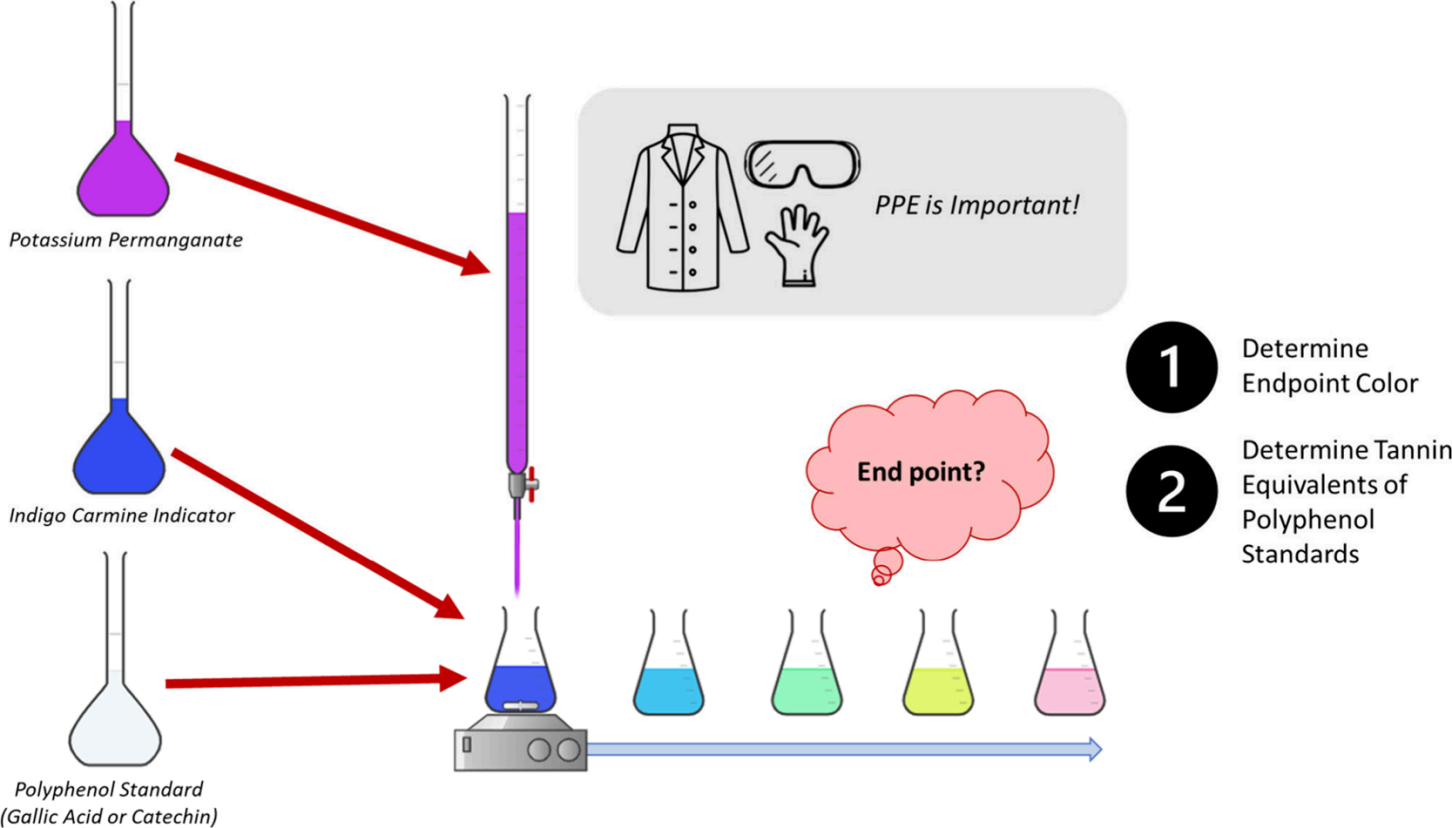
Visual abstract (provided to students in lab manual, see Supporting Information) describing the colorimetric portion of the Lowenthal Titration (LT) lab module. A standard solution of a polyphenol is titrated with potassium permanganate and indigo carmine to determine the equivalents of polyphenol (tannins) in the standard. Semester-long learning outcomes 1 and 2 are achieved by students through the LT lab module.

First, students complete an automated titration (using a Venier LabQuest equipped with an ORP probe and Drop Counter) to determine which color change (between blue, green, yellow, and orange/red; see Instructor Notes for video and more details) is associated with the titration’s equivalence point. Subsequently, students work in teams to compare the mole ratio that they individually determine for both polyphenols using a manual, colorimetric titration. Students apply statistical analyses to determine if the colorimetric titrations are repeatable (i.e., by one student using the same equipment in similar lab periods) and reproducible (i.e., by other students in their group using different pieces of equipment on the same day).^38^ Students also get experience with a blank titration (i.e., negative control, a titration with everything but the analyte of interest), the importance of pH control, and unit analysis when deriving equations that are provided with little context in the literature.

### Vanillin Spectrophotometry (VS)

Polyphenol content is often measured through spectrophotometry.^39,40^ While the Folin–Ciocalteu method (which uses a mixture of phosphomolybdic and phosphotungstic acid) is the most commonly used spectrophotometric method,^34^ vanillin is more accessible and cost-effective.^39^ In the acidic environment, vanillin is protonated, creating an activated electrophile and allowing nucleophilic attachment of the aromatic ring of the polyphenol to the electrophilic carbon atom of vanillin, through standard electrophilic aromatic substitution chemistry. The resulting solution of a *para*-quinone derivative has a red-orange color, amenable to measurement via spectrophotometry. The mechanism of this reaction is explored in a lab manual appendix to highlight the connection with the organic chemistry curriculum^28,29^ and the chemical reaction necessary for this analytical technique. The VS module has the following additional learning outcomes for students:

1. Construct calibration curves and determine a method’s detection limit under a variety of different conditions to probe the linear range and sensitivity of a method.
2. Assess the validity of the quantitative results for a provided, commercial sample via a spike recovery and positive control.

A visual abstract of the VS analysis performed by students is listed in Figure 4. Like the LT, the VS method has some authentic unknowns for students to address, including the solubility of vanillin in different solvents, the reactivity of vanillin with gallic acid and catechin in these different solvents, and the linear range of those that do react.^41,42^ Once they optimize the method, students apply their method to a commercial green tea tablet to quantify the polyphenols and perform multiple quality assurance techniques to validate the method (i.e., spike recovery to assess matrix effects on accuracy, method detection limit determination, and positive control analysis).

**Figure 4:**
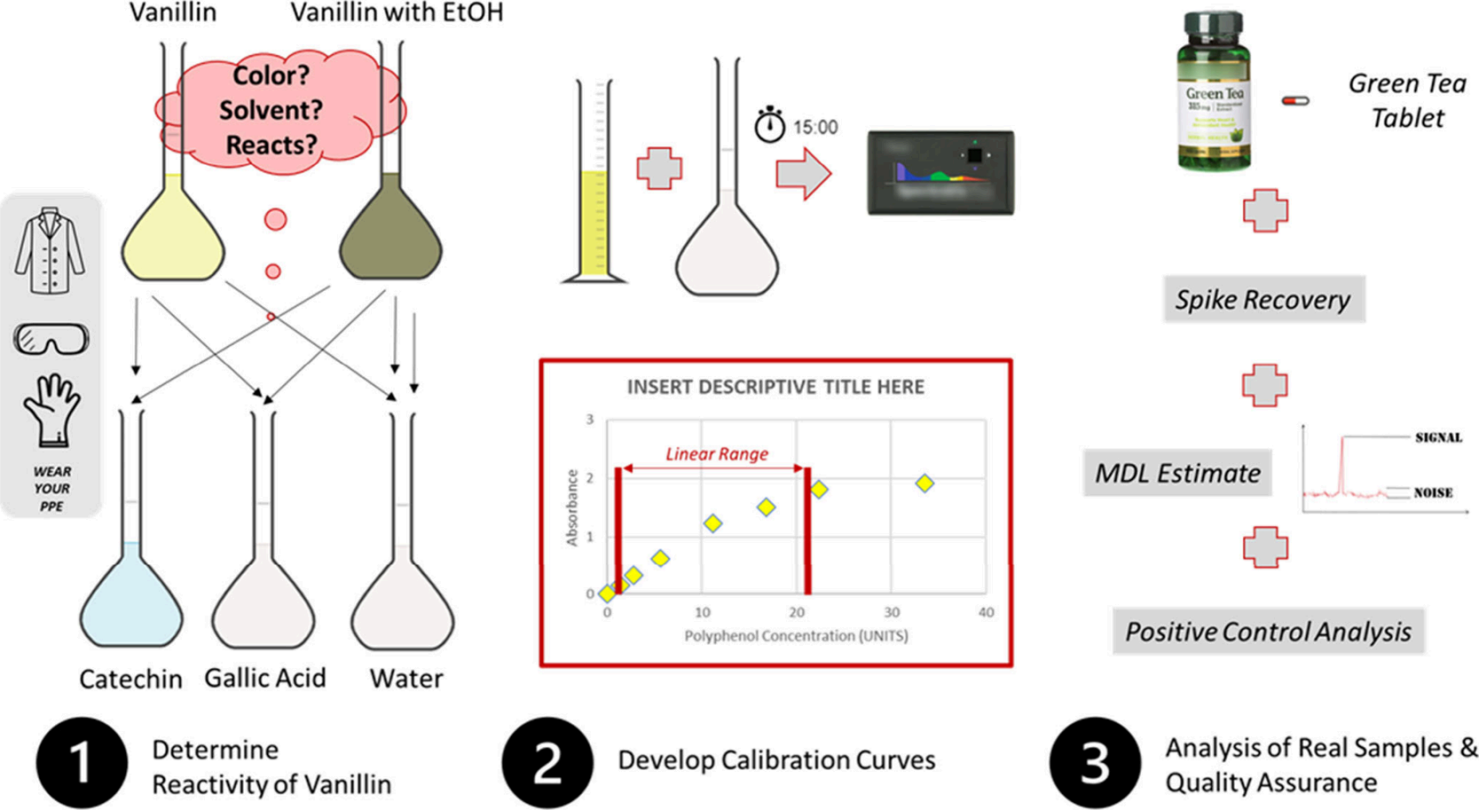
Visual abstract (provided to students in lab manuals, see Supporting Information) describing the Vanillin Spectrophotometry (VS) lab module. A standard solution of a polyphenol is reacted with vanillin to determine the concentration of polyphenols by UV–vis absorption of the resulting red-orange para-quinone. Semester-long learning outcomes 1, 2, and 3 are achieved by students through the VS lab module. “INSERT DESCRIPTIVE TITLE HERE” and “UNITS” are designed as a placeholder for students to fill in when they generate their own calibration curve(s).

### Chromatography (CH)

Modern day analysis of polyphenols, due to the diversity of their chemical and physical properties, is typically done via liquid chromatography (CH).^39,43^ The CH module has the following additional, lab-specific learning outcomes for students:

1. Assess a chromatography method’s results by evaluating replicates for consistency in retention time and quantitative accuracy using statistics.
2. Identify the capabilities and limitations of different instruments and instrument settings for the success of chromatographic separation.

A visual abstract of CH analysis is shown in Figure 5. In this lab module, students grapple with common method development decisions, such as (a) isocratic vs gradient elution, (b) manual vs automated injection, and (c) UV–vis vs fluorescence detection. Students analyze their polyphenol samples using both benchtop high-performance liquid chromatographs (HPLCs) and research-grade ultrahigh performance liquid chromatographs (uHPLCs). Students are given tours of both instruments, highlighting the major differences between them; students are also provided access to additional details presented in lab manual. Unless students have taken organic chemistry, they will be unfamiliar with how instruments display results, unlike spectrophotometry and titrations that they have been exposed to in general/high school chemistry. Thus, the CH lab module, unlike LT and VS, starts with a data analysis-focused laboratory period to teach students (a) how to analyze data from the HPLC and uHPLC instruments and (b) provide a “sample” gallic acid and catechin data set to compare their own results to. Then each student prepares a tea sample (black or green) to be analyzed by manual injection (with an isocratic elution by HPLC) and by automated injection (with a gradient elution by uHPLC). After the data were collected and shared with all students in the class, students analyze their data and compare the agreement (via statistics) of the quantity of each polyphenol (i.e., catechin and gallic acid) between each instrument method and across the class.

**Figure 5:**
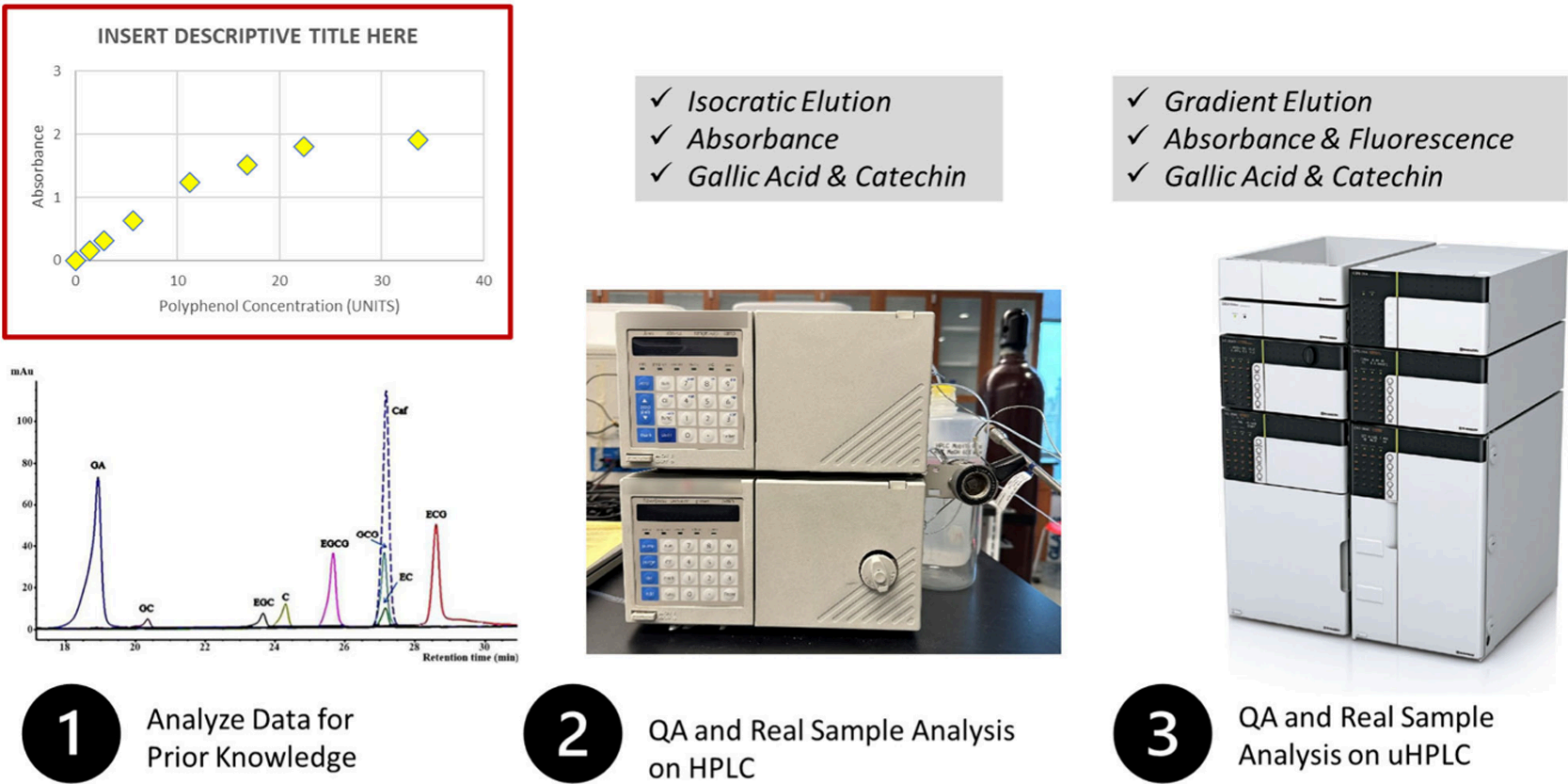
Visual abstract (provided to students in the lab manual, see Supporting Information) describing the Chromatography (CH) lab module. Standard solutions of polyphenols are injected separately between instruments, and the results are compared. Semester-long learning outcomes 1, 2, and 3 are achieved by students through the CH lab module. “INSERT DESCRIPTIVE TITLE HERE” and “UNITS” are designed as a placeholder for students to fill in when they generate their own calibration curve(s). QA = Quality Assurance. HPLC = High Performance Liquid Chromatograph. uHPLC = Ultra High Performance Liquid Chromatograph. The chromatogram was reprinted with permission.^43^

### Independent Sample Analysis (ISA)

After completion of the initial 3 lab modules, students select a sample of interest and develop a statistically testable hypothesis that uses the analytical methods developed. Since this is the cumulative experience of the semester, the learning objectives are the same as the semester-long experience stated above, especially 2–4, linking together all the skills they have previously learned.

As stated in the student laboratory manual, *‘Your goal should be more than just to “see if the packaging is correct”.’* Students select the LT or VS for primary analysis but have the option to use CH as an orthogonal method to confirm their results or obtain complementary information to their primary analysis. This curricular requirement is to standardize the amount of student work required to perform the analyses (as sample preparation for CH is generally simpler). Students justify their method choice (between the LT and VS) based upon the physical or chemical nature of their sample. According to the lab manual, *‘A justification should be more than just “I like this method better”. It should be quantifiable and convince an audience that your method is the obvious choice.’* Furthermore, students perform a minimum of two quality assurance metrics to assess the quality of their results, which is especially important if they make any modifications to their original methodology to make it compatible with their sample. Examples include controls (positive or negative), method detection limits, spike recoveries, *etc*.

### Hazards and Limitations

The LT and VS laboratory modules both utilize concentrated sulfuric acid. All excess should be neutralized prior to being poured into the sink. The LT module uses potassium permanganate, which is a strong oxidizer. Reacted potassium permanganate can be put down the sink, but unreacted potassium permanganate should be either (a) recycled (by collecting the excess, undiluted potassium permanganate for future students) or (b) chemically reduced (e.g., with ferrous ammonium sulfate) before being put down the sink. Proper protective equipment (goggles, gloves, and lab coat) is required.

The modular nature of this experience (LT, VS, and CH) allows for easy instructor adaptation to a variety of learning environments (see Instructor Notes in the Supporting Information for more information about the limitations and nuances of each method). If HPLCs are not available or are not available with the options described in this work (i.e., automated injection, fluorescence detection), the LT and VS modules can be completed on their own.

### Student Experimental Results Overview

Within the LT literature, the end point is listed as green, yellow, or even yellow with a red-tinge.^34,36,37^ Our students generally agree that the correct end point color is green-yellow (Figure 6A). The mole ratio, though, determined by the students was surprisingly variable (Catechin-to-KMnO_4_ ratio: 0.62 ± 0.66; Gallic Acid-to-KMnO_4_ Ratio: 0.56 ± 0.47); this variation was also present in the Fall 2023 implementation of this lab (see Table S2, which includes a 95% confidence interval to confirm the statistical difference between the expected and determined ratios). A student example is shown in Table S3, highlighting both the lack of repeatability and reproducibility. The variation could be due to incorrect calculations (such as not subtracting the volume of the blank (see Figure S1B)), poor titration technique, or improper detection of the end point color, the latter being the most likely cause, which is not surprising.^44^ The LT has been found to be repeatable (0.70% RSD) within a single day by a single person using gallic acid.^34^ One key difference is that we did not standardize our potassium permanganate (which tends to be contaminated with MnO_2_ or degrade easily), which would cause a depression in the expected ratio (unknowingly titrating a greater volume of KMnO_4_ with a lower concentration to reach the colorimetric end point). Other, optional variations of this lab should include either time for staff or students to standardize the potassium permanganate by titration with oxalic acid to determine if a more accurately known potassium permanganate concentration reduces the variability,^34^ but this has not been incorporated into the included version of this lab manual.

**Figure 6:**
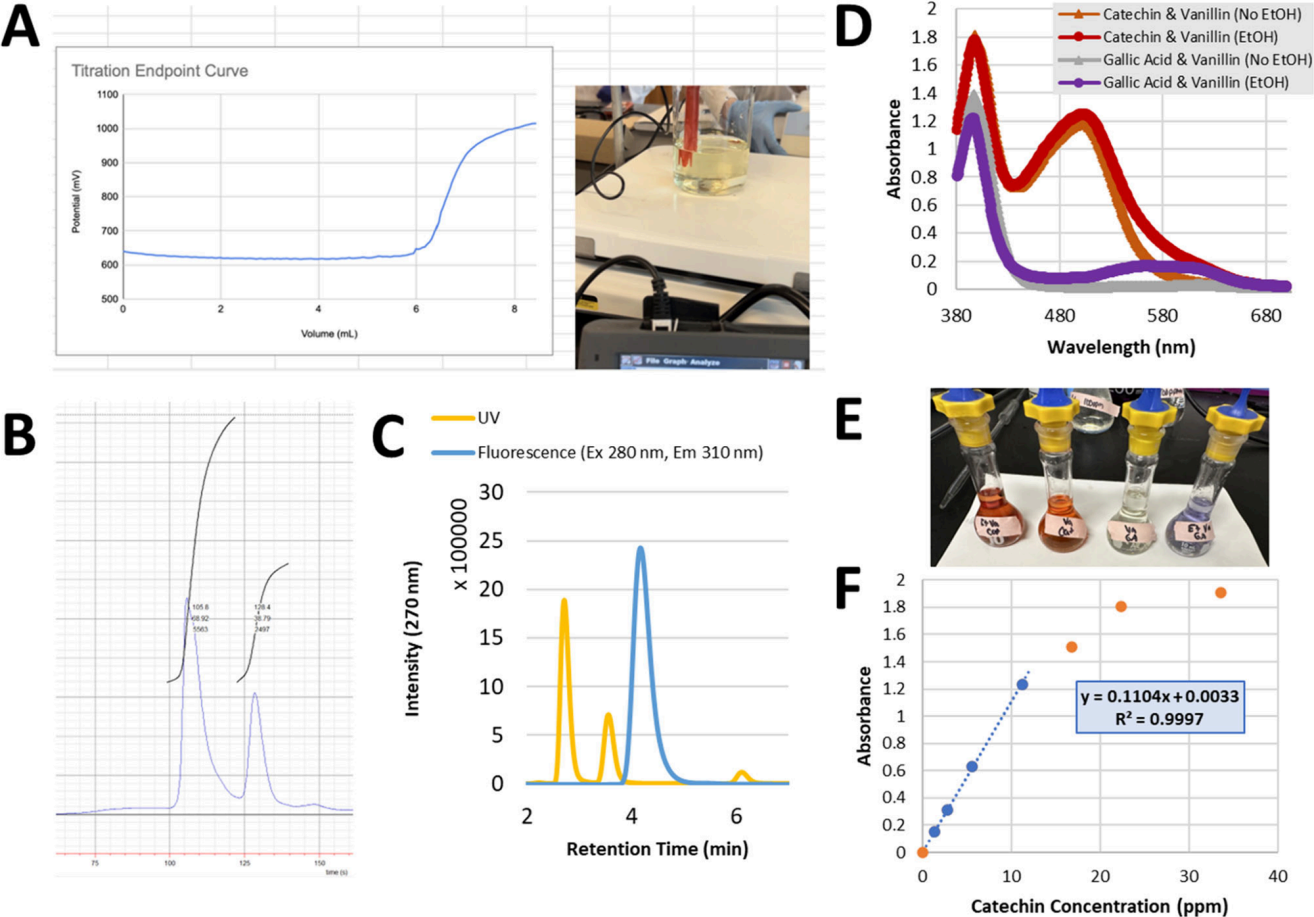
Example results found for the LT (A), CH (B–C), and VS (D–F) lab modules. (A) Screenshots of a student’s lab reports, depicting titration curve obtained by automated titration (and the color at the equivalence point). (B) Representative HPLC data of a green tea sample; the catechin and gallic acid eluted around the same time as the first peak at ∼105 s. (C) Representative uHPLC data for a student group’s positive control (a mix of catechin (∼3.5 min) and gallic acid (∼2.5 min)) are shown with both UV–vis and fluorescence detection. Gallic acid does not fluoresce; thus, the only peak present is catechin (∼4 min). The delay in detection is due to the flow path of the analyte through the instrument. The UV data was scaled 10× to match the peak size for fluorescence. (D) Full wavelength spectra of each polyphenol with the 2 different vanillin mixtures (with and without ethanol). The instrument was blanked with DI water for this experiment. The peak at ∼390 nm represents vanillin. The peak at ∼500 nm is the polyphenol complex with vanillin. A representative vanillin-only spectra can be seen in the Instructor Notes (Figure S5). (E) An image of the vanillin solutions (in water or ethanol/water) for both gallic acid (right two) and catechin (left two). Based upon a red color of the *para*-quinone expected when polyphenols react with vanillin, it does not appear that gallic acid has reacted, which is confirmed by spectrophotometry measurements (D). (F) An instructor-collected calibration curve for catechin, demonstrating the dynamic (orange) and linear range (blue).

For the VS experiments, all students eventually concluded that gallic acid did not react sufficiently with vanillin to be detected (Figure 6D and E), which is consistent with previous reports.^42^ Vanillin (in 70% sulfuric acid) has an absorbance peak at ∼390 nm (Figure S5), which some students initially interpreted as the gallic acid (reacting with vanillin) peak (Figure 6D). In a variation of this lab, students could analyze (a) pure vanillin and (b) a higher concentration of gallic acid mixed with vanillin to help avoid this misassignment (which has been incorporated into the version of the lab manual in the Supporting Information). When students did plot the linear response of this vanillin peak, they realized quickly that it could not have been gallic acid reacting with vanillin (Figure S3). The solvent choice (water or ethanol/water) for the vanillin solution did not impact the reaction or analysis (Figure S4). Students were equally split between which solvent they employed for their final analysis. Students were easily able to discriminate the linear range from the dynamic range of the calibration curve (Figure 6F; Figure S6A and S6B), which was similar each day of the experiment (Figure S6C and S6D). Students determined that the method detection limit of this assay was low but also variable depending upon the solvent choice (0.16 mg/L in ethanol/water and 0.59 mg/L in water, respectively). Students were given a lot of freedom on how to prepare their commercial green tea tablet for analysis, leading to a wide range (50% up to 400%) of spike recoveries. During testing, we observed that less concentrated solutions would have a more ideal spike recovery (i.e., 100% of the concentration of the spike is measured in the sample) with the green tea table samples. Students were able to analyze the green tea tablet, showing that the 15% polyphenols (as stated on the packaging) were not just catechin and catechin-like species (median 32.76 mg rather than 42.25 mg on packaging).^45^

For the CH experiments, when the isocratic separation (on the HPLC) is performed, gallic acid and catechin separate but only minimally (Figure 6B), making quantitation of each individual species not possible with peak area or peak height, especially within the tea sample. With the current gradient uHPLC method, catechin and gallic acid were separated with an appropriate resolution for quantitation (Figure 6C). Interestingly, gallic acid does not fluoresce, while catechin does (Figure 6C). It is unclear if other molecules are coeluting with these 2 polyphenols in the green tea sample, but evidence supports that this is the case because the catechin concentration was found to be substantially different between absorbance and fluorescence (244.77 mg vs 31.77 mg, respectively, via peak height of brewed green tea during instructor testing). To explore this further, samples of theophylline, theobromine, and caffeine, expected components of green tea, were all analyzed by uHPLC with the current HPLC and uHPLC methods. Theobromine and theophylline both eluted at similar retention times to catechin; thus, samples that were rich in these molecules (e.g., tea, coffee) could influence HPLC and uHPLC results (Figure S8). Future iterations of this experiment will try to resolve these common molecules more effectively before running student samples.^47^

Overall, the ISA experiment was successful for many students (Figure 7), providing quantitative, statistically testable results. Comparisons grouped generally as:

- Organic vs Nonorganic
- Comparing different flavors or brands (Figure 7B)
- Off- vs onbrand
- Alcoholic vs nonalcoholic

**Figure 7:**
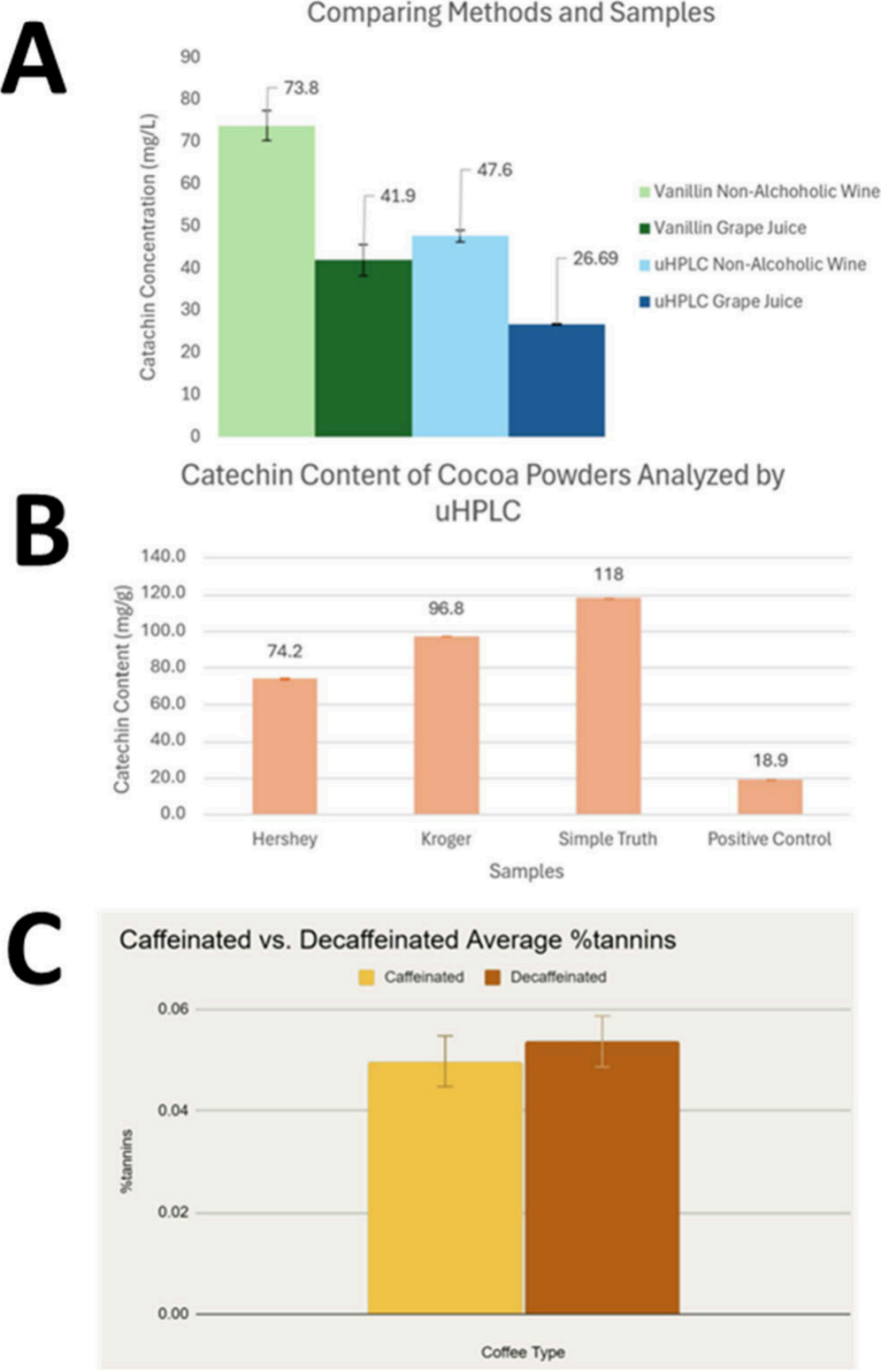
Sample data obtained by student groups during the ISA module of the lab experience. (A) Comparison of VS and CH analysis of grape juice versus nonalcoholic wine. The error bars represent the standard deviation. (B) uHPLC analysis of different cocoa powders. The first three bars are different brands of cocoa powder, while the last bar represents a student-created positive control (unknown true concentration). (C) LT quantitative results for percent tannins in caffeinated vs decaffeinated coffee samples. The error bars represent the standard deviation.

Common sample choices in Spring 2024 included milk, wine, chocolate, and supplements. Unique samples included black walnut sap, olive oil, and witch hazel (Figure S11). A full list of the comparisons/samples is given in the Instructor Notes (in the Supporting Information). When selecting appropriate quality assurance metrics, most students decided to use a positive control (just catechin or gallic acid in water), with a few students selecting to perform a negative control (and replicating the matrix of their samples, e.g., adding sugar). Some samples came with their own challenges, including mangos (where the LT indigo carmine indicator interacted with the pulp of the mango (Figure S9)) and apple juice (which can have ascorbic acid added as an antioxidant preservative, thus causing a systematic error in the amount of polyphenols measured by LT). Both situations were easily remedied by filtering pretitration or using an ascorbic acid control, respectively.

While the Spring 2024 session students preferred using the VS method as their primary analysis method, the Fall 2023 semester had students primarily using the LT method; this difference is likely due to both personal preference and the different samples selected. When selecting their primary analysis method, students should consider that samples will have different types of expected polyphenols. If the types of polyphenols are unknown, the LT is more generic compared to VS, which is more specific to catechin and equivalent species, such as anthocyanins.^41,42^ Thus, if the sample is rich in gallic acid or other nonflavonoids, performing a VS analysis would be ill-advised (unless the hypothesis is to confirm that assumption). Both the VS and LT methods are compatible with liquid or solid samples (if they can be dissolved in water) but also depend upon color. Some students naturally selected red samples and avoided VS. Interestingly, VS is used commonly for quantitation of anthocyanins, the polyphenol pigment that colors cranberries and other red fruits and vegetables.^41,42^ In final assessments, students also referenced the quality assurance metrics explored, including repeatability, reproducibility, and lower error toward the MDL (considering the optimal range of their buret is 25–40 mL) to help them select their primary analysis method. While not routed in scientific evidence, one group selected the VS method to avoid bias, as one of their group members was colorblind and could not reliably determine the colorimetric end point of the LT.

Only a few groups performed a truly orthogonal study of their selected sample by also analyzing their samples via CH. VS and CH data are expected to be more comparable (theoretically both directly measure catechin specifically), meaning that combination could be used to confirm quantitative accuracy (e.g., in olive oil). In some cases, though, such as the comparison between nonalcoholic red wine and pure red grape juice, the students thought that the CH data under-reported the catechin content (Figure 7A), but, more likely, this data set shows that something else besides catechin was also reacting with vanillin to inflate the concentration determined from VS. Another group analyzing cocoa powder (Figure 7B) saw the opposite trend (VS results shown in Figure S10). In another example (witch hazel), the LT was used for quantitation of overall polyphenol content, but CH was used to identify if catechin or gallic acid contributed to that content (which they did not) (Figure S11). Another example (decaffeinated vs caffeinated coffee, Figure 7C) found that, while there was no difference in polyphenol content found by LT, CH data showed that there was a significant difference between the quantity of catechin but not gallic acid for these two sample types.

### Student and TA Experience

Survey results showed that students perceived that they learned key skills, such as laboratory technique, analyzing and interpreting results, and designing experiments (Figure 8), all of which are skills industry partners wish future analytical chemists would have^4^ and connect to desirable scientific practices.^1^ Notably, using related-samples Friedman’s two-way analysis of variance by ranks, the perceived gains were not the same (*p* < 0.001, DOF = 8), with “Learning laboratory techniques” being statistically different than all others perceived gains in skills (*p* < 0.05, Bonferroni corrected for multiple tests). These perceived gains could be reflective of what they spent the most time on during the experience. Importantly, students do recognize that analytical chemistry is important (and practical), with one student stating (from the end-of-semester survey) “I think analytical chemistry is more like a tool to me ... I’m glad [to] learn a lot of analytical skills though”, while another student shared “A Chem is ... practical ... I agree that A Chem is necessary”.

**Figure 8:**
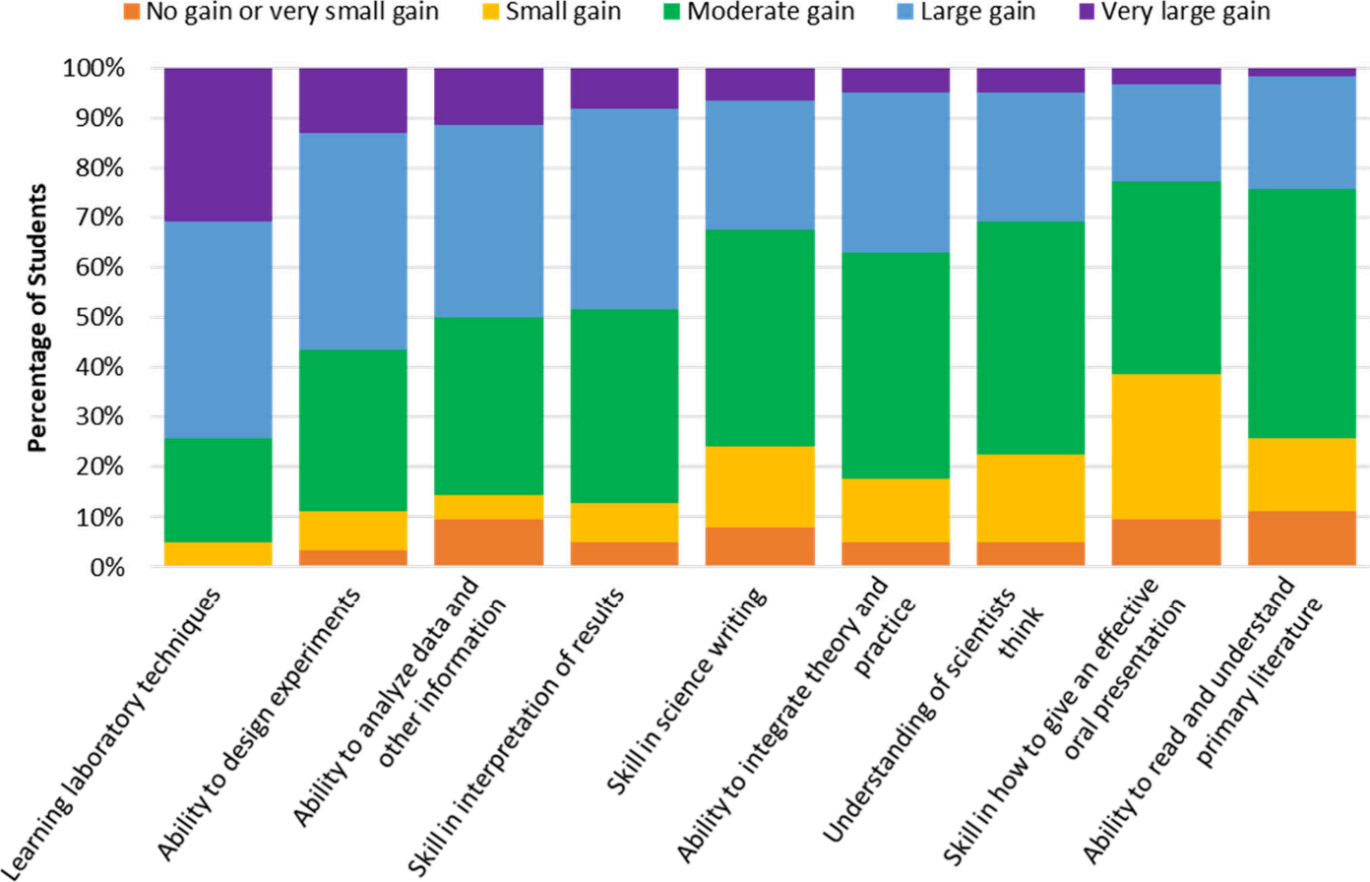
Perceived skill gains by students (*n* = 62 of 66 students) from the end-of-semester postsurvey. “Learning laboratory skills” had the largest observed gain (with the largest purple section), but all skills assessed had over 50% of the survey respondents having at least a “moderate gain”. “Learning laboratory techniques” was found to be statistically different (*p* < 0.05) from all other perceived skill gains according to a related-samples Friedman’s two-way analysis of variance by ranks test followed by a Bonferroni-corrected pairwise comparison.

Overall, working in the same groups for the entire semester/each lab module created a good working community. Quantitatively, students gave each other an average of 19.43 (±0.88) out of 20 points for the final peer feedback grades, which allowed peers to assess students’ communication abilities, preparedness, and respect for other group members. These grades could be inflated due to students not wanting to impact others’ grades, but the average grade still reflects a positive experience working in groups.

From in-lab observations and conversations, students struggled with adjusting their methods when they encountered difficulties. For some students, they switched to a different sample. As previously mentioned, one group struggled with the mango pulp (Figure S9); they were considering switching their sample to something “easier” before an instructor worked with them to think of solutions and reminded them that the assessments focused on interpretation, rather than a definitive answer. Generally, due to this challenge and being without explicit step-by-step instructions, no student group completed the experiments the exact same way or in the same time frame. All students completed the experiments within the 3-day periods provided, but some, as expected, completed the experiments in a single day. Due to this different lab structure, many students (and instructors/TAs) expressed discomfort with the unknown. In particular, the TAs would vary in their implementation of guidance, some giving direct instructions while others leaving students to struggle (i.e., no guidance). Discomfort, especially when doing something ‘new’, is not unusual and more common with students and TAs influenced by the pandemic.^46^ Depending upon teaching experience, TAs may have different beliefs about their role in an inquiry-based lab. In previous studies,^47^ it took an entire semester for some TAs to perceive themselves as ‘facilitators’ rather than ‘disseminators’ of content. To help with this discomfort, having the TAs complete a (truncated) form of the lab could be helpful, if time allows.

Our two major assessments, a final written abstract and oral presentation (to provide variety of assessments^31^), were successfully implemented (with grades of 11 (±1)/15 and 43.5 (±4.2)/50 points, respectively). Both rubrics are provided in the Supporting Information (see Instructor Notes). Students had multiple opportunities to practice both assessment types (through the initial three experiment modules’ lab reports for the abstract; during informal practice presentations and short, assessed group meeting presentations for the oral presentation; see Figure 2 for timetable) and were provided feedback by staff and/or TAs. Of note, the generation of these assessments (as well as planning assignments), inside and outside the classroom, allowed for peer-to-peer engagement in the scientific practices (see Table S10). In the final abstracts, students struggled with providing the appropriate details (i.e., when to be quantitative, when to provide statistical results, when to remove outliers) as well as connecting their thoughts. Of note, students consistently articulated a good understanding of the broad importance of polyphenol analysis in both assessments, but some students did not provide compelling reasons for justifying their chosen analysis method or why we should care about the specific hypothesis being tested. In the final presentation, students commonly forgot to discuss the chemistry of what they were studying (i.e., “what was the importance of vanillin in the VS experiment?”; “what is Beer’s Law, and how does it relates to your calibration curve?”; “how does KMnO_4_ react with the polyphenols?”; “what other molecules are present in your sample to compete/co-elute”, etc.), which also reflected in a poor justification of the method. These comments were also consistently found in the grading of the final abstract; it is worth noting that the feedback from the final abstract (given by the instructor of record) was returned before the final presentations to prompt students to address these items. In the next iteration of this lab, explicit class time (such as discussion, lecture, or lab) will be used to break down an abstract and how to write an effective one rather than just providing a handout (see How to Tell a Story Student Reference in Supporting Information).

## Conclusion and Future Variations

Moving forward, optional variations of the lab experience should be considered sufficiently modular; any of the individual lab modules can be used independently or in different combinations. An area of interest is to expand this laboratory experience to include a module with gas chromatography–mass spectrometry (GC-MS) on derivatized polyphenols to expand to more relevant, orthogonal techniques.^4,48^

Overall, this inquiry-based, semester-long experience was successful at giving students exposure to working with unknowns (samples and features of methods) and orthogonal methods while giving the opportunity to gain key skills for research and future careers as a scientist. Individually, each lab module was successfully implemented in the classroom, demonstrating diversity of analytical techniques that can be used to answer a problem, but they have the depth to be independently integrated into a standard lab or method-focused inquiry-based project as well.^4^ The time involved, however, to complete this full experience for the students (or even the individual modules) is more substantial than traditional experiments.^9,23,49^ We have chosen to have students engage in fewer experiments more deeply than more experiments more superficially. The balance of confirmatory and inquiry-based experiences is important and should be carefully considered when implementing any curriculum.

## Supplementary Material





















## Notes

How to tell a story student reference: document for students that gives instruction on how to put together a scientific abstract and presentation (PDF)
Instructor notes: supplemental notes on each experiments (LT, VS, CH, ISA) and other relevant information, such as supplemental figures and tables, course learning objectives, each activities/assessments intention, grading rubrics, and end-of-semester survey questions (PDF)
Lab manual for chromatographic separation of polyphenols using HPLC (CH) Lab Module: Lab manual for the CH experiment that includes background, prelab exercises, in-lab procedure, and postlab assessments (PDF, DOCX)
Lab manual for independent student analysis (ISA) lab module: lab manual for ISA experiment that includes background, prelab exercises, in-lab procedure, and postlab assessments (PDF, DOCX)
Lab Manual for Spectrophotometric Analysis of Polyphenols using Vanillin (VS) Lab Module: Lab manual for VS experiment that includes background, prelab exercises, in-lab procedure, and postlab assessments (PDF, DOCX)
Lab Manual for Titration Analysis of Polyphenols using Lowenthal Titration (LT) Lab Module: Lab manual for LT experiment that includes background, prelab exercises, in-lab procedure, and postlab assessments (PDF, DOCX)
