# Supplementary material for: Pick Your Own Adventure: Orthogonal Methods for Analyzing Polyphenols
Source: J Chem Educ. 2026 Jul 17;103(8):4243–54. doi: 10.1021/acs.jchemed.5c01459 (PMC13480120; doi:10.1021/acs.jchemed.5c01459)
Supplement: Supplementary file 1 [file ed5c01459_si_001.pdf]

Last Updated Spring 2024

How to Tell Your Story

(when writing abstracts or giving presentations)

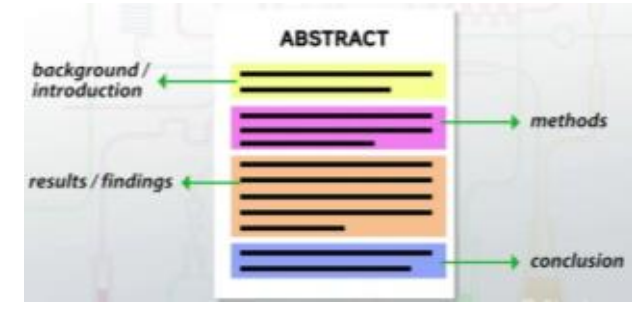

Abstract: A concise (200-300 word) written summary (usually a paragraph) of a study/story and its key message/purpose. This writing provides the first impression of your study.

Presentation: A visual and oral display (*i.e.*, poster or PowerPoint) of your study/story, with the length being variable (*e.g.*, 5-60 minutes) depending upon the venue and the desired audience.

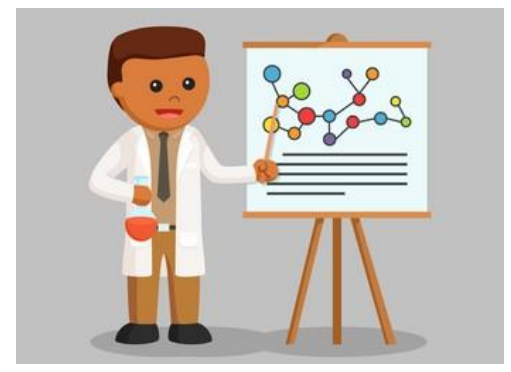

Pro Tip

It is common for an abstract to come prior to a presentation (or paper) to provide a summary of what to expect. Readers/reviewers will first look at the abstract to see if the rest of the story is worth even hearing!

Scientific Study Story to Share? → Scientific Abstract → Presentation / Article/Paper

---

The structure of your story should resemble an hourglass. You want to hook your audience with important broad concepts before going into the specifics of your study and then relating that back to the importance of the results broadly.

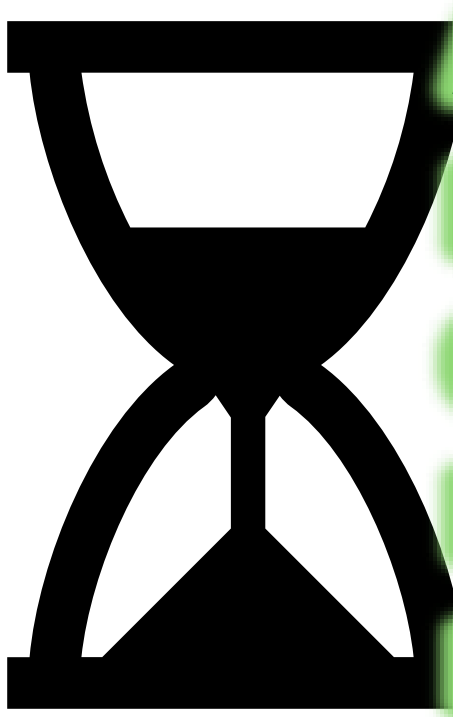

- **A** *Background /Introduction* → *Why should I care? Necessary background?* → *What is your question/hypothesis?*
- **B** *Methods* → *What methods were used to answer question?*
- **C** *Results* → *What answers did you find?*
- **D** *Discussion* → *What are the significance of the results? What should we gain?*
- **E** *Conclusions* → *What is the results' broader impacts?* → *What are future directions? (optional)*

Pro Tips

- Normally, these are written in past tense and passive (no "I", "me", "we", *etc.*) voice.
- Results and discussion can be combined.
- The background and "why we care" really depends upon your audience. It could be more scientifically-focused, meaning focused on scientific concerns, OR designed to highlight broader issues generally to the public.
- Normally, results/discussion & methods are shown off visually, not just with words (via a tables/figures & a visual abstract, respectively).

Generally (when building your story):

1. Make sure everyone in your group agrees and understands each part of the story/project.
2. The topics should flow, no matter if spoken or written.
3. All styles (written or visually) should be standardized and in a single voice.
4. Each point (slide, sentence, *etc.*) should have one focus. For example, there should be a clear separation between HOW you did the analysis and WHAT results you obtained.
5. Find balance between conclusions and providing enough quantitative information about the statistics that you convince the audience of the results.

How to Tell Your Story

(when writing abstracts or giving presentations)

For Abstracts:

1. The abstract of a story/article/presentation should convey to readers the most relevant aspects of each part of the whole story.
2. The abstract should be written by carefully selecting the words and sentences to provide coherent, clear, and concise contents. →
3. Do not use abbreviations, excessive acronyms, references, or figures.
4. It should be a freestanding paragraph (meaning no bullet points).

Example Wording:

- "We performed statistical analysis and saw the samples were different." (vs.)
- "Using a confidence interval (95% confidence, 4.3-5.6 ppm), we showed that the 2 samples are statistically different."

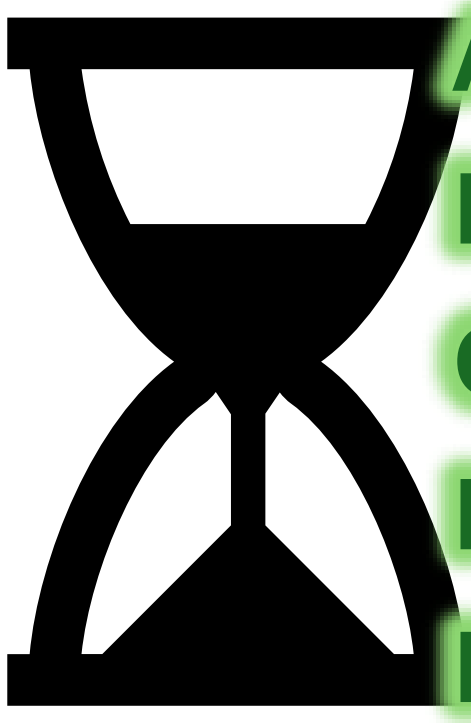

A *Background /Introduction*

B *Methods*

C *Results*

D *Discussion*

E *Conclusions*

Know Your Audience

Who your audience is matters a lot! Are you trying to share your work to a group of scientists or to the general public? Between the two examples below, note:

1. Level of background knowledge assumed
2. Types of language used/use of acronyms
3. How engagement is maintained

...But they still have the same structure!

Example Scientific Abstract:

A Browning index (BI, $ABS_{520\,nm}/ABS_{420\,nm}$) is a measure of anthocyanin-rich fruit juice pigmentation quality. This study sought to determine the extent to which BI describes anthocyanin quality and degradation in fruit juic B Commercial fruit juices were assayed for monomeric anthocyanin (MA) content, percent polymeric color (%PC), pH, and BI. BI varied, 0.29 to 1.72, among cranberry, cherry, grape, aronia, and pomegranate juic C Principal component analysis (PCA) revealed that BI was strongly inversely associated with %PC, and positively correlated with MAs to a lesser extent. The BI of grape and cherry juices varied linearly with pH from 2.0 to 4.0 in pH-adjusted juices. Cherry and grape juices at pH approximately 2.0 to 4.0 were incubated at 50 °C to induce juice browning. BI and MA decreased, and %PC increased, but the amount of MA degradation was not explained by %PC. In the aged juices, BI and MA were strongly correlated using P B In aged grape juice, chromatographic analysis was used characterize anthocyanins, proanthocyanidins, and anthocyanin scission produ C Anthocyanin loss and a gain of unresolved components absorbing at 420 nm decreased BI. Proanthocyanidins and co-eluting pigments with varying BI decreased during aging. Scission products did not account for anthocyanin lo D Thus, MA loss more so than the gain in pigments associated with juice proanthocyanidins contribute to the increase in %PC and decline of the BI during accelerated aging of grape jui E Thus, BI is a useful marker of fruit juice quality within juices of the same pH and anthocyanin composition.

Example Health Newsletter

A News articles, advertisements, and food labels often tout antioxidant benefits such as slowing aging, fending off heart disease, improving flagging vision, and curbing cancer. And laboratory studies and many large-scale observational studies (those that query people about their eating habits B and supplement use and then track their disease patterns) have noted antioxidant benefits from diets rich in them, particularly those coming from a broad range of colorful vege C bles and fruits. But results from randomized controlled trials of antioxidant supplements (in which people are assigned to take specific nutrient supplements or a placebo) B have not supported many of these claims. Indeed, too much of these antioxidant supplements won't help D you and C may even harm you. It is better to supply your antioxidants from a well-rounded diet. E

Pro Tip

Notice that that results/discussion are always followed by methods, but you can repeat that sequence multiple times!

How to Tell Your Story

(when writing abstracts or giving presentations)

For Presentations:

1. Prepare in advance! Practice the presentation, including transitions, who will introduce everyone, who will advance the slides, the time, *etc*. Practice will also reduce nerves, allow you to avoid reading from notes or from the slides, and make you more confident!
2. Don't forget to make eye contact (avoid staring at the slides!), project your voice, and use your voice as a tool to highlight the important items.
3. Think about questions in advance. Prepare backup slides if needed. Don't be afraid to ask for clarifications, and don't make up answers. Be honest and feel comfortable saying "that is a great questions, and we should look into that".
4. Avoid large amounts of text; remember mostly everything can be displayed visually instead. Find balance!

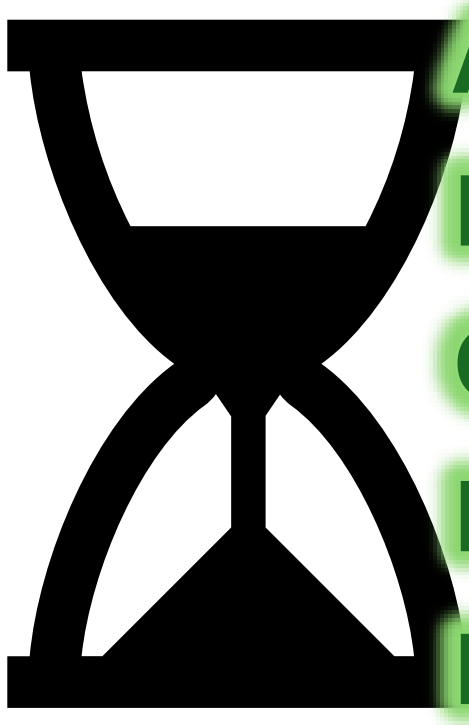

A *Background /Introduction*
B *Methods*
C *Results*
D *Discussion*
E *Conclusions*

Example Scientific Presentation

Class Slides

- How does your eye flow through the graphic?
- What is the background knowledge required?

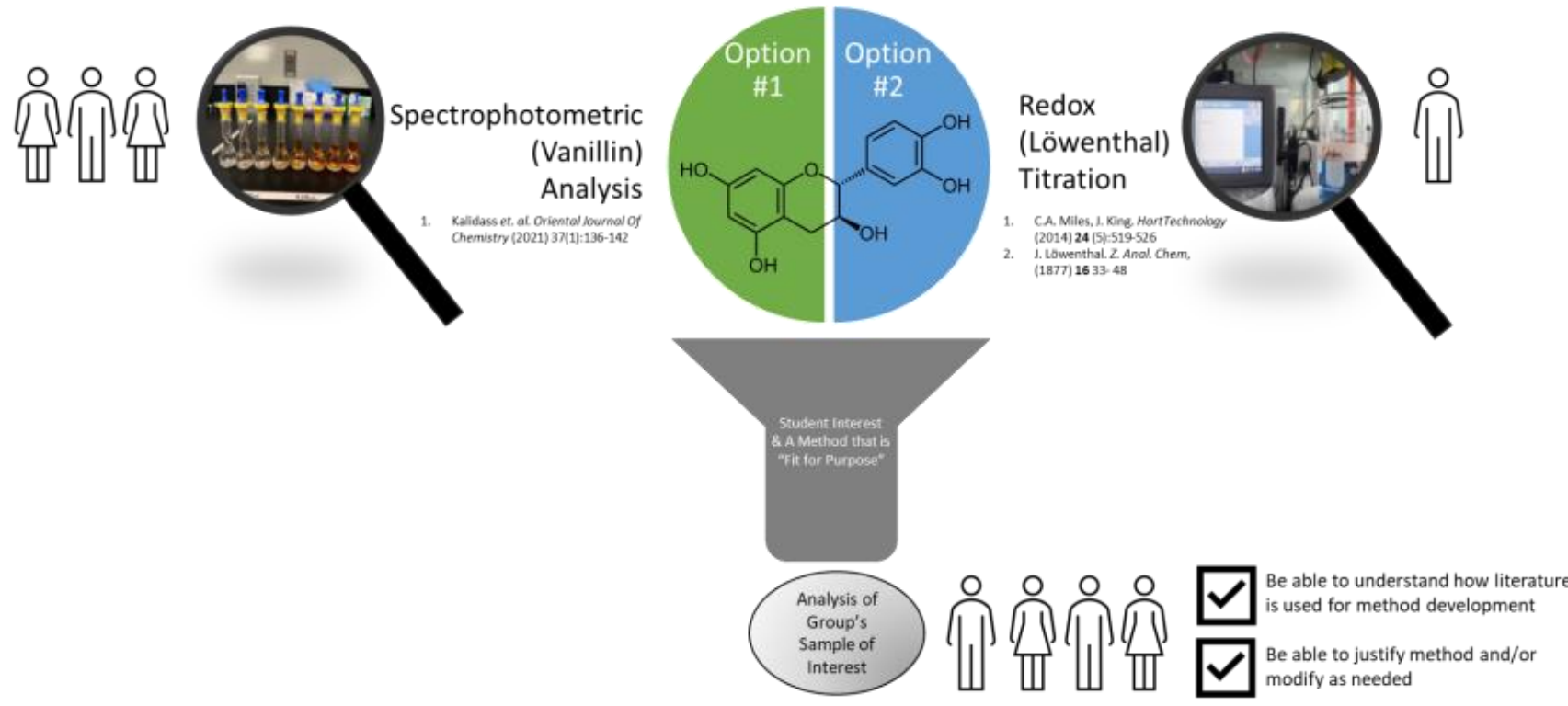

Know Your Format

There are many formats for presentations (PowerPoint/slides, poster, video). Notice differences between:

- Types of images/graphics
- How information is presented (visually, verbally, etc.)

Example General Public Graphic:

"Chemistry Of…" Graphics (Check these out via this link!)

- How does the figure grab your attention?
- Notice level of explanations, despite complex chemistry topics

Example Public Presentations:

YouTube Videos

Videos are examples of presentations! Check out how these videos present information in the same hourglass order. How do they use visuals?

Video 1 Video 2 Video 3

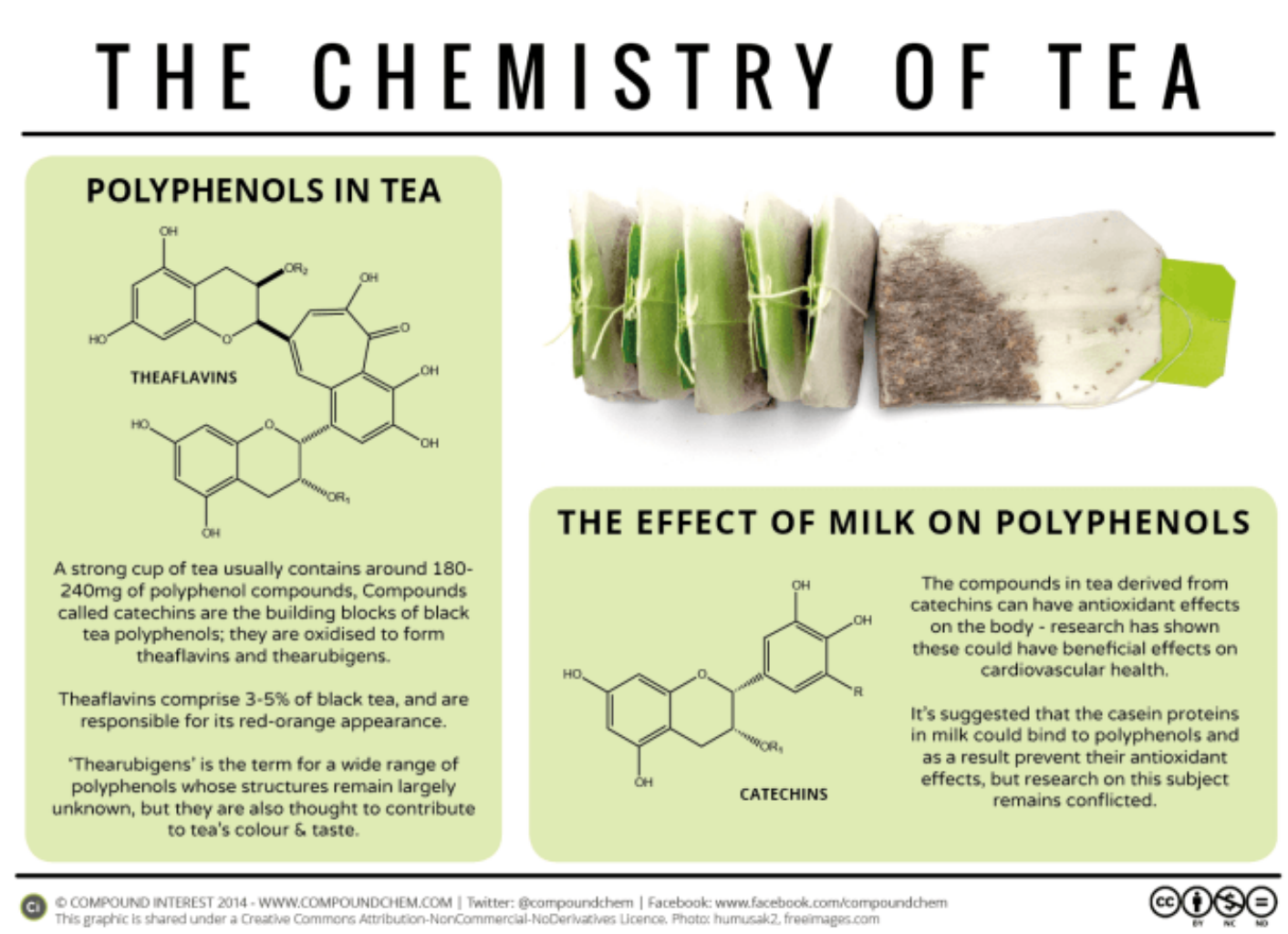

Pro Tip

UW Design lab has some great resources as a starting point for making a presentation.
