# Supplementary material for: Pick Your Own Adventure: Orthogonal Methods for Analyzing Polyphenols
Source: J Chem Educ. 2026 Jul 17;103(8):4243–54. doi: 10.1021/acs.jchemed.5c01459 (PMC13480120; doi:10.1021/acs.jchemed.5c01459)
Supplement: Supplementary file 2 [file ed5c01459_si_002.pdf]

Pick Your Own Adventure: Orthogonal Methods for Analyzing Polyphenols

Amanda Rae Buchberger[1]*, Kristel M. Forlano[1], Madeline J. Lund[1], Pamela S. Doolittle[1], Dominic M. Colosi[1], Thao U. Duong[1], Brian J. Esselman[1], Kyoung-Shin Choi[1], Robert J. Hamers[1]

[1]Department of Chemistry, University of Wisconsin-Madison, 1101 University Avenue, Madison, WI 53706

*Email Addresses:
amanda.buchberger@wisc.edu

Supporting Information/Instructor Notes

Table of Contents:

1. **Lowenthal Titration (LT)** S2
   - Special Chemicals/Equipment Used
   - Pre-lab Worksheet Questions to Prepare Students
   - Instructor Guidance
2. **Vanillin Spectrophotometry (VS)** S9
   - Special Chemicals/Equipment Used
   - Pre-lab Worksheet Questions to Prepare Students
   - Instructor Guidance
3. **Chromatography (CH)** S17
   - Special Chemicals/Equipment Used
   - Pre-lab Worksheet Questions to Prepare Students
   - Instructor Guidance
4. **Independent Sample Analysis (ISA)** S24
   - Instructor Guidance
5. **Activities/Assessments Connection to Scientific Practices & Industry-Prioritized Skills** S27
6. **Full Course Schedule for Spring 2024** S29
7. **Course Learning Objectives** S30
8. **Grading Rubrics** S31
   - Final Abstract Grading Rubric
   - Final Presentation Rubric
9. **Survey Questions** S34
10. **References** S40

1. **<u>Lowenthal Titration (LT)</u>**

<u>Special Chemicals/Equipment list</u>

Equipment Used:

- Balance capable of massing 10 mg
- Chimeric stirring hot plate
- Volumetric flasks (*i.e.,* 100 mL, 250 mL)
- Volumetric pipets (*i.e.,* 5 mL, 25 mL)
- Erlenmeyer flasks or beakers (*i.e.,* 250 mL)
- Buret (*i.e.,* 50 mL) and stand/clamp
- Data Collection Platform
  - Example: Venier LabQuest 3 (*Order Code: LABQ3*)
- Oxidation-Reduction Potential (ORP) Probe
  - Example: Vernier ORP Sensor (*Order Code: ORP-BTA*)
- Drop Counter
  - Example: Vernier Drop Counter (*Order Code: VDC-BTD*)

Special Chemicals & Instructions:

- (+)-Catechin Hydrate (*Aldrich 22110-100G*)
  - Air sensitive. Solution should be made fresh each lab period and remain covered when not in use.
  - Supplied in 40-50 mg aliquots (1 per student). Stored in capped vials between portioning and giving to students.
- Gallic Acid (*ChemCruz sc-205704B*)
  - Air sensitive. Solution should be made fresh each lab period and remain covered when not in use.
  - Supplied in 40-50 mg aliquots (1 per student). Stored in capped vials between portioning and giving to students.
- Potassium Permanganate ($KMnO_4$) (*Sigma-Aldrich 223468-500G*)
- Indigo Carmine (indicator) *(TCI I0214)*
- Sulfuric Acid
  - Supplied at 50% for students to dilute.

Pre-lab Worksheet Questions to Prepare Students

The key can be provided by request.

Purpose: The following activity is meant to help you think about the data analysis for the Lowenthal Titration portion of the project lab.

Learning Objectives:

- *Understand what information statistical tests provide.*
- *Critically analyze published statistics to draw conclusions.*
- *Draw analogies to statistical analysis that will be completed in the project lab.*

In the paper *Comparison of Common Analytical Methods for the Quantification of Total Polyphenols and Flavanols in Fruit Juices and Ciders* (DOI: 10.1111/1750-3841.14713)[1] the authors are completing a very similar set of experiments to what you will be doing throughout the semester! In this study, they compare the amount of polyphenols they obtain through 4 different analytical methods: Lowenthal-Permanganate (L-P) Titration, Folin-Ciocalteu (F-C) assay, 4-dimethylaminocinnamaldehyde (DMAC) assay, and the bovine serum albumin (BSA) precipitation. Both the F-C and DMAC methods are spectroscopic analyses, similar to the one you'll be completing in the Vanillin Assay portion of the project lab. The authors determined the method parameters, validated each method, and then completed real sample analysis on 14 fruit juice samples, 22 apple juice samples, and 17 cider samples. Wow, that's a lot of data! We will be using some of their results to help guide us through thinking about the data analysis for your own experiments.

In the first part of the study, the authors completed a method validation. The results they give are in Table 2 in the manuscript linked above.

1. We are told that the measurements were completed on a gallic acid or other standards of known concentration, and that each sample was measured "10 times each with a 15-min interval on the same day under the same conditions (materials, equipment, and temperature) by the same personnel". Are the authors testing *Reproducibility* or *Repeatability*? How does that compare to the experiments you're completing? What conclusions will you be able to draw versus the paper?

2. The authors report Relative Standard Deviation (RSD) (Equation 3 in manuscript), which is a form of the standard deviation according to the equation below (you can check your textbook that expresses the standard deviation as a percentage of the mean). Does this measure the accuracy or precision of the measurement? Is this useful for you to consider for your data?

3. What type of calculation is the recovery (Equation 4 in manuscript)? Maybe you know this calculation by a different name? Does this measure the accuracy or precision of the measurement? The L-P method has a large recovery, despite it being a very repeatable method. What could be causing this? Would you expect a similar effect in your own experiments?

4. The authors give a 95% confidence interval (CI) for their data in Table 2. They also give the known concentrations of the standards they were measuring: 300 mg/L, 5 g/L, and 20 mg/L for the F-C, L-P, and DMAC methods respectively. What do you notice about the "known" value versus the CI? Can you state this statistically? (*i.e. There's a 5% chance of…*) What does this tell you about the accuracy of the measurement?

In a later part of the study, the authors determine the quantity of polyphenols in real samples of different fruit juices (see Table 4 in manuscript).

5. If you're comparing the polyphenol content of one type of juice by the two analytical methods, how would you go about statistically comparing the results (*i.e. What tests*)? What would the results of those tests allow you to conclude about the data? Is this a similar or different process to how you'll compare your data to the data of your other group members? As practice, you can try out the statistical calculations comparing the two methods. Which samples had statistically significant different polyphenol content between the two methods?
   PS. Your lab manual has a flow chart of how to decide what statistical test to use!

6. In your lab manual, Results/Calculations questions 2, 4, and 5 all ask for you to complete statistical analysis of your results and to <u>justify</u> which statistical tests you're doing. You can already determine which statistical tests would be appropriate to use, even if you don't have results yet! Discuss and plan with your group.

<u>Instructor Guidance</u>

- **Pre-lab Talk Notes**
  - Much of this lab is in your hands. Be prepared for me (the instructor) to not know the exact answer to your question.
  - SAFETY IS OUT BIGGEST CONCERN. WEAR PPE (GLOVES, GOOGLES, LAB COAT). All H_2SO_4 should be disposed of down the sink with A LOT of water (turn on the sink first, and then pour any excess down the drain slowly) after being neutralized.
  - If you would like to use glassware that is not found in your drawer, ask the stockroom if they have options that work for you.
- **General Expected Schedule**
  - Students will need to do a minimum of 1 automated titration on Day 1 (in a group), 9 titrations on Day 2 (individually), and 6 titrations on Day 3 (individually). They can likely do the 9 titrations with the automated titration in 1 day if they wish. A sample schedule is provided in Table S1.

Table S1. Sample Schedule for LT Experiment. An “x” means task is completed on that lab day.

Task?	Day 1	Day 2	Day 3
(Making of) Key Solutions	x	x	x
Determination of Titration Endpoint	x		
Quantitative Analysis of Standard Samples	x	x	x
Analysis for Reproducibility (Results/Calculations)			x

- **Implementation Considerations & Sample Results**
  - Besides the student-provided materials, TAs were provided a pre-built pre-lab PowerPoint, representative sample data, a macro for calculating the expected values based upon the student data, and an overview of the common mistakes/modifications students could make. *All of these materials can be provided by request*.
  - Student groups of 3-4, maximizing 3 students first.
  - To make the ~0.005 M $KMnO_4$ solution, students should weigh out 0.2 grams in a 250.0 mL volumetric flask. Students will figure out that this is too concentrated and likely need to dilute their $KMnO_4$ 10x to get within the ideal range of a 50 mL buret.
  - To make the indigo carmine solution, they can add 0.1 grams into a 100.0 mL volumetric flaks with 10 mL of 50% H_2SO_4 prior to diluting to 100.0 mL. The indicator is just to see the color change, so students could weigh out less (~0.05% solution) be successful.

- Solids can form during the titration if the pH is not controlled due to the multiple different reactions that can occur with MnO_4^-. This is not an issue during this lab module, but it has been observed when working with basic samples. The color will not change as well without sulfuric acid. (Figure S1A).
  - For the blank titration, a color change should occur prior to 10-20 mL added. Blank titration should be less than the titration with gallic acid or catechin (Figure S1B).
  - The expected color is green-yellow. Here is a link to a video of the titration color evolution. The titration will go through the rainbow of colors (starting at blue and going all the way to red if you add an excess amount of $KMnO_4$.)
    - Indigo carmine (also known as FD&C Blue #2) is a pH sensitive indicator that blue in acidic conditions and yellow in basic conditions. The $KMnO_4$ is purple/dark pink. Once the polyphenols are reduced, the indicator begins being reduced to a yellow color (moving through teal and green before yellow). The addition of $KMnO_4$ to the yellow slowly creates an orange solution followed by red/pink mixture (due to a mix of the purple/pink & yellow).

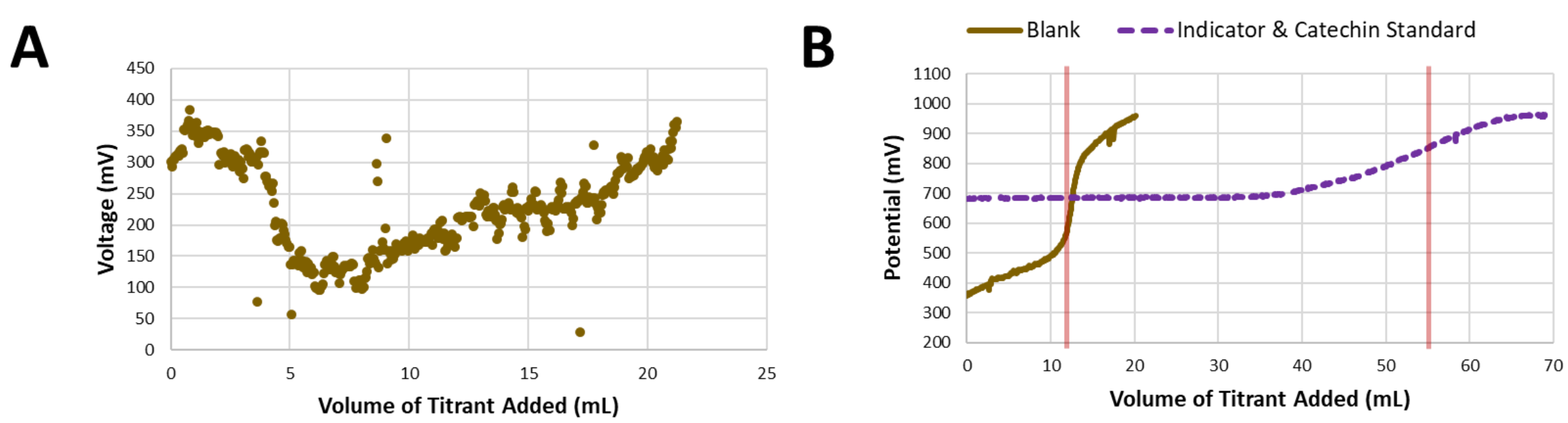

Figure S1. Instructor-collected example data. (A) LT experiment performed without the addition of sulfuric acid. Due to the lack of acid, the reduction-oxidation reaction does not work as intended. The indigo carmine indicator is also pH sensitive, leading to more confounding color changes. (B) An example titration curve of both the blank (with just the indicator) and the indicator with a catechin standard under acidic conditions. As expected, it takes more volume of titration to reach the equivalence point (as highlighted by the red line) than the blank. The baselines are slightly different due to not calibrating the ORP probe before use.

- The ORP probes are slower to equilibrate than pH probes; thus, extra care needs to be taken to make sure they have enough time to equilibrate, especially since they are not calibrated in this experiment.
  - We are not calibration them because we do not really care about the exact voltage; instead, the titration is looking for where there is the

largest change. If there is desire, you can calibrate the ORP probe prior to use.
    - Some ORP probes can be changed over to read pH; we do not want to read pH in the experiment, and, thus, this can lead to issues with results.
- The students need to be careful to add a specific volume of the polyphenol (catechin or gallic acid) and the indicator, as both can impact the endpoint volume. Meaning, they should use volumetric glassware and NOT a graduated cylinder. In contrast, the exact volume in which the sample is diluted to with DI water doesn't change the chemistry at all. It just impacts the clarity of the color.
- Endpoint will be between green and yellow, depending upon the amount of water. THEY NEED TO WATCH THIS CAREFULLY. THEIR EYES NEED TO SEE WHEN THE COLOR CHANGES.
- Students will likely find a statistical difference between the catechin and gallic acid titrations ratios. Hopefully they find no difference between their titrations and their classmates' titrations. These can be confirmed via a 2-sample t-test. Example student data is included in Table S2. *Raw data can be shared upon request.*
    - From past classes....

Table S2. Example Class Results for Tannic Acid Equivalents (TAE) and Mole-to-Mole Ratio

	Fall 2023	Spring 2024
Catechin TAE	418.3 ± 440.6 (±97.4; 548.6)	213.7 ± 220.6 (±39.2; 67.7)
Gallic Acid TAE	512.1 ± 541.3 (±120.4; 751.8)	188.8 ± 137.7 (±35.0; 76.6)
Catechin-to-$KMnO_4$ Ratio	0.53 ± 1.07 (±0.24*; 0.41)	0.62 ± 0.66 (±0.12*; 0.18)
Gallic Acid-to-$KMnO_4$ Ratio	1.07 ± 2.90 (±0.64*; 0.62)	0.56 ± 0.47 (±0.12*; 0.21)

NOTE: The numbers in the parentheses are the (1) 95% confidence interval and (2) interquartile range (IQR). If there is a statistical difference for the mole-to-mole ratios from the expected ratio (2.5), the interval will be marked with an asterisk (*).

    - From instructor testing...
        - Average Mole-to-Mole (Catechin-to-$KMnO_4$) Ratio: 1.50 ± 0.03
        - Average Mole-to-Mole (Gallic Acid-to-$KMnO_4$) Ratio: 1.05 ± 0.05
- If standardization of the $KMnO_4$ is performed, it is recommended that the students are given an extra lab day (meaning 4 days total) and only have

catechin or gallic acid analyzed for reproducibility and repeatability. An alternative is to have the instructor or staff pre-standardize the $KMnO_4$ to avoid adding more lab time for students.
    - If the instructor provides the $KMnO_4$, students can "recycle" $KMnO_4$ by returning undiluted $KMnO_4$ for future students to use.
- Another way to explore redox chemistry of polyphenols is using Pourbaix diagrams, which was done in one of the earlier versions of this lab experience.
- If automated titration systems are not available, a truncated form of the LT can be done just considering the reproducibility/repeatability of the lab module while provided the expected endpoint color change.
- If alternative redox titrations are required due to the sample background, I_2 as the oxidant followed by a back titration with $Na_2S_2O_3$ is a viable alternative.[2]

Table S3. Example LT Mole-to-Mole (Polyphenol-to-$KMnO_4$) Ratio Results

Student #	Replicate	Catechin (Day 1)	Catechin (Day 2)	Gallic Acid
A*#	1	1.21	2.06	1.45
	2	1.27	2.01	1.41
	3	1.25	1.98	1.4
	Average	1.24	2.02	1.42
B*#	1	0.47	0.59	14.1
	2	0.48	0.59	15.1
	3	0.48	0.57	14.9
	Average	0.47	0.59	14.7

*Statistical difference between Day 1 vs. Day 2 comparison of same student.

#Statistical difference between student A and B comparison.

2. <u>**Vanillin Spectrophotometry (VS)**</u>

<u>Special Chemicals/Equipment list</u>

Equipment Used:

- Balance capable of massing 10 mg
- Volumetric flasks (*i.e.,* 10 mL, 100 mL)
- Volumetric or Mohr pipets (*i.e.,* 1 mL, 2 mL, 3 mL)
- Variable, single-channel pipet (*i.e.,* 100-1000 uL)
- Graduated cylinder (*i.e.,* 10 mL)
- Data Collection Platform
    - Example: Venier LabQuest 3 (*Order Code: LABQ3*)
- Spectrophotometer capable of UV-Vis detection
    - Example: Vernier Go Direct® SpectroVis® Plus Spectrophotometer (*Order Code: GDX-SVISPL*)

Special Chemicals & Instructions:

- (+)-Catechin Hydrate (*Aldrich 22110-100G*)
    - Air sensitive. Solution should be made fresh each lab period and remain covered when not in use.
    - Supplied in 40-50 mg aliquots (1 per student). Stored in capped vials between portioning and giving to students.
- Gallic Acid (*ChemCruz sc-205704B*)
    - Air sensitive. Solution should be made fresh each lab period and remain covered when not in use.
    - Supplied in 40-50 mg aliquots (1 per student). Stored in capped vials between portioning and giving to students.
- Sulfuric Acid
    - Supplied at 70% for students to use.
    - This should be made in advance to allow it to cool to room temperature if mixed from concentrated (100%) sulfuric acid.
- Green Tea Tablet
    - Example: Nature's Bounty Green Tea Capsules (*found at CVS*)
- NOTE: Other chemicals we have considered for students or have been asked for include:
    - Curcumin
    - Quercetin
    - Anthocyanidins

Pre-lab Worksheet Questions to Prepare Students

The key can be provided by request. NOTE: Dr. Bolling is a faculty member at the University of Wisconsin-Madison in Department of Food Science. Prior to this pre-lab activity, Dr. Bolling visited the lecture to give context to food science field and polyphenols.

Purpose: The following activity is meant to help you think about how sample choices and preparation may impact your quantitation during Vanillin portion of the project lab.

Learning Objectives:

- *Explain why polyphenol content is reported in equivalents.*
- *Recognize and apply quality assurance methods.*
- *Understand the effect of the solution matrix on collected data.*

In the paper *Assay Dilution Factors Confound Measures of Total Antioxidant Capacity in Polyphenol-Rich Juices* (DOI: 10.1111/j.1750-3841.2011.02538.x)[3], Dr. Bolling and his co-authors encounter a troubling issue: the Folin-Ciocalteu (FC) method that they are using is not consistent! The FC spectrophotometric method for determination of polyphenol content is the most common analytical technique used in polyphenol research and industry. This method is very similar to the Vanillin Assay you will be completing, so we will use this paper to examine something interesting you may run into!

They use a lot of abbreviations in this paper, so here's a key:

DF =	Dilution Factor
FC =	Folin-Ciocalteu
GAE =	Gallic Acid Equivalents
TP =	Total Polyphenols
GJ =	Grape Juice
PJ =	Pomegranate Juice
PNJ =	Pomegranate Nectarine Juice

In the paper, the authors create a calibration curve from the FC method using **gallic acid** standard solutions. *(Note that we will use catechin with the Vanillin method.)* They claim to be using the linear region of the calibration curve and report upper and lower concentration bounds. They don't show this data, so we'll have to trust them! In the first part of the paper, they make several other solutions using common polyphenol standards (quercetin, naringenin, catechin, and luteolin – all flavonoids!)) and calculate the **gallic acid equivalents (GAE)** from their calibration curve. For the next questions, please reference Figure 2 in the manuscript.

1. Let's break down this graph. It's trickier than it first appears! What quantity is expressed on the x-axis? On the y-axis? (PS. This isn't a calibration curve!)
2. Why are the authors calculating the polyphenol content of all solutions as gallic acid equivalents as opposed to total polyphenols (or gallic acid alone)? When you build your calibration curves, what will you be calculating?
3. Reproduce the graph and mark on the graph above what line you'd expect if GAE was a perfect representation of flavonoid concentration. Does this make sense?

In the second half of the paper, the authors test how measuring the polyphenol content of three samples (grape juice, pomegranate juice, and pomegranate nectarine juice) changes depending on the dilution factor. The Figure 3A in the manuscript shows the calculated, undiluted concentrations of the original juice from their measurements. We are told that all samples still fall into a linear range of the calibration curve.

4. In an ideal world, should the amount that a sample is diluted for measurement affect the calculated polyphenol content of the original sample? How would you expect the graph to look in an ideal world? What may be a reason for this effect?
5. In the Vanillin Assay lab, you will be using several quality assurance methods to determine the accuracy and reliability of your measurements. These include a method detection limit, a positive control, and a spike recovery. Choose a quality assurance method and brainstorm how you would apply it to test the validity of the results above.

Instructor Guidance

- **Pre-lab Talk Notes**
  - Much of this lab is in your hands. Be prepared for me (the instructor) to not know the exact answer to your question.
  - SAFETY IS OUT BIGGEST CONCERN. WEAR PPE (GLOVES, GOOGLES, LAB COAT). All H_2SO_4 should be disposed of down the sink with A LOT of water (turn on the sink first, and then pour any excess down the drain slowly) after being neutralized.
  - If you would like to use glassware that is not found in your drawer, ask the stockroom if they have options that work for you.
  - You are working as a team; make sure you delegate tasks like a team.
- **General Expected Schedule**
  - Students should divide up tasks rather than all students working on a specific thing. For example, students can split up making the vanillin, catechin, and gallic acid solutions. Then, students can create an assembly line to create solutions for testing purposes, making the calibration curves, *etc.* A sample schedule is provided in Table S3.

Table S4. Sample Schedule for VS Experiment. An "x" means task is completed on that lab day.

Task?	Day 1	Day 2	Day 3
(Making of) Key Solutions	x	x	x
Explore Vanillin Solubility	x		
Explore Polyphenol Reactiveness with Vanillin	x		
Determination of λ_{max}	x		
Define Calibration Range		x	
Analysis of Real Sample		x	x

- **Implementation Considerations & Sample Results**
  - Besides the student-provided materials, TAs were provided a pre-built pre-lab PowerPoint, representative sample data, and an overview of the common mistakes/modifications students could make. *All of these materials can be provided by request.*
  - Student groups of 3-4, maximizing 3 students first.
  - Students should weigh out ~1 gram of vanillin for each solution. For the 70% H_2SO_4 solution, that means the whole 100 mL is 70% H_2SO_4. For the 90:10 solution, 10 mL should be ethanol and 90 mL (diluted to the line of the volumetric flask) is 70% H_2SO_4.

- The vanillin should be put on ice once made, but the experiment seems to work well enough if vanillin isn't chilled.
- Once made, the 70% H_2SO_4 solution should look electric yellow. If the vanillin crashes out, or the solution is a very dull yellow, it is likely something was done wrong.
- Once made, the ethanolic version of the vanillin should be more yellow-brown.
- Since vanillin is in excess, you can use a graduated cylinder to measure this in all cases. It is important to be CONSISTANTLY adding the same amount, if possible. A difference of <0.25 mL has not shown a large difference. Another alternative is a 10 mL Mohr pipet.
- Vanillin, when stored at 4°C long term, can change colors. If dissolved in the ethanol solution, it will turn purple. If stored in the non-ethanol solution, it will turn green. The reactivity of vanillin does not change (based upon our observations/experiments), but it is recommended to make the solution fresh each day just in case (Figure S2).

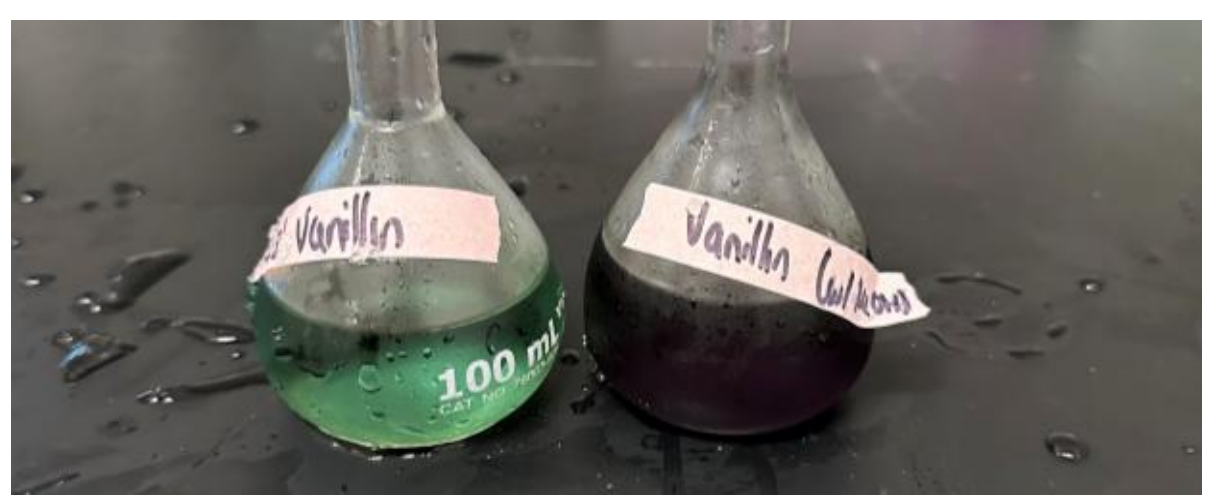

Figure S2. Example colors of aged vanillin solutions. The vanillin made with 70% H_2SO_4 only is on the left. The vanillin made with 70% H_2SO_4 and EtOH is on the right.

- A sample dilution plan for the standards is provided below in Table S4.

Table S5. Sample Dilution Plan for Determining Linear Range of Vanillin Assay

Final Concentration of Polyphenol Standard (mg/L)	1.0	2.5	5	10	15	20	25	30
Polyphenol Standard Added (mL)	0.1	0.25	0.5	1	1.5	2	2.5	3
Vanillin Added (mL)	6.5	6.5	6.5	6.5	6.5	6.5	6.5	6.5
Final Volume Diluted to (mL)	10	10	10	10	10	10	10	10
Polyphenol Standard Added (mL)	0.1	0.25	0.5	1	1.5	2	2.5	3
Vanillin Added (mL)	6.5	6.5	6.5	6.5	6.5	6.5	6.5	6.5

- Gallic acid will not react with vanillin (Figure S3), while catechin will react with vanillin with a λ_{max} of ~500 nm.
  - Reactivity does not change with solvent (Figure S4A and S4C).
  - The ~390 nm peak is vanillin alone (Figure S5).

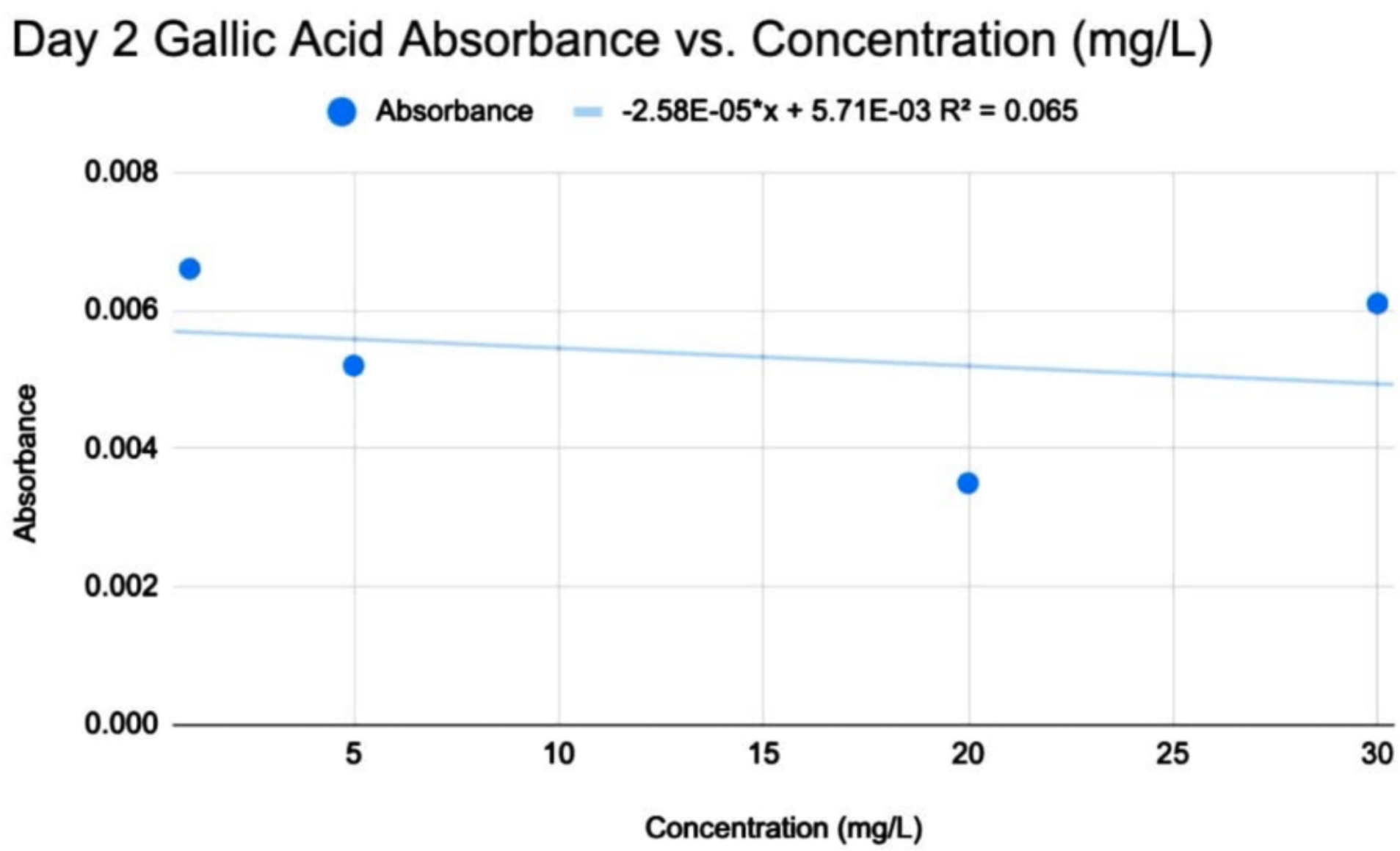

Figure S3. Example student data generating a gallic acid calibration curve via the VS method. Gallic acid does not react with vanillin.

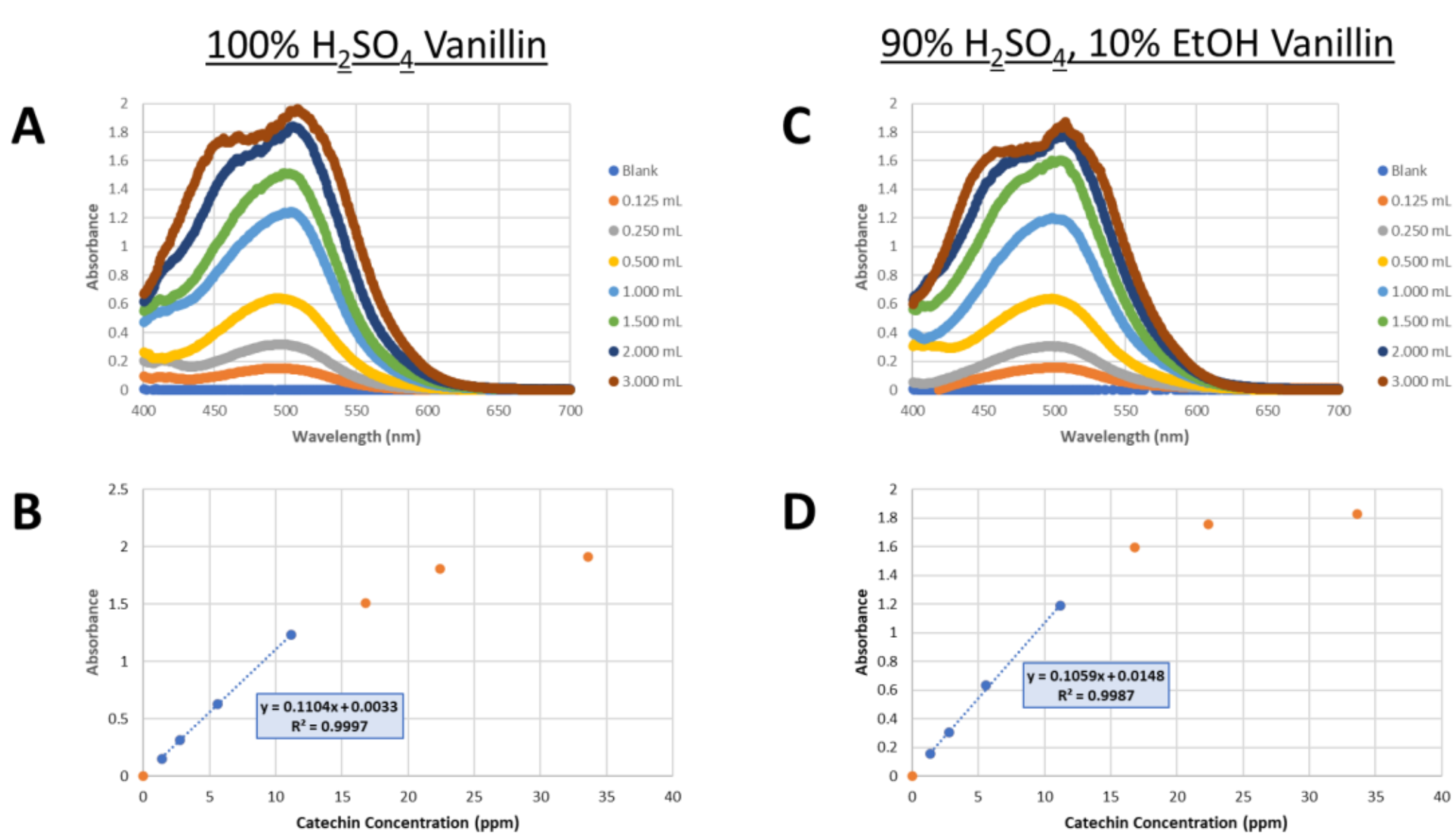

Figure S4. Instructor comparison of the different solvent choices for the VS experiment. The spectrophotometer was blanked with a reagent blank (vanillin in appropriate solvent mixed with DI water).

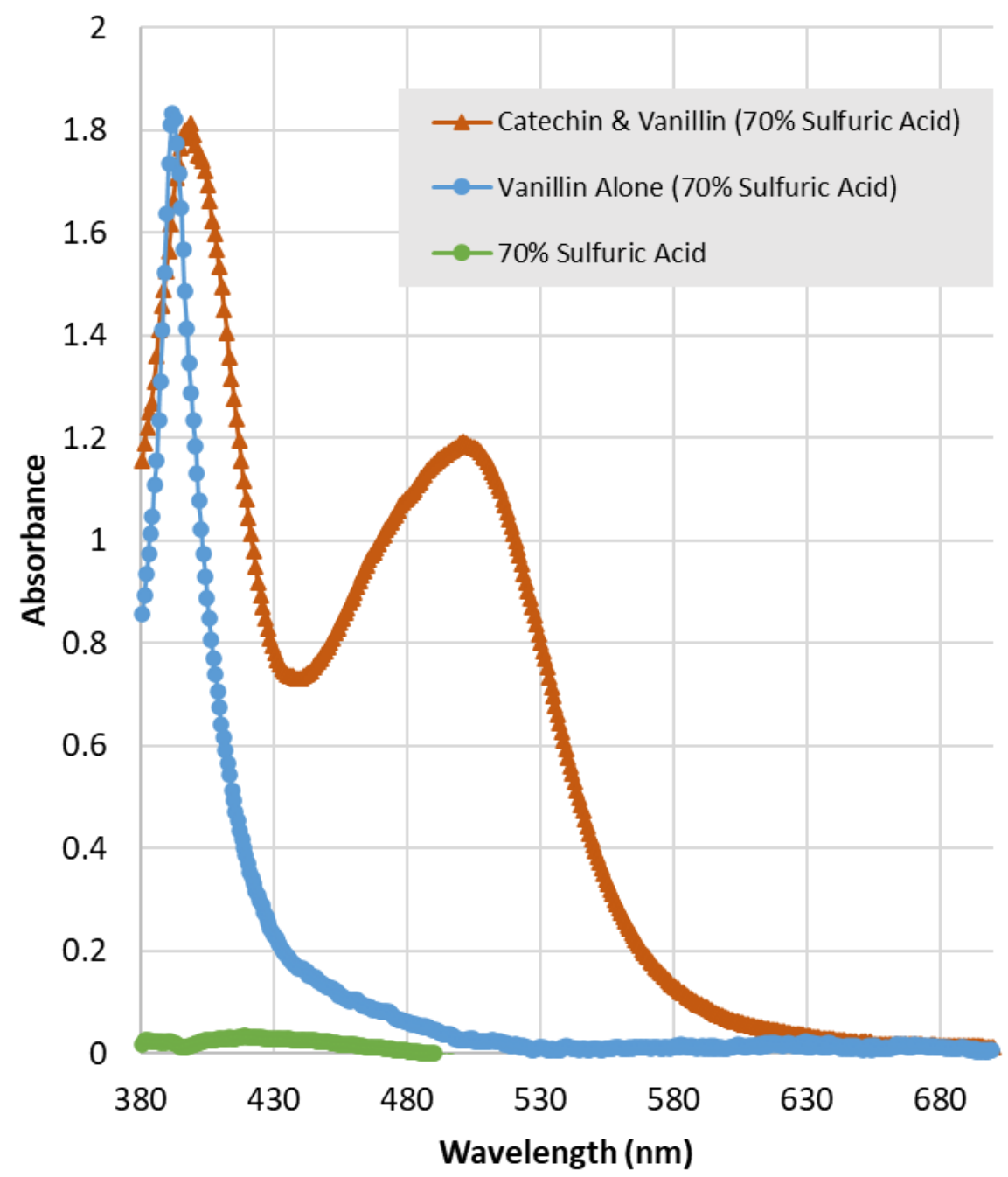

Figure S5. Measurement of 70% sulfuric acid, vanillin in 70% sulfuric acid, and catechin with the vanillin in 70% sulfuric acid to demonstrate which peaks are associated with the solvent, vanillin alone, and catechin reacting with the vanillin. The spectrophotometer was blanked with DI water.

- For our instruments, we have found a reasonable linear range for catechin between 0.5 and 10 ppm Figure S4B and S4D). The lower range for most students was between 0.2-1.0 mg/L, and the upper range was 10-15 mg/L. (Figure S5).

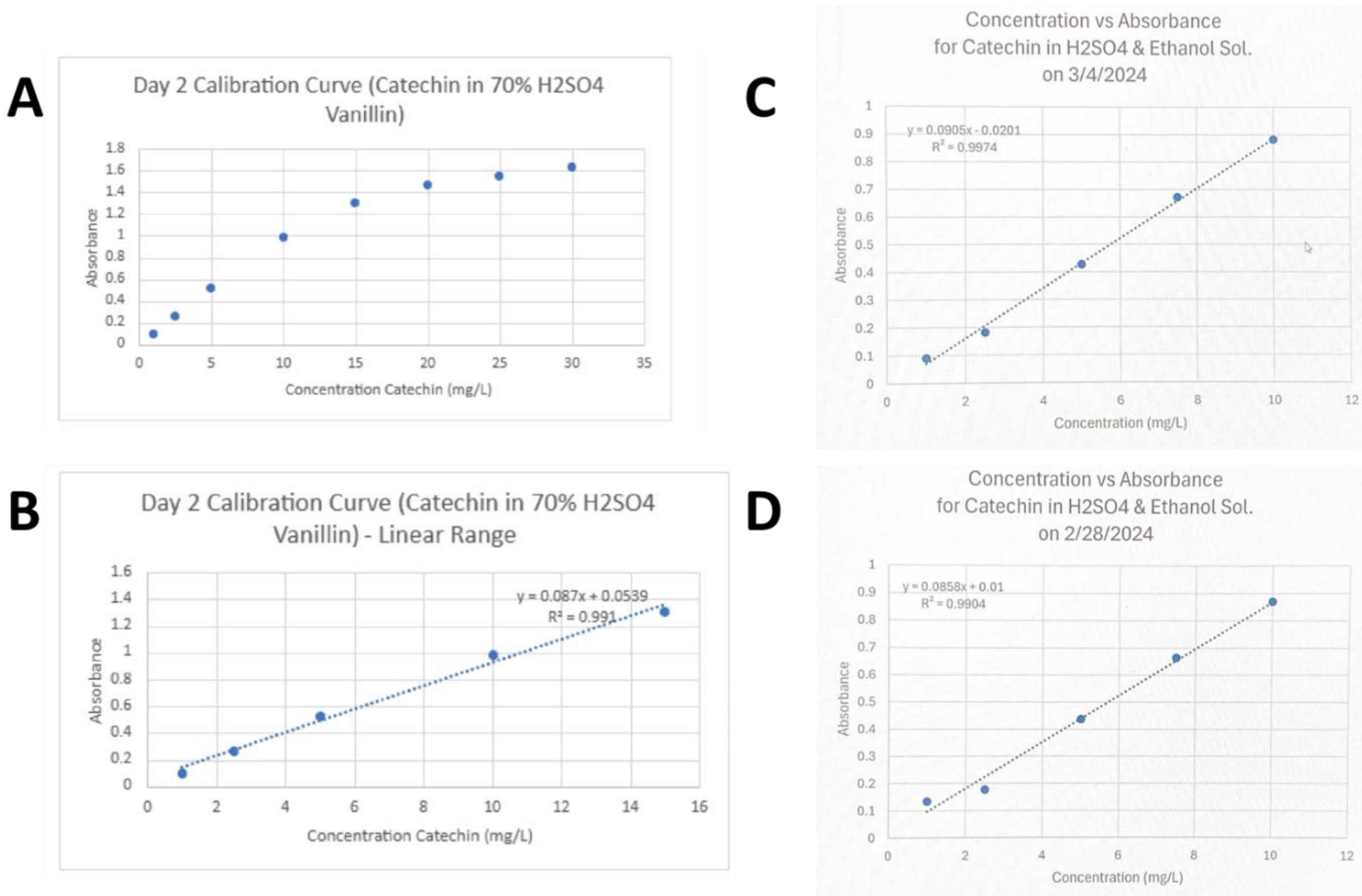

Figure S6. Screenshots of student VS lab report data, including determination of the dynamic range (A) and linear range (B) of catechin reacting with vanillin. These calibration curves were constant day-to-day (C vs. D).

- For the MDL, 0.01 ppm is common for our instruments.
- For the green tea tablets, the powder is easy to remove from the gelatin casing.
    - The matrix of the tablet can be problematic. The tablet is orange/red colored (which could interfere with the vanillin assay). Depending upon how students dilute the powder, they could get between 20-200% spike recovery. If they dilute the powder more, they will see less deviation from 100%; it is well documented that different dilution schemes will cause differences in the recovery. The prelab activity on pages S11-13 helps prepare students to think of these dilution issues.
    - Spike added for the spike recovery should be 0.1-0.2 mL (if preparing 10 mL of solution) to avoid changing the matrix.

3. <u>Chromatography (CH)</u>

<u>Special Chemicals/Equipment list</u>

Equipment Used:

- Balance capable of massing 10 mg
- Volumetric flasks (*i.e.,* 100 mL)
- Volumetric or Mohr pipets (*i.e.,* 1 mL, 2 mL, 3 mL)
- Variable, single-channel pipet (*i.e.,* 100-1000 uL)
- Filtering apparatus (*i.e.,* Buechner funnel, 1000 mL filter flask, and Q5 filter paper)
- HPLC unit with manual injector, isocratic pump, and UV-Vis detection
    - Example: Shimadzu 10 Series
- uHPLC unit with automated injector, multiple pumps (for gradient elution), and both UV-Vis and fluorescence detectors
    - Example: Shimadzu Nexera XR Series
- 4°C Fridge (if storing tea samples)

Special Chemicals & Instructions:

- (+)-Catechin Hydrate (*Aldrich 22110-100G*)
    - Air sensitive. Solution should be made fresh each lab period and remain covered when not in use.
    - Supplied in 40-50 mg aliquots (1 per student). Stored in capped vials between portioning and giving to students.
- Gallic Acid (*ChemCruz sc-205704B*)
    - Air sensitive. Solution should be made fresh each lab period and remain covered when not in use.
    - Supplied in 40-50 mg aliquots (1 per student). Stored in capped vials between portioning and giving to students.
- Green Tea Bag Sample
    - Example: Ahmad Tea Londen Green Tea
- Black Tea Bag Sample
    - Example: Lipton Black Tea
- <u>NOTE</u>: If cost is not an issue, other chemicals of interest include:
    - Gallocatechin
    - Epigallocatechin
    - Epicatechin
    - Epigallocatechin Gallate
    - Gallocatechin Gallate

- Epicatechin Gallate

Pre-lab Worksheet Questions to Prepare Students

The key can be provided by request.

Purpose: The following activity is meant to help you think about how to analyze HPLC chromatograms.

Learning Objectives:

- *Understand and apply the chemistry behind HPLC.*
- *Use standard addition to identify chromatographic peaks.*
- *Calculate polyphenol content from HPLC data.*
- *Understand the effects of interfering molecules on HPLC data.*

In the paper *Browning Index of Anthocyanin-Rich Fruit Juice Depends on pH and Anthocyanin Loss More Than the Gain of Soluble Polymeric Pigments* (DOI: 10.1111/1750-3841.14106)[4], Dr. Bolling and co-authors examine how the Browning Index (BI) is related the pH and content of fruit juices. The BI is a metric that has been used to describe the quality of fruit juices and is calculated through an absorbance measurement ($ABS_{520\ nm}/ABS_{420\ nm}$). However, the metric had not been well correlated with the chemistry that was happening in the fruit juice. In this paper, the authors attempt to define changes in the BI through changes in polyphenol content.

One of the fruit juice samples, GrZc11, was analyzed through HPLC. The text is dense, but we can see all of the information we need about how they ran their experiments. The text describes how the samples were prepared, the instrument specifications, how the column was run, what data was collected, and how the chromatographic peaks were identified. Wait. "Data not shown"?!

Well, this can actually be a common issue when reading journal articles; sometimes not all data is given. Luckily, research is a very collaborative field and scientists frequently reach out to each other to ask questions and request data. We reached out to request the "data not shown" and the authors graciously went back and compiled the chromatograms that he used to identify different polyphenols. We'll be taking a look at some of this data today.

1. There are two specific polyphenols that will be identified in the chromatograms: gallic acid (GA) and protocatechuic acid (PA or PCA). Reverse-phase HPLC is used to separate the compounds. Look up the structures and determine what the elution order should be and justify.

Consider the elution conditions for the column. Of the three solvents mentioned, polarity decreases from water > methanol > formic acid, although all are polar. Graph the %B in the elution mixture as a function of time. At what point is the elution mixture most polar? Least polar? Why would it be beneficial to run a gradient column like this with all polar solvents? PS. Mixture A is mostly water!

2. The authors use standard addition to identify which peaks in the juice chromatogram correspond to standard polyphenols. Dr. Dorris provided us with the data below. Describe how the shown data and the standard addition method helps to identify chromatogram peaks. It may help to define what standard addition is and how the experiment would be conducted in HPLC.

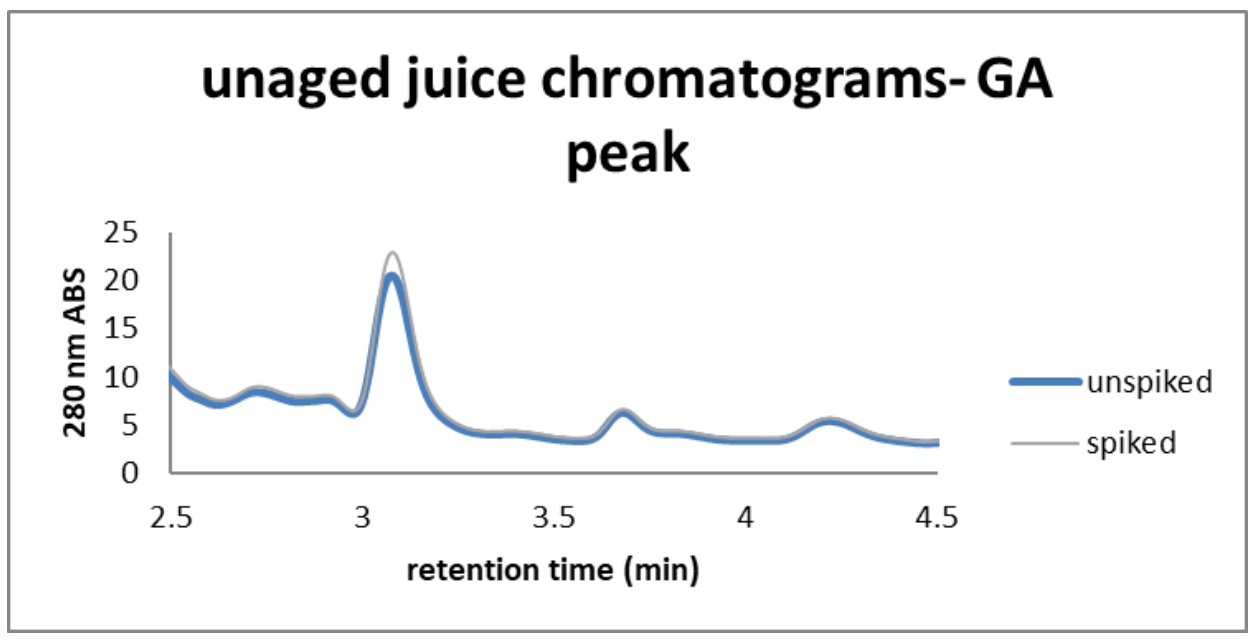

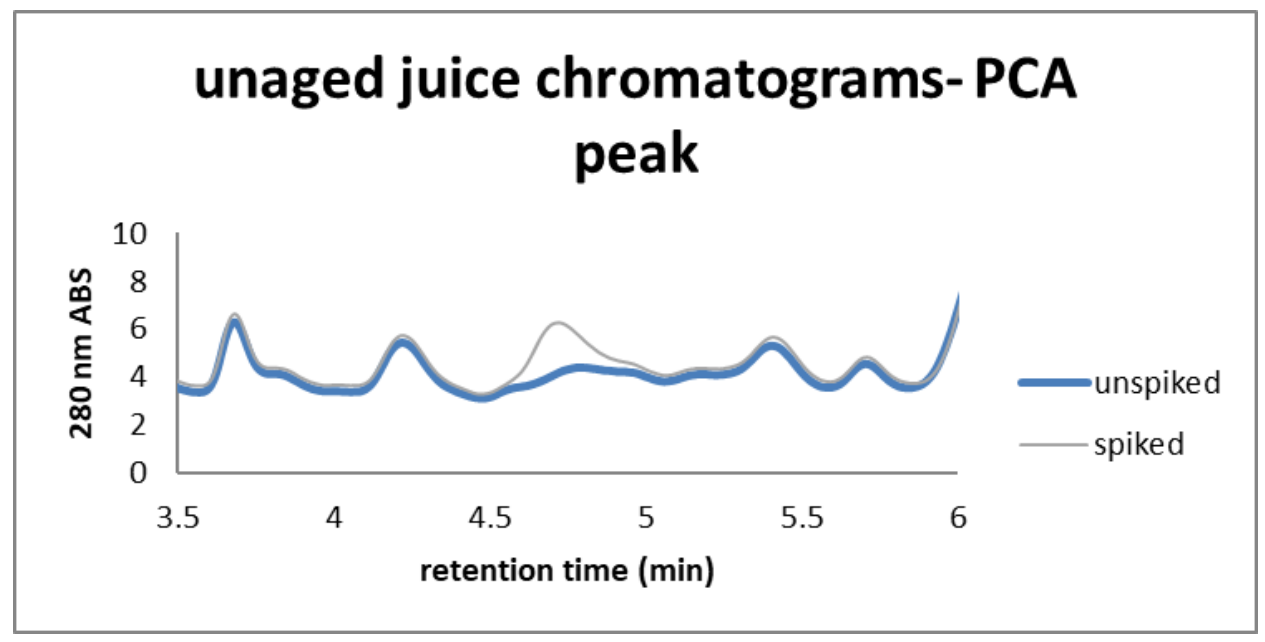

Figure S7. "Data not shown" in article provided by Dr. Dorris, demonstrating how they identified GA and PCA peaks in a 280 nm chromatogram by standard addition to the whole juice samples.

3. Using the concentration curves of PCA (y=0.2162x+0.0698) and GA (y=0.4214x+0.1436) at 280 nm and the peak area of GA (2.0918 and 2.5287) and PCA (0.2848 and 0.2374) in unaged and aged grape juice, respectively, calculate the concentrations of GA and PCA in unaged and aged grape juice in the measured samples. What conclusions can be made about how the polyphenol content changes in grape juice over time?

4. Figure 5C in the article shows the difference between an unaged and aged grape juice sample. Notice how many peaks there are in this real sample. What causes the additional chromatogram peaks? How will the complicated nature of this chromatogram affect your quantitative results? Do you believe the calculations you did in question 5 are accurate after seeing where the GA and PCA peak areas came from? What conclusions can you still draw about the polyphenol content in unaged vs aged grape juice?

5. After the HPLC lab, you'll get a chance to analyze your own sample! It's not too early to start brainstorming a question you want to investigate. This paper shows an interesting comparison between unaged and aged fruit juice polyphenol content. This website that Dr. Bolling recommended is a database of many different foods that

contain polyphenols. When you click on a food, it will show you the polyphenol content, how the polyphenol content was determined, and what papers/studies did the experiments. This is an amazing resource as you determine what questions you and your group want to explore! Take the chance to explore this website. Consider what questions you can ask, what information is out there, and how you may want to analyze your samples.

Instructor Guidance

- **Pre-lab Talk Notes**
    - Much of this lab is in your hands. Be prepared for me (the TA) to not know the exact answer to your question.
    - Make sure you know how to use the instruments (and process the data-which will be checked by your TA) before going into the lab. You don't want to break the HPLC for everyone else!
    - If you would like to use glassware that is not found in your drawer, ask the stockroom if they have options that work for you.
    - All HPLC/uHPLC samples should be filtered prior to running on the instrument.
- **General Expected Schedule**
    - Students will sign up for 30-minute time slots to use the HPLCs. 10-minute time slots will be available to get a tour and submit samples to the uHPLC. Students will not have access to sign up for the HPLCs until they have analyzed the sample data, and students cannot sign up for the uHPLC until they have analyzed their collected uHPLC data. A sample schedule is provided below in Table S5.

Table S6. Sample Schedule for CH Experiment. An "x" means task is completed on that lab day.

Task?	Day 1	Day 2	Day 3
Analysis of Sample Data	x		
(Making of) Key Solutions		x	x
Analysis on HPLC		x	
Analysis on uHPLC			x

- **Implementation Considerations & Sample Results**
    - Besides the student-provided materials, TAs were provided a pre-built pre-lab PowerPoint, representative sample data, and an overview of the common mistakes/modifications students could make. *All of these materials can be provided by request*.
    - Student groups of 3-4, maximizing 3 students first.
    - Standards will be run on both the HPLCs and the uHPLCs by course staff. For our instruments...
        - HPLC: 1, 1, 50, 100, and 200 mg/L gallic acid; 10, 100, 200, 500, and 1000 mg/L catechin
        - uHPLC: 10, 50, 100, 200, 300, and 500 mg/L gallic acid; 1, 10, 50, 100, 200, 500, and 1000 mg/L catechin

- In preparing the tea samples, there are many papers that outline the concentration of catechin and gallic acid in different teas.
- The standard for brewing a cup of tea is 3 minutes in boiling water. Students should dilute to a consistent value after that to make comparable data.[5,6]
- Catechin will fluoresce, while gallic acid will not.[7]
- Retention times of catechin and gallic acid will vary depending upon instrument set up. For our specific columns and conditions, the averages and standard deviations during instructor testing are provided in the table below (Table S6).
    - Note that catechin and gallic acid retention times overlap for HPLC but not for uHPLC. This is by design, but the isocratic separation (on the HPLC) could be optimized to improve the resolution of the separation.
    - The uHPLC instrument used in this class has the fluorescence detector hooked up in tandem with the absorbance detector. This means that analyte flows through the absorbance detector and then through the fluorescence detector prior to going to waste, hence the later retention time for the sample molecule for fluorescence.
    - The retention times for catechin and gallic acid are not specific enough to avoid misidentification. Common molecules found in tea, for example (see Figure S6) do have overlapping retention times with catechin and/or gallic acid.

Table S7. Example Retention Times for Polyphenols of Interest

	Catechin	Gallic Acid
HPLC – Absorbance (sec)	109.64±0.59	108.12±0.50
uHPLC – Absorbance(min)	3.64±0.10	2.7±0.04
uHPLC -Fluorescence (min)	4.25±0.02	N/A

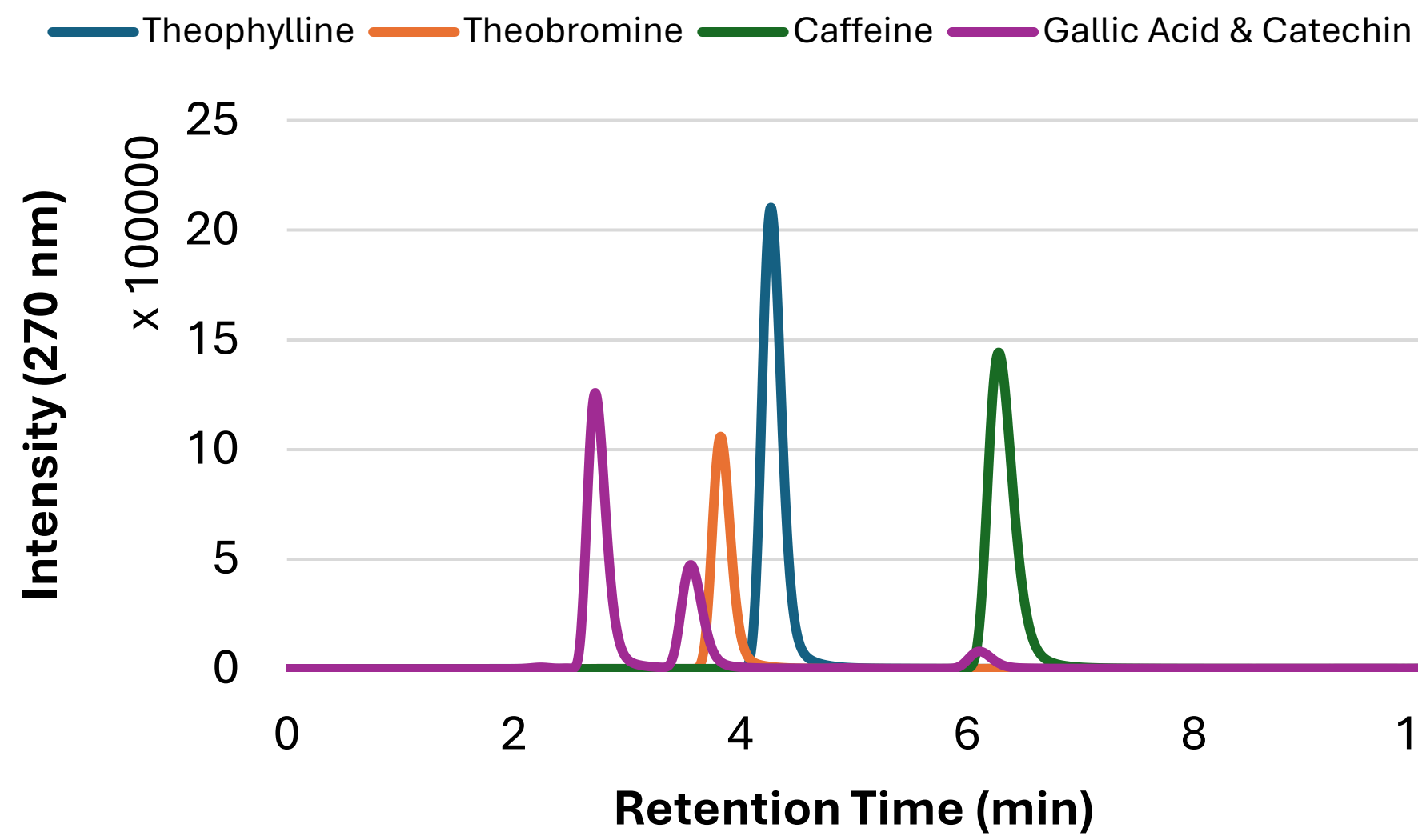

Figure S8. Representative chromatograms of caffeine, theobromine, theophylline, and a mixture of gallic acid (Peak 1, purple line) and catechin (Peak 2, purple line). Catechin elutes around a similar timeframe as theobromine, leading theobromine to be misidentified as catechin in the chromatography software. The gallic acid and catechin data was scaled 10x to match the peak sizes for the other 3 species.

- When quantifying the polyphenols of interest, due to the lack of specificity of the separation for polyphenols (with no secondary detection like mass spectrometry), we see that quantitation of the tea samples is very different when comparing absorbance vs. fluorescence data in uHPLC. Also, due to how close the peaks are (and lack of resolution), peak height and area can give varying quantities of the polyphenols in different teas. Students can test for these differences using a two-population t-test.

Table S8. Example Quantitation Amounts for Polyphenols of Interest in Black and Green Tea by uHPLC

	Catechin (mg/tea bag)	Gallic Acid (mg/tea bag)	Notes
Absorbance	151±46	122±35	Black Tea (Peak Height)
	639±49	245±36	Black Tea (Peak Area)
	114±38	92±36	Green Tea (Peak Height)
	244±75	177±49	Green Tea (Peak Area)
Fluorescence	0	N/A	Black Tea (Peak Height)
	0	N/A	Black Tea (Peak Area)
	0	N/A	Green Tea (Peak Height)
	31±10	N/A	Green Tea (Peak Area)

4. <u>Independent Sample Analysis (ISA)</u>

Instructor Guidance

- **Implementation Considerations & Sample Results**
  - Depending upon the goals of the lab series, students could stay in a previous group or be mixed up.
  - Students should compare 2-4 samples (to make the lab work manageable).
  - A good starting resource is http://phenol-explorer.eu/[8], which is a database that includes food stuffs, their polyphenol content, and associated citations.
  - Some example hypotheses include comparing:
    - Different brands of products (*i.e.,* dark chocolate, cocoa, hot chocolate, chocolate milk, witch hazel, extra virgin olive oil, ground cloves, grape juice, apple juice)
    - Differences in chocolate products (*i.e.,* Milk versus dark chocolate bars)
    - Different red wines (*i.e.,* Bordeaux versus Pinot Noir)
    - Store-bought versus dining hall fruit (*i.e.,* apples and applesauce)
    - Different juices (*i.e.,* cranberry versus apple, Naked Green Machine versus Blue Machine)
    - Different flavors of Bubbl'r (*i.e.,* pomegranate acai refresh'r versus cranberry grapefruit sprakl'r)
    - Different coconut water sources (*i.e.,* from fruit versus pre-packaged)
    - Wine versus grape juice (*i.e.,* alcoholic white versus white grape, non-alcoholic red versus red grape)
    - Different types of coffee (*i.e.,* light versus dark roast, caffeinated versus decaffeinated, ground versus instant)
    - Homemade versus store-bought fruit juice (*i.e.,* mango, apple)
    - Steeping time for English Breakfast Tea
    - Organic versus name brand (*i.e.,* apple juice, black tea)
    - Apple cider versus K-Cup apple cider
    - Different types of tea (*i.e.,* peach honey versus green tea)
    - Different types of fruits (*i.e.,* golden delicious versus pink lady apples)
  - Some products were just quantified (rather than compared) for polyphenol content, including:
    - Black walnut sap
    - Fruits (*i.e.,* blackberries)
    - Supplements (*i.e.,* grape seed extract, bilberry, Applewise/apple, ashwagandha root, grape)

        - Cocoa powder
        - Hemp powder
        - Fruit juice (*i.e.,* lemon, apple, plum)
        - Hot chocolate powder
        - Turmeric (NOTE: quantified curcumin, not gallic acid or catechin)
    - Example data sets are provided in Figures S9, S10, and S11.
    - Within this experience as is, the allowable, regulated deviation from the product label is not discussed directly with students or prompted as a topic of consideration. Adding this as a consideration in a statistical hypotheses could add a content-rich layer in a course, if appropriate.
    - Students may select samples that have no polyphenols present, and they would benefit from a reminder on the reporting of numbers below the detection limit (and how to statistically handle them).
        - If students "remove" points below the detection limit of a method, they will artificially skew their data towards higher values.
        - If students want to report numbers below the detection limit…
            - If no additional statistics are needed, reporting as "below detection limit" is appropriate.
            - If additional statistics are needed, reporting the value as "0" is appropriate but could skew data towards lower values.

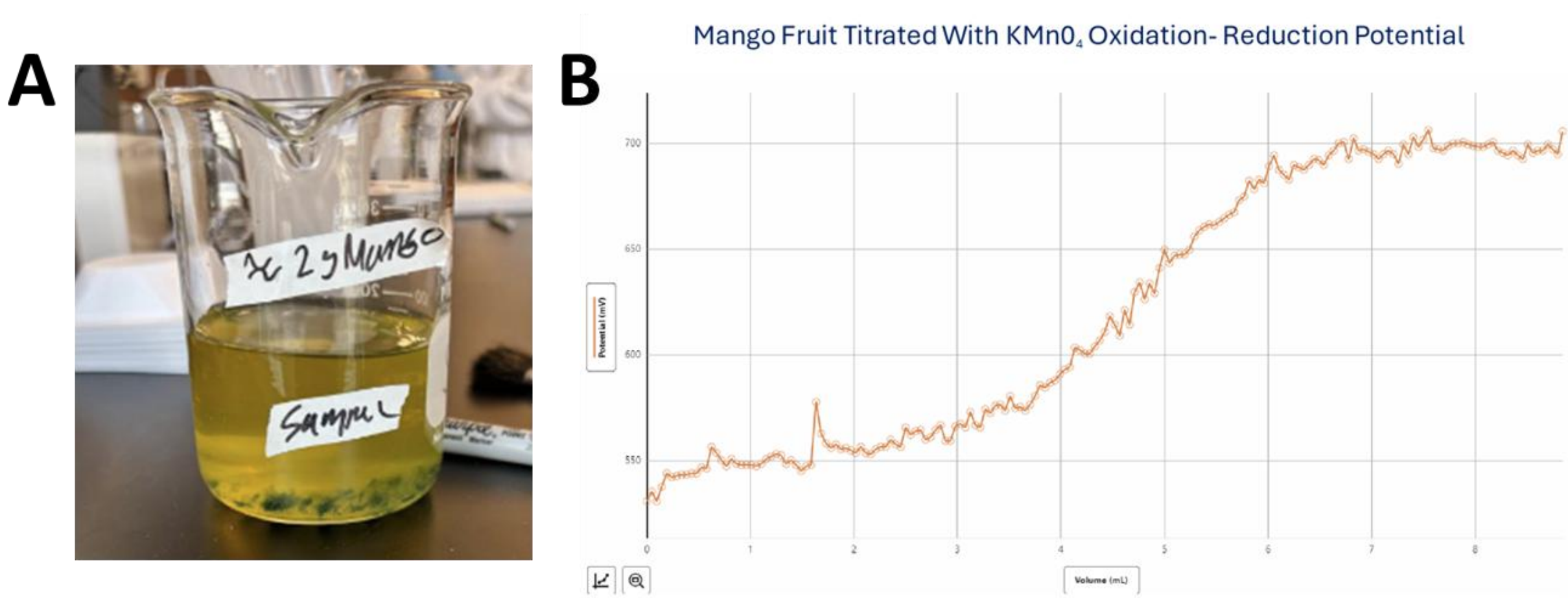

Figure S9. Screenshots of student final presentation results from analyzing mango fruit. (A) Photo showing the indigo carmine indicator interacting with the pulp of the mango. (B) LT curve collected from the analysis of mango fruit with the pulp removed via filtration.

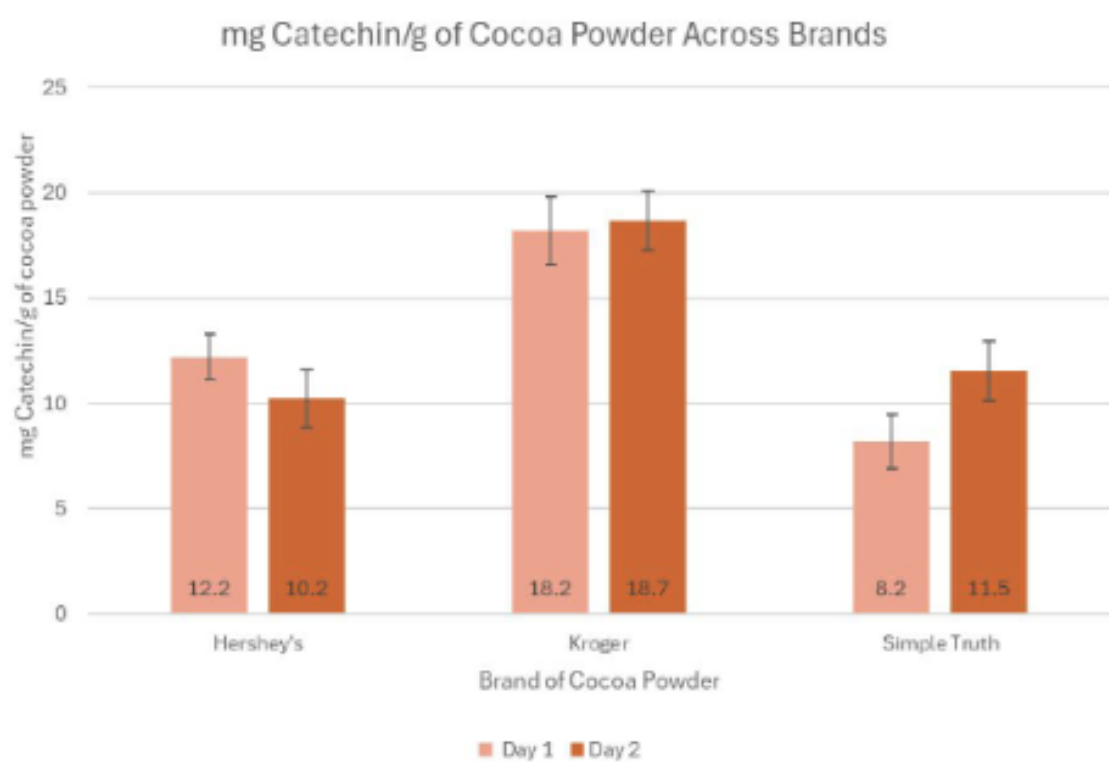

Figure S10. Screenshots of student final presentation results from analyzing cocoa powder by VS.

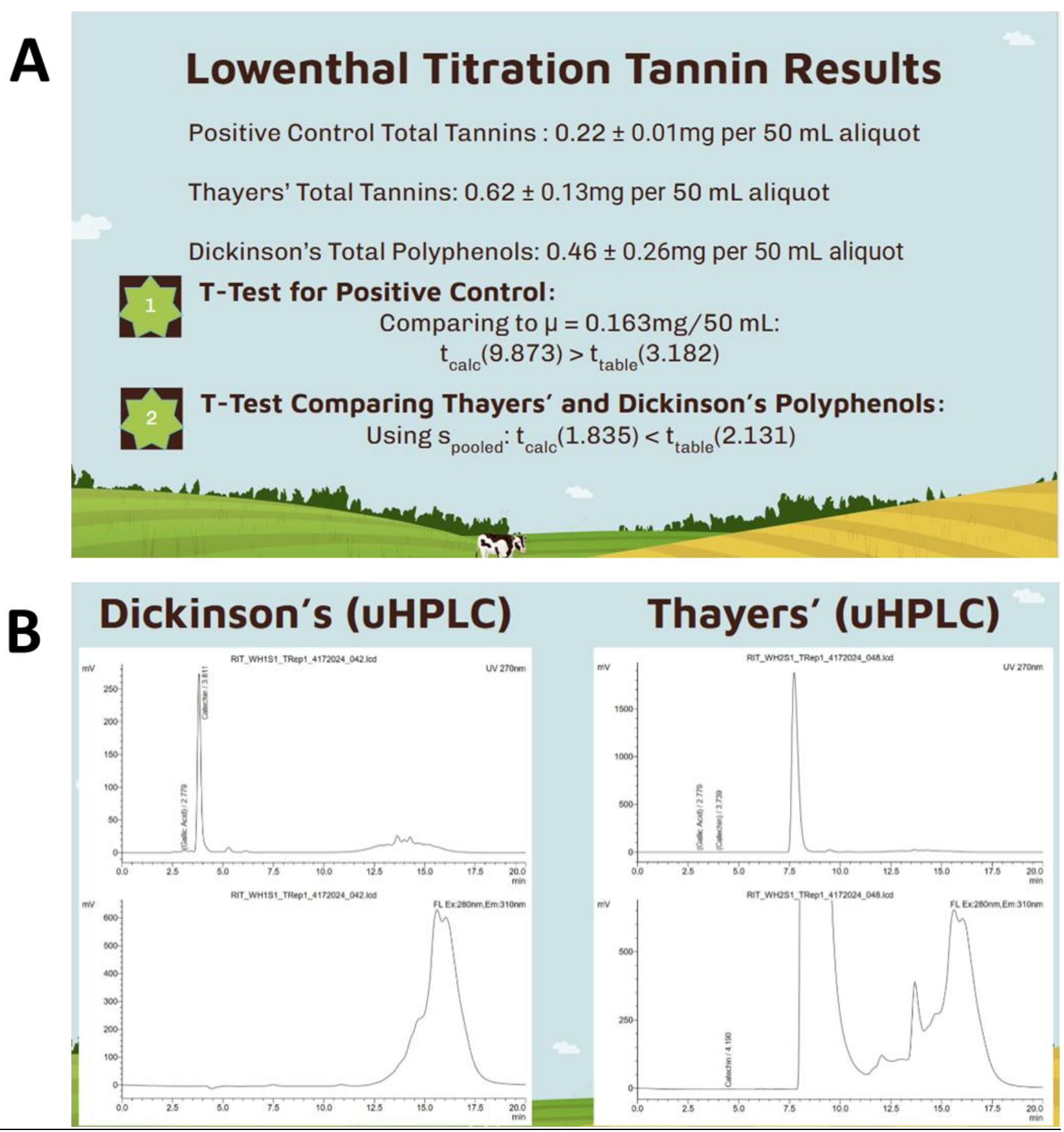

Figure S11. Screenshots of student final presentation results from different brands of witch hazel. In this case, the LT results (A) showed statistical differences between the amount of polyphenols measured, but CH results (B) showed that gallic acid nor catechin were the reason for those differences. Note that even though catechin was identified via UV detection for Dickinson's (top panel), the fluorescence detection (bottom panel) confirmed that this was not catechin, highlighting the importance of both detection methods.

5. Activities/Assessments Connection to Scientific Practices & Industry-Prioritized Skills

Table S9. Activities and Assessments and Their Intended Industry-Prioritized Skills[9] (complementary to Figure 2 in main text)

Activity/ Assessment	Communication	Problem Solving	Teamwork/ Collaboration	Critical Thinking	Technical Skills	Planning and Organization Skills	Personal Growth/ Development
Group Charter	■		■			■	
LT Pre-lab Preparation Worksheet				■			
LT Lab		■	■	■	■	■	
LT Lab Report & Abstract				■	■		
VS Pre-lab Preparation Worksheet				■			
VS Lab		■	■	■	■	■	
VS Lab Report & Abstract				■	■		
CH Pre-lab Preparation Worksheet				■			
CH Lab		■	■	■	■	■	
CH Lab Report & Abstract				■	■		
(Mid-semester& End-of-Semester) Peer Feedback							■
ISA Procedure (Preparation & Assessment)	■		■	■	■	■	
ISA Lab		■	■	■	■	■	
ISA Lab Notebook Pages					■		
ISA Group Meeting Presentation	■		■	■		■	
ISA Final Presentation (Preparation & Assessment)	■		■	■		■	

NOTE: Networking, Teaching/Training, Management, Organization Awareness, and Personal Value/Attributes are not included.

LEGEND: Colored in boxes are considered "marked".

Table S10. Activities and Assessments and Their Intended Scientific Practices[10] (complementary to Figure 2 in main text)

Activity /Assessment	Asking Questions	Developing & Using Models	Planning & Carrying Out Investigations	Analyzing & Interpreting Data	Using Mathematics & Computational Thinking	Constructing Explanations & Engaging in Argument with Evidence	Evaluating Information	Communicating Information
Group Charter								
LT Pre-lab Preparation Worksheet						■	■	
LT Lab			■			■		
LT Lab Report & Abstract				■	■	■		■
VS Pre-lab Preparation Worksheet						■	■	
VS Lab			■			■		
VS Lab Report & Abstract				■	■	■		■
CH Pre-lab Preparation Worksheet						■	■	
CH Lab			■			■		
CH Lab Report & Abstract				■	■	■		■
(Mid-semester& End-of-Semester) Peer Feedback								
ISA Procedure (Preparation & Assessment)	■		■					■
ISA Lab			■			■		
ISA Lab Notebook Pages				■	■	■		■
ISA Group Meeting Presentation				■		■	■	■
ISA Final Presentation (Preparation & Assessment)				■	■	■	■	■

LEGEND: Colored in boxes are considered "marked".

6. <u>Full Course Schedule for Spring 2024</u>

			LAB (MW)		LECTURE (MW)
**Week**	**Date**	**Day of Week**	**621/623**	**622/624**	**Topic**
1	22-Jan	Monday	***NO CLASS (Classes Begin Tuesday, January 23rd, 2023!)***		N/A
	24-Jan	Wednesday	Check in/Good Lab Practices		Course Introduction & Project Lab Introduction
2	29-Jan	Monday	Good Lab Practices		Units & Significant Figures
	31-Jan	Wednesday	Preparation of a Standard HCl Solution		Error Analysis
3	5-Feb	Monday	Determination of Acetylsalicylic Acid in Aspirin		Gaussian Curves & Confidence Intervals
	7-Feb	Wednesday	Quantifying Polyphenols by the Lowenthal Titration Method (Day 1)		Statistics
4	12-Feb	Monday	Quantifying Polyphenols by the Lowenthal Titration Method (Day 2)		Quality Assurance
	14-Feb	Wednesday	Quantifying Polyphenols by the Lowenthal Titration Method (Day 3)		Fundamentals of Spectrophotometry
5	19-Feb	Monday	*Study Day/Lab Make-up*		Beer's Law
	21-Feb	Wednesday	A Spectrophotometric Study of Fluorescein		Emission
6	26-Feb	Monday	Spectrophotometric Determination of Polyphenols by way of Vanillin Assay (Day 1)		*Super Homework #1 Work Time*
	28-Feb	Wednesday	Spectrophotometric Determination of Polyphenols by way of Vanillin Assay (Day 2)		Chromatography Theory (Part 1)
7	4-Mar	Monday	Spectrophotometric Determination of Polyphenols by way of Vanillin Assay (Day 3)		Chromatography Theory (Part 2)
	6-Mar	Wednesday	Introduction to Liquid Chromatography		Chromatography Applied
8	11-Mar	Monday	Using Liquid Chromatography and Polarity to Analyze Polyphenols (Day 1)		Equilibria Basics & Activity
	13-Mar	Wednesday	Using Liquid Chromatography and Polarity to Analyze Polyphenols (Day 2)		Solubility & Water Equilibria
9	18-Mar	Monday	Using Liquid Chromatography and Polarity to Analyze Polyphenols (Day 3)		Buffers
	20-Mar	Wednesday	Adventures with Buffer Solutions		Polyprotic Acids & Bases
--	25-Mar	Monday	***Spring Break***		
	27-Mar	Wednesday			
10	1-Apr	Monday	Study and Identification of an Unknown Weak Acid		Titration Curves
	3-Apr	Wednesday	Determination of an Indicator Constant: A Study of Bromocresol Green		Alpha Fractions
11	8-Apr	Monday	*Study Day/Lab Make-up*		Systematic Treatment of Equilibria (Part 1)
	10-Apr	Wednesday	Individual Sample Analysis (Day 1)		Systematic Treatment of Equilibria (Part 2)
12	15-Apr	Monday	Individual Sample Analysis (Day 2)		*Super Homework #2 Work Time*
	17-Apr	Wednesday	Individual Sample Analysis (Day 3)		Reduction-Oxidation Basics
13	22-Apr	Monday	Silver Electrode Study of Equilibria	Fluoride Ion Selective Electrode	Redox Standard Potentials
	24-Apr	Wednesday	Project Presentations Feedback Session		Nernst Equation
14	29-Apr	Monday	Fluoride Ion Selective Electrode	Silver Electrode Study of Equilibria	Mass Spectrometry Basics (Part 1)
	1-May	Wednesday	Project Presentations/Check Out		Mass Spectrometry Basics (Part 2)
Finals Week	8-May	Thursday	**Submit Super Homework #3 by End of Final Exam Period (May 9th, 2023 at 2:25 PM)**		

NOTE: Lab days associated with the lab experience (*i.e.*, graded for data collection and interpretation) are highlighted in green. Standard labs (*i.e.*, graded upon correctness) rounding out the semester are in white. The lab experience accounted for 26.4% of the final grade for the semester.

7. <u>Course Learning Objectives</u>

At the end of this course, students will KNOW or be able TO…

1. Perform chemical measurements, adapt analytical methods (*e.g.,* titrations, separations, electrochemical measurements, and spectroscopy), and interpret results for the purpose of quantitative analysis.
2. Evaluate laboratory data by applying statistical methods (*e.g.,* statistical uncertainty, confidence intervals, propagation of error).
3. Develop quantitative chemical models involving thermodynamics, equilibrium concentrations, separation performance and electrochemical potentials and compare them to experimental results.
4. Solve chemistry problems that connect concepts from across the curriculum and apply them to real world situations.
5. Design and execute independent analytical chemistry experiments using methods sourced from the scientific literature.
6. Effectively communicate experimental outcomes through written reports, oral presentations, and/or visual aids.

8. Grading Rubrics

Table S11. Final Abstract Grading Rubric

<table>
<tr><th>Criteria</th><th>Criteria Details</th></tr>
<tr><td>Background/Introduction/Hypothesis</td><td rowspan="5">• +3 (all good)
• +2 (minor issues)
• +1 (major issues)
• +0 did not cover</td></tr>
<tr><td>Methodology Explanation/Justification</td></tr>
<tr><td>Data/Results</td></tr>
<tr><td>Discussion/Conclusions/Future Directions</td></tr>
<tr><td>Structure/Grammar</td></tr>
</table>

This rubric was developed[11] and customized for the Polyphenol Pick Your Own Adventure Final Presentation. Circle the box for the evaluation you wish to give for this presentation and add any comments.

Criteria	Developing<br>*(Did not complete any required elements well)* (50%)	Novice<br>*(At least 1 item was done well)* (70%)	Intermediate<br>*(More than 1 thing (but not all) was done well)* (85%)	Sophisticated<br>*(Everything was done well)* (100%)
**Organization/Clarity/Flow**<br>• Length<br>• Order of and inclusion of required sections<br>• Transitions<br>• Clarity<br><br>*10 Points*	Presentation was inappropriate length (*i.e.,* long (>1 min over) or very short (<2 minute under)). No appropriate sections included. Transitions did not exist.	Presentation was almost inappropriate length. Missed most required sections. Presentation flow was lacking.	Presentation was/ was almost appropriate length. Most required sections were present. All ideas were logical and flowed.	Presentation was appropriate length. All required sections were present. All ideas were clearly logical and flowed throughout the presentation.
**Subject Knowledge**<br>• Includes relevant deliverables<br>• Depth and accuracy of materials<br>• Ability to answer questions<br><br>*20 Points*	Presentation contains multiple, factual errors and lacks any relevant answers to deliverables; no data presented. Unable to answer any questions.	Presentation contains major omissions (missing >1 deliverable) or mistakes that detract from key points. Some data is presented. Not able to clearly answer questions.	Almost all relevant information is included (missing 1 deliverable), and at an appropriate depth and accuracy. Students can usually answer questions.	Presentation contains accurate and complete information to tell an engaging story. Depth is appropriate. Students can answer question clearly and concisely.
**Presentation Support and Visual Aid Quality**<br>• References/citations<br>• Visual aids (graphs, tables)<br>• Text & grammar<br><br>*15 Points*	Little or no message support designed into the presentation; visual aids missing or inadequate. No citations. Major text or grammatical issues.	Some data supported using visual aids, but visual aids are hard to interpret or view. No citations. Minor to major text or grammatical issues.	Project results are mostly supported by visual aids. Most information is correctly referenced. Minor text or grammatical issues.	Project results are fully supported by visual aids; reported values and are correctly referenced. No text or grammar errors.
**Elocution & Collaboration**<br>• Pace & delivery<br>• Verbal & body language (energy/volume/gestures)<br>• Appearance & eye contact<br>• Group collaboration<br>*5 Point*	Delivery techniques (posture, gesture, eye contact, pace, and energy/volume) detract from the understandability of the presentation. Only 1 group member presented/was knowledgeable.	Delivery techniques (posture, gesture, eye contact, pace, and energy/volume) make the presentation understandable. Not all group members presented or seemed knowledgeable.	Delivery techniques (posture, gesture, eye contact, pace, and energy/volume) make the presentation interesting. Not all group members presented or seemed knowledgeable.	Delivery techniques (posture, gesture, eye contact, pace, and energy/volume) make the presentation compelling. All group members present as a team and seemed knowledgeable.

Expectations

Each item has a checkbox that you can mark if that item was done well. If you have a reason why that item was not checked, make sure to include it as a comment.

Organization/Clarity/Flow

- ☐ Length
  - 15-minute presentation
- ☐ Order of and inclusion of required sections
  - Background/introduction, methods, results, discussion, conclusion
- ☐ Transitions
  - Smooth and natural
- ☐ Clarity
  - Individual content sections are clear and well-supported

Subject Knowledge

- ☐ Includes relevant deliverables
  - Select a sample of interest (not tea) and analyze it using a Vanillin or Lowenthal (and justify that method using quantifiable evidence).
  - Develop an important (not just number confirming) hypothesis that you will statistical test with this sample (with at least 3 replicates per group member)
  - Describe how the quality assurance (at least 2) metrics support your experimental results.
  - Example calculations for all major findings.
- ☐ Depth and accuracy of materials
  - Includes appropriate data to support deliverables
- ☐ Ability to answer questions
  - Correctly and at appropriate level
  - All students in group can answer the question

Presentation Support and Visual Aid Quality

- ☐ References/citations
  - All data/information found from outside sources are cited
- ☐ Visual aids (graphs, tables)
  - Be able to summarize all major findings in figures (preferred, at least 2) or tables (if needed)
  - All required components (legend, title, axes labels w/ units, caption)
  - Appropriate colors & resolution
  - Relevance to point being made
- ☐ Text & grammar
  - No large blocks of text
  - Appropriate grammar

Elocution & Collaboration

- ☐ Pace & delivery
  - Not reading off slides
- ☐ Verbal & body language (energy/volume/gestures)
- ☐ Appearance & eye contact
- ☐ Group collaboration
  - All members should be equally contributing to presentation

9. Survey Questions[12]

Demographic Questions (Pre-semester Experience)

Q1 What is your major?

- ◯ Chemistry (1)
- ◯ Chemical Engineering (2)
- ◯ Biochemistry (3)
- ◯ Other: (4) ____

Q2 What year of your undergraduate career are you in?

- ◯ 1st (1)
- ◯ 2nd (2)
- ◯ 3rd (3)
- ◯ 4th (4)
- ◯ 5th or higher (5)

Q3 Please check off all previous chemistry courses you have completed

- ☐ Chem 103 (2)
- ☐ Chem 104 (3)
- ☐ Chem 109 (4)
- ☐ Chem 115 (5)
- ☐ Chem 116 (6)
- ☐ Chem 260 (7)
- ☐ Chem 311 (8)
- ☐ Chem 343 (9)
- ☐ Chem 344 (10)
- ☐ Chem 345 (11)
- ☐ Chem 346 (12)
- ☐ Tested out/transferred chemistry credit (Please list all): (1) ____
- ☐ Other chemistry courses taken at UW - Madison (Please list all) (13) ____

Q4 Are you currently involved in undergraduate research?

- ◯ Yes (1)
- ◯ No, but I have participated in undergraduate research previously (2)
- ◯ No, but I plan to in the future (3)
- ◯ No, and I don't plan to in the future (4)

Q9 Which of the following best describes you?

- ◯ Male (1)
- ◯ Female (2)
- ◯ Non-binary (3)
- ◯ Not indicated (please specify) (4) ____
- ◯ Prefer not to say (5)

Q10 Is English your first or primary language?

- ◯ Yes (1)
- ◯ No (2)
- ◯ Prefer not to say (3)

Q11 Please select the highest level of education attained by a parent or guardian

○ Less than high school (1)

○ High school graduate (2)

○ Some college (3)

○ 2 year degree (4)

○ 4 year degree (5)

○ Master's or Professional degree (6)

○ Doctorate (7)

○ Prefer not to say (8)

Q12 Does at least one parent or guardian have a degree/work in STEM?

○ Yes (1)

○ No (2)

○ Prefer not to say (3)

<u>Skill Gains Questions (Pre-semester Experience)</u>

Q1 In this section, you will be asked about possible skill benefits you may have gained from this project lab experience. Please rate the following statements:

	No gain or very small gain (1)	Small gain (2)	Moderate gain (3)	Large gain (4)	Very large gain (5)
Ability to read and understand primary literature (1)	○	○	○	○	○
Ability to integrate theory and practice (2)	○	○	○	○	○
Learning laboratory techniques (3)	○	○	○	○	○
Ability to design experiments (4)	○	○	○	○	○
Ability to analyze data and other information (5)	○	○	○	○	○
Skill in interpretation of results (6)	○	○	○	○	○
Skill in science writing (7)	○	○	○	○	○
Skill in how to give an effective oral presentation (8)	○	○	○	○	○
Understanding of how scientists think (9)	○	○	○	○	○

Q6 Why has your career interest changed or not changed due to the project lab?

10. References

(1) Ma, S.; Kim, C.; Neilson, A. P.; Griffin, L. E.; Peck, G. M.; O'Keefe, S. F.; Stewart, A. C. Comparison of Common Analytical Methods for the Quantification of Total Polyphenols and Flavanols in Fruit Juices and Ciders. *J. Food Sci.* **2019**, *84* (8), 2147–2158. https://doi.org/10.1111/1750-3841.14713.

(2) Sequeira, C. A.; de Souza Júnior, R. S.; Borges, E. M. A STEAM-Based Laboratory Approach: Evaluating Vitamin C Content with Multiple Titration and Statistical Methods. *J. Chem. Educ.* **2025**, *102* (7), 3018–3026. https://doi.org/10.1021/ACS.JCHEMED.4C01414.

(3) Bolling, B. W.; Chen, Y. Y.; Kamil, A. G.; Chen, C. Y. O. Assay Dilution Factors Confound Measures of Total Antioxidant Capacity in Polyphenol-Rich Juices. *J. Food Sci.* **2012**, *77* (2), H69. https://doi.org/10.1111/J.1750-3841.2011.02538.X.

(4) Dorris, M. R.; Voss, D. M.; Bollom, M. A.; Krawiec-Thayer, M. P.; Bolling, B. W. Browning Index of Anthocyanin-Rich Fruit Juice Depends on PH and Anthocyanin Loss More Than the Gain of Soluble Polymeric Pigments. *J. Food Sci.* **2018**, *83* (4), 911–921. https://doi.org/10.1111/1750-3841.14106.

(5) Henning, S. M.; Fajardo-Lira, C.; Lee, H. W.; Youssefian, A. A.; Go, V. L. W.; Heber, D. Catechin Content of 18 Teas and a Green Tea Extract Supplement Correlates with the Antioxidant Capacity. *Nutr. Cancer* **2003**, *45* (2), 226–235. https://doi.org/10.1207/S15327914NC4502_13.

(6) Luaibi, H. M.; Alfarhani, B. F.; Hammza, R. A. Comparative Assessment Of Catechin And Gallic Acid Content In Different Brands Of Black And Green Tea. https://doi.org/10.1088/1742-6596/1294/5/052056.

(7) Du, C.; Ma, C.; Gu, J.; Li, L.; Zhu, C.; Chen, L.; Wang, T.; Chen, G. Rapid Determination of Catechin Content in Black Tea by Fluorescence Spectroscopy. *Journal of Spectroscopy* **2020**, *2020* (1), 2479612. https://doi.org/10.1155/2020/2479612.

(8) *Database on Polyphenol Content in Foods - Phenol-Explorer*. http://phenol-explorer.eu/ (accessed 2026-04-18).

(9) Hamilton, D.; Castillo, A.; Atkinson, M. B. Survey of Instrumentation Use in Industry: What Does Industry Want New Chemists to Know? *J. Chem. Educ.* **2024**, *101* (5), 1883–1890. https://doi.org/10.1021/ACS.JCHEMED.3C00990.

(10) Council, N. R. *A Framework for K-12 Science Education: Practices, Crosscutting Concepts, and Core Ideas*; The National Academies Press: Washington, DC, 2012. https://doi.org/10.17226/13165.

(11) *VALUE Rubrics - Oral Communication | AAC&U*. https://www.aacu.org/value/rubrics/value-rubrics-oral-communication (accessed 2026-04-18).

(12) Lopatto, D. Survey of Undergraduate Research Experiences (SURE): First Findings. *Cell Biology Education*. December 2004, pp 270–277. https://doi.org/10.1187/cbe.04-07-0045.
