# Supplementary material for: Pick Your Own Adventure: Orthogonal Methods for Analyzing Polyphenols
Source: J Chem Educ. 2026 Jul 17;103(8):4243–54. doi: 10.1021/acs.jchemed.5c01459 (PMC13480120; doi:10.1021/acs.jchemed.5c01459)
Supplement: Supplementary file 4 [file ed5c01459_si_005.pdf]

Pick Your Own Adventure: Orthogonal Methods for Analyzing Polyphenols

Amanda Rae Buchberger[1]*, Kristel M. Forlano[1], Madeline J. Lund[1], Pamela S. Doolittle[1], Dominic M. Colosi[1], Thao U. Duong[1], Brian J. Esselman[1], Kyoung-Shin Choi[1], Robert J. Hamers[1]

[1]Department of Chemistry, University of Wisconsin-Madison, 1101 University Avenue, Madison, WI 53706

*Email Addresses:
amanda.buchberger@wisc.edu

Lab Manual for Independent Student Analysis (ISA) Lab Module

PICK YOUR OWN ADVENTURE: PERSONAL POLYPHENOL SAMPLE ANALYSIS

Using what students have learned thus far about analyzing polyphenols, student groups will select a sample of their own interest to analyze for polyphenol content. Working in groups, students will develop a hypothesis around a sample, adapt methods (and justify that method choice) to test the hypothesis, analyze data, and report the results.

BACKGROUND

Analytical chemists spend their time thinking about what exactly they wish to measure, what methods to use to make that chemical measurement, and then how to frame the results with some measure of accuracy and precision. **Our goal for this lab series is to quantify polyphenols.**

You have gained experience using three methods for analyzing polyphenols. Each possesses positive and negative qualities, depending on the sample of interest. In this experiment, as a group, you will decide a sample of interest that will be analyzed by either (or both!) the (a) Lowenthal titration[1] or (b) the Vanillin spectrophotometry method[2]. Then, you will have the choice (if you wish) to submit a sample for chromatographic analysis[3] to provide complementary/orthogonal information to your other analyses.

> ***Did You Know?***
>
> "We work in basically any part of the food industry, from infant formula, standard food, pet foods, a lot in dietary supplements."
> *-Dan Berg*
>
> All of those samples will have a different preparation and analysis process. Industry needs to figure out what will work best, and so will you!

Your group will be responsible for all aspects of the experimental design from preparing the sample for analysis, adapting a method for this purpose, collecting data, analyzing results, and reporting an answer framed with some statistical significance. Remember that you have already done the leg work of the method development (figured out the concentrations, linear ranges, colorimetric endpoints, *etc.*), so much of your focus will be specific to your sample, not the method. If you Google "Polyphenol Rich Samples", you can discover >14 million hits, meaning that there are tons of different options for a sample; be inspired and unique!

Upon completion of this project, plan to accomplish the following deliverables:

1. Recognize and compare/contrast authentic, modern analytical methods used to analyze polyphenols.
2. Construct and justify a method to analyze and statistically test a hypothesis focused on sample of personal interest.
3. Analyze and validate results with appropriate statistical methods and quality assurance and control metrics, including detection limits, controls, and the percent recovery of a spike.
4. Summarize and communicate findings in an oral presentation and written abstract.

PRELABORATORY EXERCISES

Below is a list and short description of all pre-lab assessments you will complete. More details and expectations are posted on Canvas.

1. **Submit Peer Feedback for your initial groups.** In the groups you've worked with the previous weeks, share commendations and constructive comments for improvement. Your feedback will help those members to be better members of your teams. Be honest (but constructive). Be thoughtful. Be kind.
2. **Develop a draft procedure**. Using your past experiences in analyzing polyphenols, put together a plan for how to analyze your sample of interest and interface it with your method(s) of choice. See Experimental section for details that should be considered when making your procedure comprehensive.

Pro Tip

Talk to the stockroom if you want to use other sized glassware/equipment than what is in your drawer! Examples of what he has includes:

- Volumetric Flasks (10, 25, 50 mL)
- Volumetric Pipets (0.5 mL; 0.1-1.0 mL)
- Wiretrols (5/10, 25, 50/100 uL)
- Graduated Cylinders (10, 50, 100 mL)
- Morter and Pestle
- Spice Grinder
- Vortex Mixer
- Sonic Cleaner
- Centrifuge
- Furnace *(to ash samples)*
- pH Paper & Probes
- Parafilm
- Aluminum Foil
- Plastic Wrap
- Vacuum Filtration Setups (250, 1000 mL)
- Freezer/Fridge
- Bottles (125, 250, 500, 1000 mL)

BEFORE YOU TAKE THE QUIZ

There is no pre-lab quiz for this experiment. The point of the pre-lab quiz is to make sure you are fully prepared for the experiment and understand the calculations. In this case, we are expecting that you already have a good grasp without us checking!

EXPERIMENTAL

Proposed Timeline/Plan

Day 1	Day 2	Day 3
Up to you!	Up to you!	Up to you!

Key Considerations

1. First thing, you will want to pick a sample. Below are some guiding questions for selecting a sample. NOTE: Pick anything OTHER than a tea product (since we have analyzed a green tea tablet, black tea, and green tea). A good place to start looking is http://phenol-explorer.eu/. *(If you have a compelling reason/hypothesis for analyzing a tea sample, you must get approval from course staff prior to selecting that as your sample).*
    a. What is the importance of this sample? Is there a story you can tell to make others care about the analysis of this sample? **Your goal should be more than just to "see if the packaging is correct".** Thing about the broader, societal impacts of the sample and this analysis.
        i. *Example: Tea is the #1 consumed beverage in the world. And, tea contains a lot of polyphenols, which are effective antioxidants. We want to quantify these and compare them across X teas to see which tea is better for your health.*
    b. How will you obtain this sample? How much do you (estimate) you will need?
    c. How do you plan to prepare it so that it (or at least the polyphenols) are in solution? Have a few options to try. If you need to do something different than just "dissolving" it, will the pH of the solution be a problem? What about other ions/molecules? Looking through the literature may help!
    d. What kind(s) of polyphenol(s) are (predicted or known to be) present? And does that impact what method works best for the sample?
2. Next, you will want to pick a method.
    a. Of the methods you have used, is one more or less appropriate for your sample? Why? What makes that method the best choice? Or is there a different method that is better? Justify your choice. **A justification should be more than just "I like this method better".** It should be quantifiable and convince and audience that your method is the obvious choice.
    b. Do you need to modify the chosen method at all for your new sample? Do you need to do some method development again?
3. You must report your quantified amount with some statistical information. Put together at least one hypothesis to test about your sample of interest.
    a. Capture an adequate number of replicates to perform statistics. The number of replicates expected will vary depending upon which method you choose. *Talk to the course staff if you are unsure about how many replicates you are expected to collect.*
        i. You should plan to acquire AT LEAST 3 replicates *(remember the difference between technical and biological replicates!)* per person.

    b. Your hypothesis should have importance connected to point #1 above. You should avoid just confirming what is on the bottle unless you have compelling, real-world reasoning. Examples include:
        i. Are there multiple or different versions of the sample that you can compare?
        ii. Is there a documented value you can compare against (that has important significance)?
        iii. Is there information on the internet that you want to confirm (or prove wrong)?
4. You will want to make sure you consider other quality assurance metrics.
    a. Should you do a positive control? An MDL analysis? A spike recovery? Is negative control possible or useful? *(The answer is "yes" to most of these questions!)*
        i. The expectation is that you perform AT LEAST 2 quality assurance metrics, no matter the method you select.
    b. **Analyzing the sample via chromatography is NOT considered a quality assurance metric.**

RESULTS/CALCULATIONS

Below is a list and short description of all post-lab assessments you will complete. More details and expectations are posted on Canvas.

1. **Abstract about your research project.** This will be similar to what you did previously in the 3 standard labs. You are welcome to send course staff a draft to review for feedback, but your assessment will be on the FINAL (graded for appropriateness and professionalism) version. You should capture all the project deliverables in this abstract.
2. **Presentation about your research project.** You will do a shorter practice round (Group Meeting Presentation) in discussion as well as give a final presentation to a panel of course staff during the designated lab period. You should capture all the project deliverables and key considerations in this final presentation, and the practice round will have some provided focus.
    a. Make sure you address the chemistry and justification in your background/methods.
    b. Your major data/methods should be captured in figures or tables, such as calibration curves, comparison graphs, or methodology workflow. All graphs should include a title, (axes) labels, and units/numbers as appropriate.
        i. It is expected that you will have <u>at least</u> 2 data figures in your presentation.
        ii. Be careful NOT to put too much data in tables. If you do include data as tables, make sure to consider units and appropriate significant figures.
3. **Lab notebook pages.** This will capture all your <u>individual</u> notes about the project lab.
4. **Peer evaluation.** You will be assessed by your classmates in your final project groups and their perception of your participation in the project lab.

CHALLENGE QUESTIONS FOR YOUR LAB NOTEBOOK

Challenge questions are designed to make you think deeper about the concepts you learned in this lab. There may be multiple answers to these questions! Any honest effort at answering the questions will be rewarded.

1. Project labs are challenging! Course staff will ask challenging questions during your presentation. These questions may center around the sample you chose and details about its preparation; address clarification of calculations or key findings; or inquire what direction you might take the experiment if you had more time. Keeping a good record of your experimental details in the laboratory notebook may prove helpful in reminding you about what you did over the course of the project. In your final lab notebook pages, predict what sort of questions the course staff may ask to prepare for the presentation!

REFERENCES

1. "The Lowenthal Titration Method to Quantify Polyphenols". *A Manual of Experiments for Analytical Chemistry.* Department of Chemistry at UW- Madison, Spring 2024.
2. "Spectrophotometric Quantitation of Polyphenols Leveraging Vanillin Chemistry." *A Manual of Experiments for Analytical Chemistry.* Department of Chemistry at UW- Madison, Spring 2024.
3. "The Two Phases of Separation: Using Liquid Chromatography and Polarity to Analyze Polyphenols". *A Manual of Experiments for Analytical Chemistry.* Department of Chemistry at UW- Madison, Spring 2024.

Final Words of Wisdom from Dan Berg

"I suggest doing extra things to do research and investigate things you don't know" - *Dan Berg*

Look for internships and get practical experience. Find product development projects and student competitions. Learning how to dig into problems understand them, and communicate you understand the problem and how to test it is vitally important for careers in food science.

Can you use this project lab experience in a job interview? Think about all you've learned and the skills you've developed.

Thank you, Dan, for providing insights into the industrial aspect of the food sciences field through this semester!

Here's an example of some of Dan's work with Eurofins: https://www.eurofinsus.com/food-testing/resources/nutrient-analysis-of-dietary-supplements-white-paper-and-faq/
