# Supplementary material for: Pick Your Own Adventure: Orthogonal Methods for Analyzing Polyphenols
Source: J Chem Educ. 2026 Jul 17;103(8):4243–54. doi: 10.1021/acs.jchemed.5c01459 (PMC13480120; doi:10.1021/acs.jchemed.5c01459)
Supplement: Supplementary file 6 [file ed5c01459_si_009.pdf]

Pick Your Own Adventure: Orthogonal Methods for Analyzing Polyphenols

Amanda Rae Buchberger[1]*, Kristel M. Forlano[1], Madeline J. Lund[1], Pamela S. Doolittle[1], Dominic M. Colosi[1], Thao U. Duong[1], Brian J. Esselman[1], Kyoung-Shin Choi[1], Robert J. Hamers[1]

[1]Department of Chemistry, University of Wisconsin-Madison, 1101 University Avenue, Madison, WI 53706

*Email Addresses:
amanda.buchberger@wisc.edu

Lab Manual for Titration Analysis of Polyphenols using Lowenthal Titration (LT) Lab Module

THE LOWENTHAL TITRATION METHOD TO QUANTIFY POLYPHENOLS

In this lab, students will learn about one option for quantifying polyphenols called the Lowenthal redox titration. Students will visualize the colorimetric endpoint and connect it with an equivalence point to then quantify standard solutions of the common polyphenols, catechin and gallic acid.

Visual Abstract:

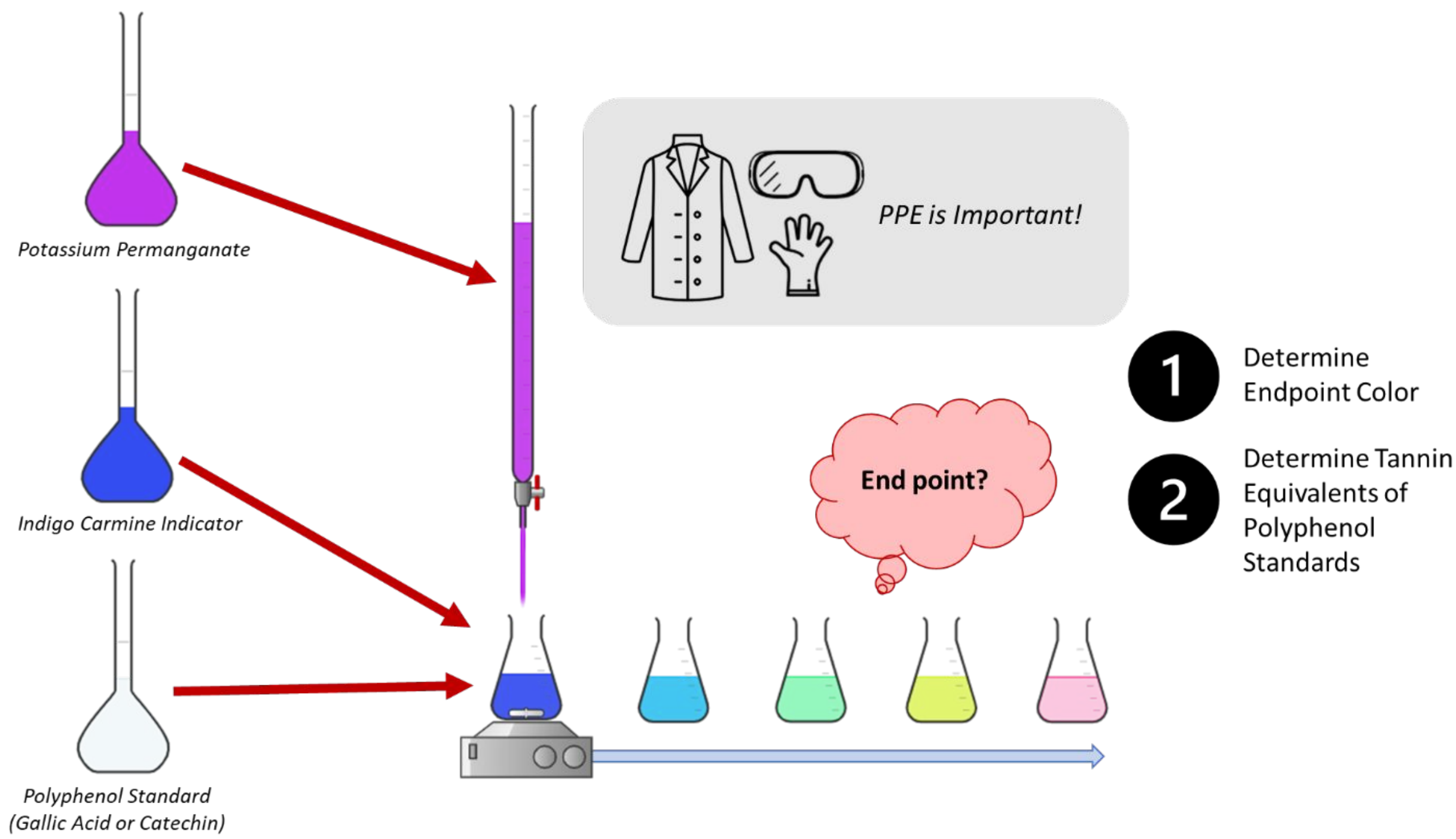

*To cite this lab manual: "*The Lowenthal Titration Method to Quantify Polyphenols*"*. A Manual of Experiments for Analytical Chemistry. *Department of Chemistry at UW- Madison, Spring 2024.*

Did You Know?

Keep an eye out for these green boxes throughout your lab manuals! They will contain little tidbits connecting the experiments you're completing with real research and industrial applications, provided by Dr. Bradley Bolling in the Department of Food Science at UW-Madison and Dan Berg, an Analytical Services Manager at Eurofins Food Testing Chemistry in here in Madison!

BACKGROUND

Just like there are many tools in a toolbox, the analytical chemist has a plethora of chemistries, techniques, and instrumentation to identify and quantify analytes of interest. Qualitatively, we must first discern WHAT needs to be measured and, knowing that, can select and characterize an appropriate method for analysis. **In this lab series, we will explore a variety of techniques used to quantify polyphenols.** This manual focuses on a titration-based method for quantifying polyphenols.

<u>Importance</u>

Generally, polyphenols are a large family of molecules (**>8,000 identified**) that (as you guessed) contain many phenol (C_6H_5-OH) groups.[1] They are incredibly diverse, ranging in size from ~100 to ~800 Daltons (~g/mol). There are different classes (and structural motifs). Generally, non-flavonoids contain only one phenol ring, and flavonoids contain two phenol rings. Polyphenols are naturally found in many fruits, vegetables, and teas. Interestingly, the pulp of fruits/vegetables is rich in non-flavonoid compounds, while the skins, seeds, and stems are rich in flavonoid compounds. Green tea has gained a lot of attention for its high concentrations of the polyphenol epigallocatechin gallate (EGCG).[2] Easily purchasable and simple polyphenols that you will work with in this lab are catechin (a flavanol) and gallic acid (a non-flavonoid, phenolic acid). The great variety in structures makes analyzing these molecules difficult without customizing or restricting the exact samples analyzed.

On a more microscopic scale, polyphenols are known primarily as antioxidants, meaning they inhibit oxidation. Uncontrolled oxidation inside the human body (*e.g.,* of DNA, proteins) results in cellular damage that can lead to downstream problems. Antioxidants react with these oxidizing species (*e.g.,* oxygen) to reduce their oxidative behavior and therefore reduce cellular oxidation in the body. Many people associate consuming antioxidants with reducing the effects of aging and mitigating risks of certain cancers and other diseases. This reduction/oxidation property of these molecules is something that many individuals wish to take advantage of to quantitatively characterize or quantify these properties.

<u>Did You Know?</u>

Polyphenols cannot be specifically labeled on food packaging according to the FDA. However, they can be labeled on dietary supplements.

<u>Building a Method (The Important Stuff)</u>

In developing a method to analyze polyphenols, we look to the literature to see how others have analyzed polyphenols. That way, we don't have to start from scratch! One of the oldest methods for analyzing polyphenols is a colorimetric titration from the 1800s called the Lowenthal permanganate titration. Titrations are a method that allow you to determine the amount of something (*e.g.,* moles) found in a sample of interest (called an "unknown") by comparing how much (*e.g.,* moles) of a known concentration solution is added. Depending upon the mole-to-mole ratio at the desired endpoint of the titration (usually a color change), we can determine how much of a molecule is present in that unknown sample. For polyphenols analysis, many papers and online resources outline the Lowenthal titration as the "classical" method, especially for apple and cider analysis.[3] Overall, the ***<u>chemistry</u>*** seems straightforward, but it is still a bit unknown and filled with assumptions. Firstly, the titration is based on

oxidation-reduction chemistry (shock!). In an acidic medium, MnO_4^- works as an oxidation agent of polyphenol species. The method was specifically developed to quantify tannins, a class of non-flavonoid polyphenols. Moving forward, we will assume the polyphenols of interest react in the same way tannins would with MnO_4^-. Now, what is the exact reaction we are working with? According to Appendix H in our textbook[4], in acidic conditions, permanganate can react in 2 different ways:

$$\boldsymbol{MnO_4^- + 4H^+ + 3e^- \leftrightharpoons MnO_2(s) + 2H_2O \qquad E^o = 1.692} \qquad (1)$$
$$\boldsymbol{MnO_4^- + 8H^+ + 5e^- \leftrightharpoons Mn^{2+} + 4H_2O \qquad E^o = 1.507} \qquad (2)$$

You can see that both reactions (under standard conditions) are both very product favored (based upon the high and positive E° values), meaning that it likes to accept electrons and become reduced. Which reaction is happening in this experiment? Ideally, only one reaction would be happening to keep the assay quantitative. Thus, we will want to make the solution as acidic as possible to favor the reaction described in Equation (2). We can also confirm that this is the case by watching for any solid formation during the experiment. If solid formation occurs, it means that either a mix of these reactions is happening OR only reaction (1) is happening. If that is the case, the accuracy of the method would be under question, as we cannot guarantee the mole-to-mole ratio is consistent during the entire reaction and thus cannot convert any of our data into a useful number.

Since polyphenols are thought to inhibit oxidation, a reasonable conclusion is they are oxidized themselves and thus release electrons. Thus, MnO_4^- and polyphenols would be a perfect match (since polyphenols want to give up their electrons while MnO_4^- wants to accept them). However, generally, the potentials of individual polyphenols are not well characterized. In fact, many studies focus on quantifying these values under biologically-relevant conditions (37 °C) rather than room temperature.[5] The standard reduction potentials determine at 37 °C, while still positive, are much less than MnO_4^- noted above (0.281 V vs. the standard hydrogen electrode (S.H.E.) for catechin), so, we can have some confidence that the reaction will work since the species with less positive E° will become oxidized.

Generally, catechin and gallic acid, two polyphenols used in this experiment, are believed to have a 2-electron transfer (see **Figure 1**). Based upon this information, we would assume the following net reaction (PP=polyphenol (gallic acid or catechin), assumed to start in its reduced form):

$$\boldsymbol{2.5PP(Reduced) + 8H^+ + MnO_4^- \rightleftharpoons 2.5PP(Oxidized) + Mn^{2+} + 4H_2O} \qquad (3)$$

Looking at the structures (**Figure 2**), though, it seems possible that other sites could change the number of electrons transferred. Thus, it is worth confirming this mole-to-mole ratio experimentally.

(a)

(b)

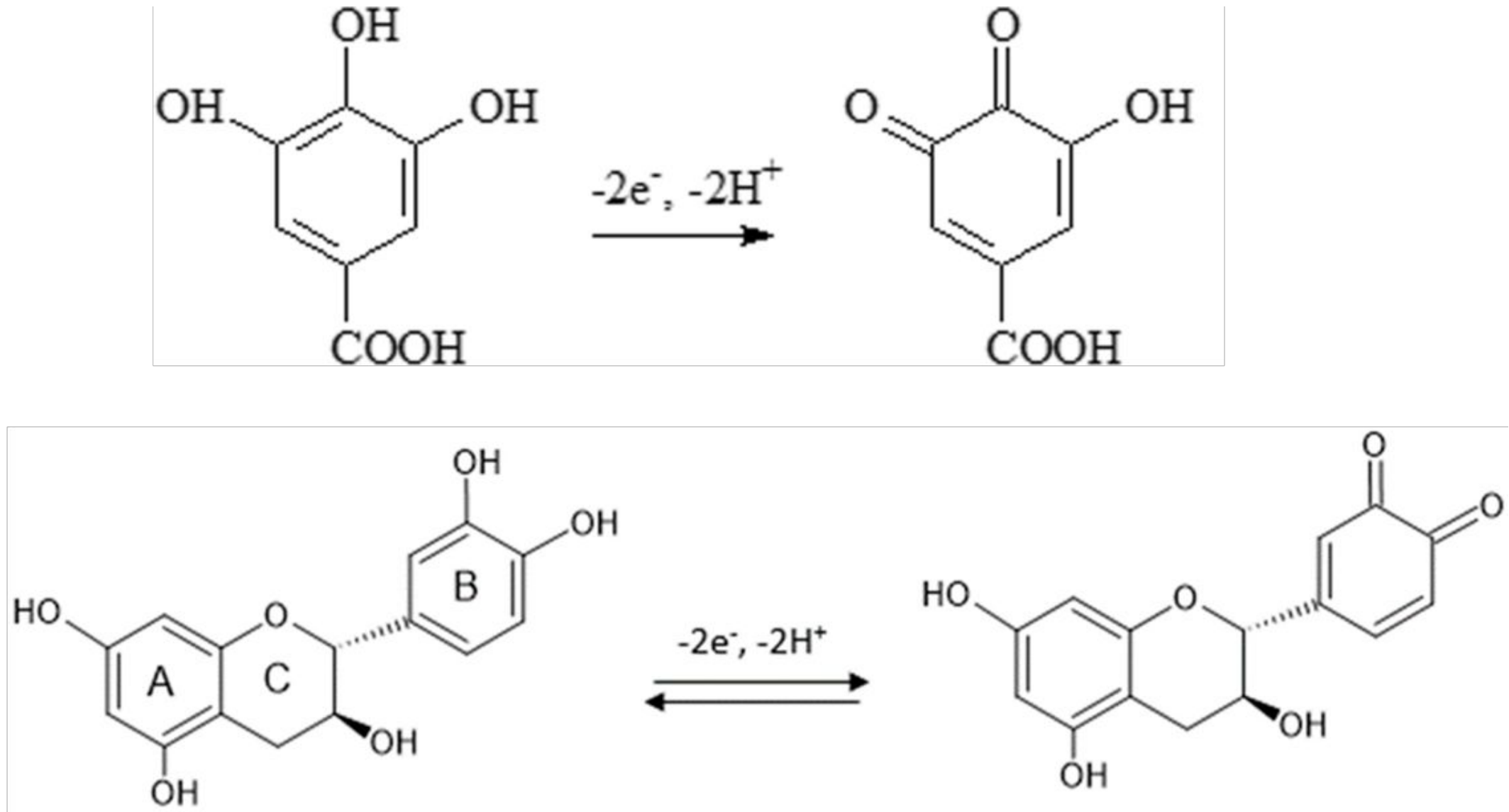

Figure 1: *Redox reactions of (a) gallic acid and (b) catechin.*

Ok…. time to use this method! The original paper was originally written in German, so, for ease, we will look at **another paper**.[6] When reading the paper, it is good to start with the methods to make sure they are even feasible in our lab spaces. On Page 522, it shows their methodology (**Figure 2**). The method describes a sample (juice) being mixed with an indicator (indigo carmine) and diluted to an arbitrary volume to make the titration easier. Then, potassium permanganate was added until a "yellow color was observed". Indigo carmine by itself also reacts with potassium permanganate. Thus, a "blank" titration was done, meaning just indicator was diluted and analyzed. This seems feasible in our lab spaces, as we have plenty of experience doing titrations!

First thing to notice is that the method looks at "percent tannin", meaning we are working to relatively quantify the amount of tannic acid equivalents in the sample. When doing relative comparisons, it is important to compare data the was collected/processed in the exact same way or normalized to some value. That means that you should not compare diluted juice to regular juice, for example.

Percent tannin (expressed as tannic acid equivalents) was measured using the Lowenthal permanganate titration method (Burroughs and Whiting, 1960; Lea, 2008; Lowenthal, 1877). For each juice sample, 1 mL juice was added to 150 mL distilled water, then 5 mL indigo carmine solution [1 g indigo carmine, 1 L distilled water, and 50 mL concentrated sulfuric acid (H_2SO_4)] was added. The mixture was titrated with a solution of 0.005 M potassium permanganate ($KMnO_4$) until yellow color was observed, and was comparable in color to the indicator blank. The indicator blank consisted of 5 mL indigo carmine solution mixed with 150 mL distilled water, and titrated with potassium permanganate until yellow color was observed. The percent tannins for each sample was calculated using the equation: total tannin (percent) = (X – Y)/10, where "X" is the amount of potassium permanganate added to the sample, and "Y" is the amount of potassium permanganate added to the indicator blank.

Figure 2: *Excerpt from paper of interest describing the Lowenthal titration.* [6] *Reproduced with permission under CC BY-NC 4.0.*

Did You Know?

You may ask "what is the hide powder method"? Great question! It is a gravimetric method that mixes a source of dissolved tannins with a hide powder (which is an enzyme hydrolyzing protein produced from powdered animal hides) to see how the weight of the hide powder (before and after soaking with tannins) changes. This method seems odd, but it is well utilized still today!

Now, to calculate the percent tannin, we need to consider what the units of (weight) percent break down into:

$$\frac{\textbf{Grams of Tannins in Sample}}{\textbf{Total Grams of Sample}} * \mathbf{100} = \%\boldsymbol{tannins} \tag{4}$$

Firstly, how do we determine the grams of tannins in an unknown sample? In the titration, we do know the amount of MnO_4^- added. The volume of MnO_4^- added to the sample is the difference in volume titrated between the sample & indicator (X) and the indicator alone (*i.e.,* the blank titration) (Y). Now that we have the volume of MnO_4^- added, how do we convert THAT to tannins? Some sources have stated that the conversation factor from 1 mL of 1 N $KMnO_4$ into grams of tannin is 0.0416, as determined by the hide powder method.[7] The other aspect of this conversion is the use of normality as a unit of concentration rather than molarity (which we are more familiar with). Normality is related to the "reactive" units for a molecule. If a molecule only has one unit that is reactive (*i.e.,* the 1 H^+ in HCl), the normality and molarity would be the same. $KMnO_4$ (*i.e.,* MnO_4^-), in an acidic medium, donates 5 electrons, meaning it has 5 reactive units. Thus, a 1 M solution of $KMnO_4$ is equivalent to a 5 N solution. We can perform unit analysis to convert:

$$\frac{\textbf{mL of KMnO}_4}{\mathbf{1}}\left(\frac{\textbf{Z N KMnO}_4}{\textbf{1 mL of KMnO}_4}\right)\left(\frac{\textbf{0.0416 grams}}{\textbf{1 N}}\right) = \textbf{Grams of Tannins in Sample} \tag{5}$$

To make this conversion a bit easier, we can change this formula to consider molarity (W) rather than normality (Z) if we wish:

$$\frac{\textbf{mL of KMnO}_4}{\mathbf{1}}\left(\frac{\textbf{5W M KMnO}_4}{\textbf{1 mL of KMnO}_4}\right)\left(\frac{\textbf{0.0416 grams}}{\textbf{1 M}}\right) = \textbf{Grams of Tannins in Sample} \tag{6}$$

In the end, we can combine Equation (4) and (6) to derive:

$$\%\boldsymbol{tannins} = \frac{(\boldsymbol{X}-\boldsymbol{Y}) * \boldsymbol{W} * \mathbf{20.8}}{\textbf{Total Grams of Sample}} \tag{7}$$

In the paper, they almost have the same thing as Equation 7, but why is it slightly different? Well, their experiment utilized 1 mL of juice (their sample), meaning the bottom of our equation would just be "1", assuming 1 gram = 1 mL (which isn't always a good assumption). Then, on top, if we plugged in their approximate concentration of 0.005 M (which is 0.025 N), we get 0.104 as a constant on top, which, when inversed is ~10. Thus, the authors' math and our math are the same!

Performing a titration can be done in 2 ways: manually (with a colorimetric indicator) or automated (using a computer to plot volume vs. signal). There are pros and cons to each set up. While manual titrations are fast and inexpensive (not considering personnel costs) in comparison to the automated titration, the manual titration lacks information to be able to look at a titration curve and confirm that the endpoint of the colorimetric indicator (meaning where the color change happens) matches the equivalence point (where the quickest change in voltage is for a redox titration).

> ***Did You Know?***
>
> Titrations are still commonly used in industry today! Titrations are used to check acidity of solutions. They are also commonly used to determine the purity of compounds. The purity of the gallic acid you will use in this lab was determined through a titration!

The Lowenthal titration is particularly tricky, as different sources say different colors for the colorimetric endpoint, ranging from green to yellow to yellow with a red tinge. By using an automated titration set up, we can confirm with our own eyes the color of the equivalence point and then perform a manual titration to save time and resources!

Building a Method (Challenges and Nuances)

While we talked you through the process above, developing this method was challenging to say the least. Many times, the method was poorly explained in a paper/online resource, including in the paper above, and it took looking at multiple other papers published over a 50-year period to find the conversion factor and where it even came from! Furthermore, the units are a bit confusing: percent tannins. We know this came from the fact that they used tannic acid when determining that conversion factor (with the hide powder method), but, do all polyphenols react the same way? I wouldn't expect them to, especially when comparing structures of 2 common polyphenols, catechin and gallic acid, to tannic acid (**Figure 3**). So, we will explore that!

Speaking of Equation 7, the denominator includes the word "sample", but what exactly is the sample? The Lowenthal titration was originally developed for juices and ciders, so, it was natural to think about the sample as a volume of what you are analyzing. When thinking about a real-world sample (*e.g.,* apple juice), this is pretty simple. If needed, it may be useful to convert the bottom value to a volume. If we assume the solution is dilute enough, we can say that 1 gram is equivalent to 1 mL (*i.e.,* the density of water). Using this assumption, considering that parts per million (ppm) is equivalent to mg/L, you can easily convert to ppm from the above equation by multiplying by 10^4 (since $10^6/10^2=10^4$).

But, what if we are analyzing standards? The "total grams of sample" is related to the grams of the standard now; if we follow the same assumption as above, this would be the volume of the standard we add. We will be doing these comparisons over multiple days (and will remake the standards); thus, the volume will NOT have the same amount of standard each day. We need a better, more comparable number to calculate. Thus, for our purposes, we will convert Equation 7 to be total grams of polyphenol standards analyzed:

$$\boldsymbol{TAE = \frac{(X-Y)*W*20.8}{Total\ Grams\ of\ Polyphenol\ Standard}} \quad (8)$$

We will call this newly calculated value TAE (or tannic acid equivalents). The reason we want to make this distinction is to make this information comparable over multiple days/time periods or if multiple people analyze the same sample/standard.

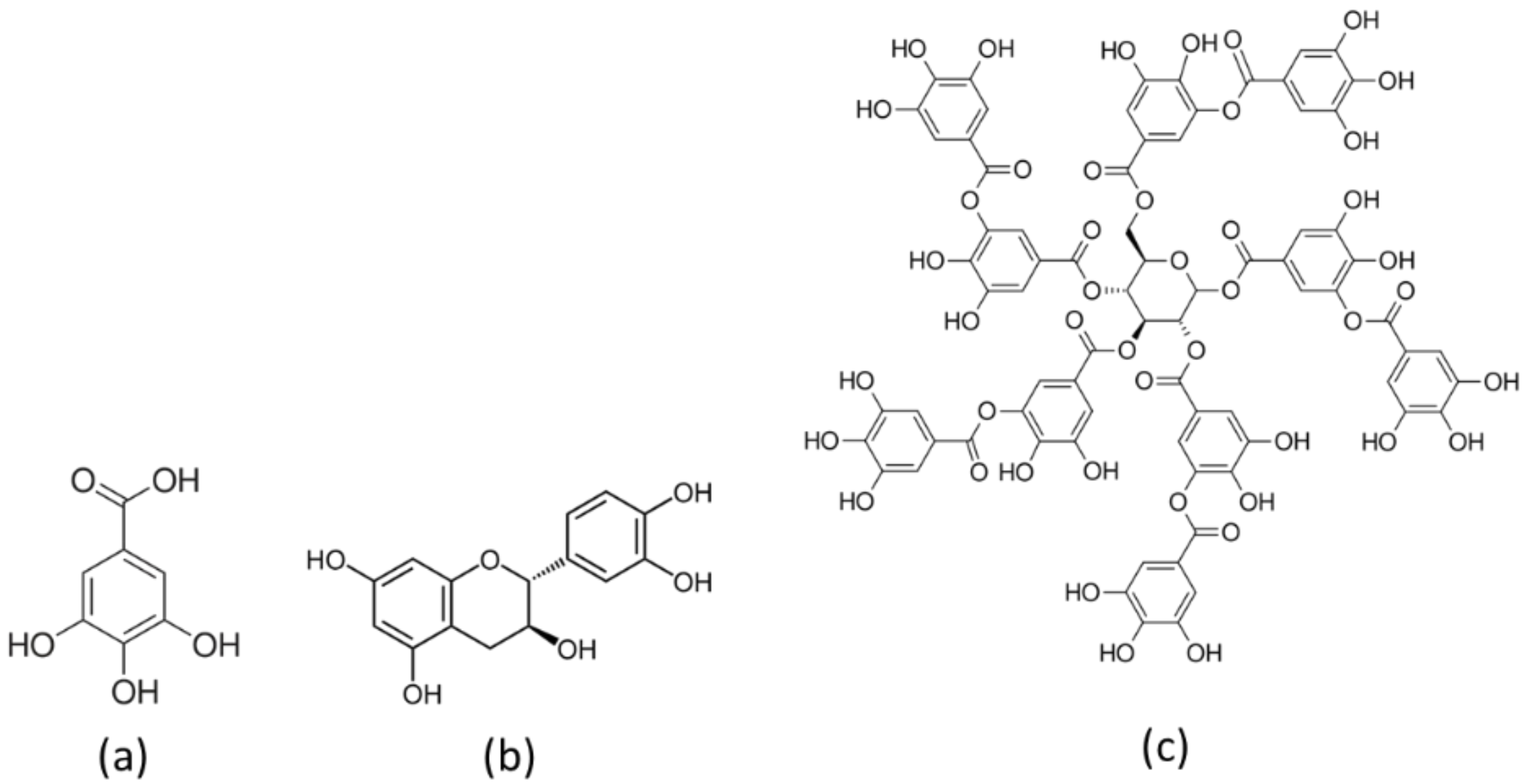

Figure 3: *Structures of different polyphenols. (a) Gallic acid. (b) Catechin. (c) Tannic Acid.*

Another mathematical challenge of doing lab work is being able to discuss the results with some confidence. This requires the use of statistical analysis to frame our results. Your textbook does a good job of discussing common options to make two major comparisons[4]:

(1) Comparing a sample population (Mean±SD) to a known value
(2) Comparing a sample population (Mean±SD) to another population (Mean±SD)

Your textbook also discusses how to handle outliers from that sample population (Mean±SD).[4] Those tests are summarized in **Figure 4**. Please note that the depth of statistical knowledge that exists in the world is FAR beyond what is discussed in this class. The options provided here and in the textbook are good starting points, but, if you find there is a better statistical test that exists, especially considering your experiences in other class or in research, feel free to use those! Examples we have seen used in this class include ANOVA, Q-Q plots, *etc.* You will need these tests to confirm not only if the known mole-to-mole ratio matches what you experimentally determine (see downward branch, **Figure 4**) but also if your data is reproducible and repeatable (see rightward branch, **Figure 4**).

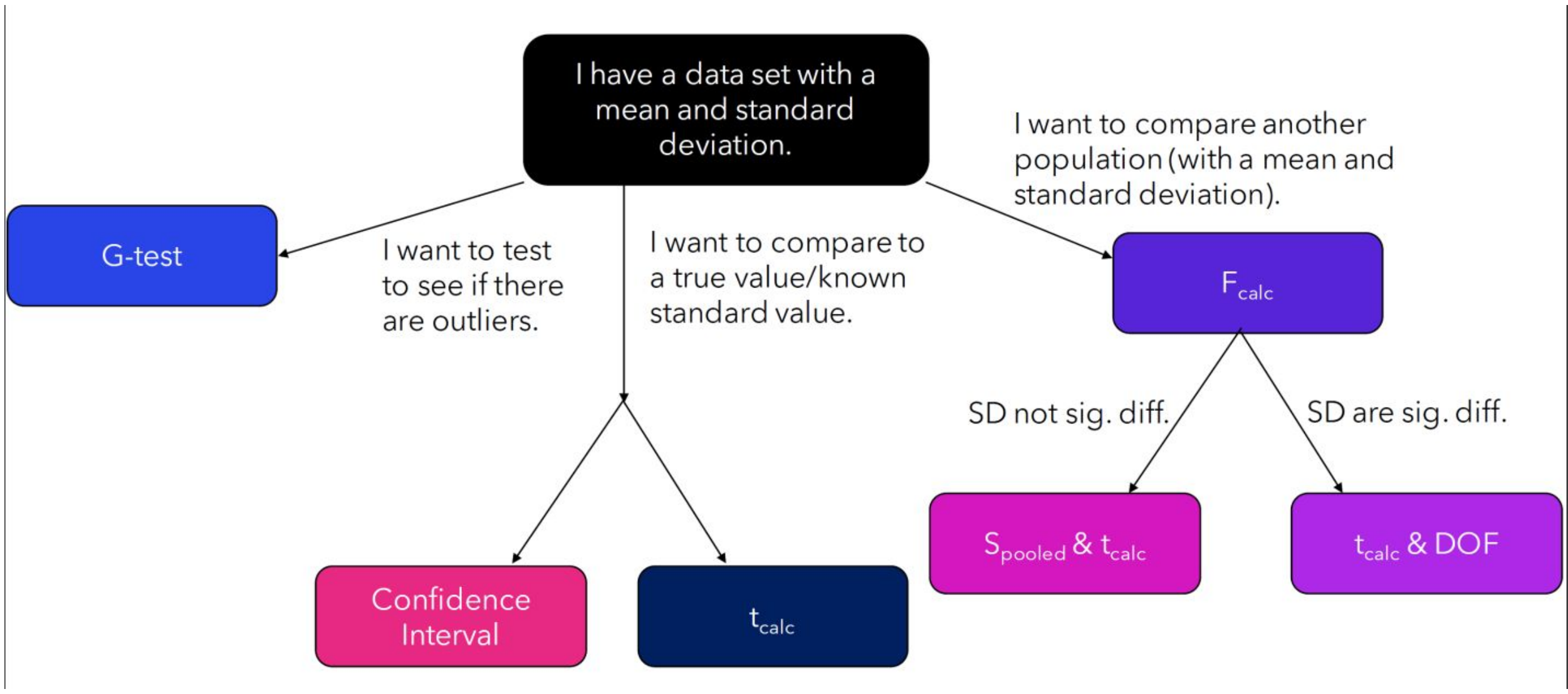

Figure 4: *A simple flow chart summarizing statistical tests commonly taught and used in our class.*

Safety Considerations

Whenever you develop a method, you want to make sure it can be done safely while minimizing other issues. We can look up the Safety Data Sheet (SDS) to find more information on these chemicals, especially if you haven't worked with those chemicals before. In particular, we are working with ***semi-dilute (5%)*** sulfuric acid. Make sure you look into how to handle this safely.

Deliverables

The overall project lab has the following deliverables:

1. Recognize and compare/contrast authentic, modern analytical methods used to analyze polyphenols.
2. Construct and justify a method to analyze and statistically test a hypothesis focused on sample of personal interest.
3. Analyze and validate results with appropriate statistical methods and quality assurance and control metrics, including detection limits, controls, and the percent recovery of a spike.
4. Summarize and communicate findings in an oral presentation and written abstract.

This lab is framing your project lab experience and ability to complete the project lab deliverables. The specific deliverables of this lab are:

1. Adapt a previously published analytical method for 2 standard polyphenols (catechin and gallic acid) to our laboratory setting.

2. Create a coherent procedure by exploring undefined procedural steps and connecting appropriate conceptual knowledge.
3. Evaluate a method's reproducibility (multiple people doing an analysis using their own equipment in a single day) and repeatability (single person doing an analysis using their own equipment across similar lab periods).
4. Apply statistical methods to frame results and determine if sample populations are equivalent

PRELABORATORY EXERCISES

Since the paper just lists concentrations and volumes, we are going to do our pre-lab preparation to make sure we know how to create those solutions!

1. Come up with a timetable to be able to complete the lab below. What checklist items should be completed each day? Note that you will have 40-50 mg of both catechin and gallic acid (per person) supplied to you TOTAL.
2. You will be doing multiple titrations, all of which we can estimate will use ~50 mL per titration plus some extra for cleaning. Noting that you should make fresh solutions every day, don't want excess waste, and that we want to use the glassware in your drawer, using the timeline you outlined above, plan how much volume you will need and how many grams of $KMnO_4$ that would require to make a ~0.005 M (N/40) solution.
3. Noting that you should make fresh solutions every day, don't want excess waste, and that we want to use the glassware in your drawer, using the timeline you outline above, plan how much volume and how many grams of indigo carmine that you would require to make the described solution (0.1%). You will have 50% (9 M) sulfuric acid available to you, and you should look at the paper for how much indicator will be used for each experiment.
4. Come up for a chemical reasoning if you observe a solid is formed at the bottom of your beaker as the titration occurs.
5. Show the work to derive Equation 3, proving the expected mole-to-mole ratio of both catechin and gallic acid.

BEFORE YOU TAKE THE QUIZ

The point of the pre-lab quiz is to make sure you are fully prepared for the experiment and understand the major concepts and calculations. Before taking the quiz, please review the posted equations and understand how to use them.

EXPERIMENTAL

Pro Tips

- All solutions should be made fresh daily to avoid degradation of the reacting species via light and oxidation.
    - $KMnO_4$ is known to slowly decompose when exposed to light or heat, making it a poor primary standard; we are assuming that the $KMnO_4$ is stable when we do not standardize it against a primary standard. This could cause an increase in variability of your results.
- You will be given a set amount of materials on the first lab period. You will not get more, so you need to think about how to plan those amounts BEFORE doing experiment. DO NOT GO INTO LAB UNTIL YOU HAVE A PLAN.

Key Solutions (as a Starting Point)

USE CAUTION!

- ***The sulfuric acid used in this experiment will burn you badly. Be attentive to adhering drops on the glassware. Remove sulfuric acid with a lot of tap water. Don't drink it. In case of eye contact wash the eyes with flowing tap water for 15 minutes, then go to the doctor.***

These are designed to get you started on the first experimental step. Feel free to update or change these depending upon your method development goes or what you need that day!

1. Potassium Permanganate ($KMnO_4$, 158.03 g/mol, 0.005 M (N/40)): Make this solution using your plan developed in your pre-lab questions.
2. Indigo Carmine Indicator (0.1%): Make this solution using your plan developed in your pre-lab questions.
3. Polyphenol Standard #1 (Catechin, 290.27 g/mol (anhydrous), 100 mg/L): Weight out 0.01 grams of catechin and dissolve in 15 mL of ethanol prior to diluting to 100.0 mL with DI water.
4. Polyphenol Standard #2 (Gallic Acid, 170.12 g/mol, 100 mg/L): Weight out 0.01 grams of gallic acid and dilute to 100.0 mL with DI water.

Pro Tip

Talk to the stockroom if you want to use other sized glassware than what is suggested!

Determination of Titration Endpoint (AS A GROUP OF 3-4)

This exploration will help us to define the colorimetric endpoint using a 'blank' titration.

1. Assemble your Venier automated titration set up using the drop counter and ORP probe.
    a. Wash the ORP probe with a 50:50 mix of methanol:water for 15 minutes prior to completing the titration.
    b. Calibrate the drop counter with your titrant ($KMnO_4$) to be ~1 drop/3 seconds to allow the ORP probe enough time to equilibrate.
2. In a 250 mL beaker, add 5.000 mL of indicator and dilute to ~150 mL with DI water. Add a large stir bar and begin stirring on the stir plate.
3. Titrate the sample beaker with $KMnO_4$, watching carefully to connect a solution color to when the equivalence point of the titration is.

Quantitative Analysis of Standard Samples (INDIVIDUALLY, BUT SHARE THE DATA WITH YOUR GROUP)
This experiment is to quantify the TAE of 2 standard polyphenol solutions. These experiments will also be used to confirm what the mole-to-mole ratio is for these two polyphenol solutions with potassium permanganate.

1. Using a buret, perform a direct, manual titration to determine the tannic acid equivalents of 25.00 mL of your catechin sample by performing at least 3 replicates of both a blank titration and a titration of the standard.
2. Using a buret, perform a direct, manual titration to determine the tannin equivalents of 25.00 mL of your gallic acid sample by performing at least 3 replicates of both a blank titration and a titration of the standard. **<u>HINT</u>**: If you can use the same blank titrations for gallic acid and catechin if they are run with the same solutions on the same day.
3. Repeat the catechin titrations (with blanks!) on a different lab day.

Pro Tip

It is quite possible that the concentrations of either the polyphenol solutions or the potassium permanganate are too dilute or concentrated to work within the optimal range of the buret (25-40 mL). Don't be afraid to change the concentrations of your stock solutions to reduce the error!

RESULTS/CALCULATIONS
Fill out the answer sheet for this experiment completely. Attach copies of your notebook pages and submit them to the LMS. Make sure your notebook pages include the following:

1. What color did you determine the titration endpoint to be? Include a copy of the acquired titration curve in your lab submission.
2. Calculate the amount of tannin acid equivalents (TAE, see Equation 8) in the catechin and gallic acid standards (including their standard deviations). Make sure you consider the AVERAGE volume

blank titrations performed. Are these numbers the <u>same</u> (using statistical analysis) on the same day? Explain why that statistical test was most appropriate for this situation.

3. Calculate the experimental mole-to-mole ratios for both gallic acid and catechin using the following equation:

$$\frac{Moles\ of\ Polyphenol\ (Catechin\ or\ Gallic\ Acid)\ Added}{Moles\ of\ KMnO_4\ Added - Moles\ of\ KMnO_4\ for\ Blank} = Ratio$$

4. <u>Statistically</u>, can YOU obtain the same results on different days for TAE of gallic acid or catechin? What about the mole-to-mole ratio? Explain why that statistical test was most appropriate for this situation.
5. <u>Statistically</u>, did your GROUP members determine the same TAE of gallic acid and catechin? What about the mole-to-mole ratio? Explain why that statistical test was most appropriate for this situation.
6. Write a 200-word abstract (in paragraph form) that captures the following items (1-2 sentences for each):
    a. What scientific (or general) background is important for us to understand and care about these experiments?
    b. What are we trying to determine? What technique are we using to answer that question?
    c. What color did you determine the endpoint to be? How did you determine/define it?
    d. What were the concentrations (TAE) ± standard deviation (SD) you found for each of our standards? Were they the same or different (statistically) (*e.g.,* include t-value comparisons, *etc.*)?
    e. What were the mole-to-mole ratio(s)±SD, and were they consistent with what you expected? If not, explain a potential reason why.
    f. Are your results (*e.g.,* TAE values, mole-to-mole ratios, *etc.*) reproducible across multiple days? Multiple individuals? Explain why/why not.
    g. What future directions do you see yourself taking this experiment? Are there changes you would make, for example? Or samples you think would be best analyzed via this method?

CHALLENGE QUESTIONS FOR YOUR LAB NOTEBOOK

Challenge questions are designed to make you think deeper about the concepts you learned in this lab. There may be multiple answers to these questions! Any honest effort at answering the questions will be rewarded.

1. Do you think MnO_4^- is reacting as expected (*i.e.,* as described in equation (2))? Explain your reasoning using any evidence you gathered in the lab.
2. This method is noted for measuring the total "tannin". But, you can also quantify the true amount of tannins by reacting your sample with gelatin or formaldehyde, which precipitates (and thus removes) the true tannins. Propose how you would modify your method and calculations above to account for the use of a gelatin control sample.

3. Considering consistency, the catechin sample we purchase is actually not pure catechin; it is a catechin hydrate with an unknown amount of H_2O molecules. When analyzing catechin from the same bottle, while the moles of catechin will have an unknown amount of impurity (we estimate ~2%), it is quite possible that catechin from different batches or companies will have different hydration amounts. <u>We assumed that the catechin is anhydrous (contains no water) for our purposes in lab.</u> Provide a quantitative explanation for how using a catechin hydrate instead of a pure catechin standard have impacted your resulting, including the measured TAE for the catechin standard, the mole-to-mole ratio with $KMnO_4$, and your repeatability of the measurements across multiple days.

REFERENCES

(1) El-Saadony, M. T.; Yang, T.; Saad, A. M.; Alkafaas, S. S.; Elkafas, S. S.; Eldeeb, G. S.; Mohammed, D. M.; Salem, H. M.; Korma, S. A.; Loutfy, S. A.; Alshahran, M. Y.; Ahmed, A. E.; Mosa, W. F. A.; Abd El-Mageed, T. A.; Ahmed, A. F.; Fahmy, M. A.; El-Tarabily, M. K.; Mahmoud, R. M.; AbuQamar, S. F.; El-Tarabily, K. A.; Lorenzo, J. M. Polyphenols: Chemistry, Bioavailability, Bioactivity, Nutritional Aspects and Human Health Benefits: A Review. *Int. J. Biol. Macromol.* 2024, *277*, 134223. https://doi.org/10.1016/J.IJBIOMAC.2024.134223.

(2) Câmara, J. S.; Albuquerque, B. R.; Aguiar, J.; Corrêa, R. C. G.; Gonçalves, J. L.; Granato, D.; Pereira, J. A. M.; Barros, L.; Ferreira, I. C. F. R. Food Bioactive Compounds and Emerging Techniques for Their Extraction: Polyphenols as a Case Study. *Foods 2021, Vol. 10, Page 37* 2020, *10* (1), 37. https://doi.org/10.3390/FOODS10010037.

(3) *Methods for analysis of Cider Tannin*. https://www.cider.org.uk/tanmeths.htm (accessed 2026-04-27).

(4) Harris, D. C. .; Lucy, C. A. . *Quantitative Chemical Analysis*; Macmillan Learning, 2020.

(5) Baranowska, M.; Suliborska, K.; Chrzanowski, W.; Kusznierewicz, B.; Namieśnik, J.; Bartoszek, A. The Relationship between Standard Reduction Potentials of Catechins and Biological Activities Involved in Redox Control. *Redox Biol.* 2018, *17*, 355. https://doi.org/10.1016/J.REDOX.2018.05.005.

(6) Miles, C. A.; King, J.; Miles, C. A.; King, J. Yield, Labor, and Fruit and Juice Quality Characteristics of Machine and Hand-Harvested ‘Brown Snout’ Specialty Cider Apple. *Horttechnology* 2014, *24* (5), 519–526. https://doi.org/10.21273/HORTTECH.24.5.519.

(7) Barua, D. N.; Roberts, E. A. H. Methods for the Volumetric Estimation of Tea Tannin in Green-Leaf and Black Tea. A New Alkaline Permanganate Method. *Biochemical Journal* 1940, *34* (12), 1524–1531. https://doi.org/10.1042/BJ0341524.
