# Supplementary material for: Pick Your Own Adventure: Orthogonal Methods for Analyzing Polyphenols
Source: J Chem Educ. 2026 Jul 17;103(8):4243–54. doi: 10.1021/acs.jchemed.5c01459 (PMC13480120; doi:10.1021/acs.jchemed.5c01459)
Supplement: Supplementary file 7 [file ed5c01459_si_004.docx]

**Pick Your Own Adventure: Orthogonal Methods for Analyzing Polyphenols**

Amanda Rae Buchberger^1^*, Kristel M. Forlano^1^, Madeline J. Lund^1^, Pamela S. Doolittle^1^, Dominic M. Colosi^1^, Thao U. Duong^1^, Brian J. Esselman^1^, Kyoung-Shin Choi^1^, Robert J. Hamers^1^

^1^Department of Chemistry, University of Wisconsin-Madison, 1101 University Avenue, Madison, WI 53706

*Email Addresses:

[amanda.buchberger@wisc.edu](mailto:amanda.buchberger@wisc.edu)

# Lab Manual for Chromatographic Separation of Polyphenols using HPLC (CH) Lab Module

# THE TWO PHASES OF SEPARATION: USING LIQUID CHROMATOGRAPHY AND POLARITY TO ANALYZE POLYPHENOLS

*In this lab, students will learn about one option (chromatography) for quantifying polyphenols. Students will prepare their own controls and samples to explore how different instruments, separation conditions, and detectors influence the analysis of common polyphenols, catechin and gallic acid.*

Visual Abstract:

To cite this lab manual: “The Two Phases of Separation: Using Liquid Chromatography and Polarity to Analyze Polyphenols”. A Manual of Experiments for Analytical Chemistry. Department of Chemistry at UW- Madison, Spring 2024.

*Did You Know?*

Keep an eye out for these green boxes throughout your lab manuals! They will contain little tidbits connecting the experiments you’re completing with real research and industrial applications, provided by Dr. Bradley Bolling in the Department of Food Science at UW-Madison and Dan Berg, an Analytical Services Manager at Eurofins Food Testing Chemistry in here in Madison!

BACKGROUND

Just like there are many tools in a toolbox, the analytical chemist has a plethora of chemistries, techniques, and instrumentation to identify and quantify analytes of interest. Qualitatively, we must first discern WHAT needs to be measured, and knowing that, can select and characterize an appropriate method for analysis. In this lab series, we will explore a variety of techniques used to quantify polyphenols. This manual focuses on a chromatography-based methodology to isolate, identify, and quantify various polyphenols.

Importance

Generally, polyphenols are a large family of molecules ([>8,000 identified](https://www.chhs.colostate.edu/krnc/monthly-blog/what-are-polyphenols-another-great-reason-to-eat-fruits-and-veggies/)) that (as you guessed) contain many phenol (C_6_H_5_-OH) groups.^1^ They are incredibly diverse, ranging in size from ~100 to ~800 Daltons (~g/mol). There are different classes (and structural motifs). Generally, non-flavonoids contain only one phenol ring, and flavonoids contain two phenol rings. Polyphenols are naturally found in many fruits, vegetables, and teas. Interestingly, the pulp of fruits/vegetables is rich in non-flavonoid compounds, while the skins, seeds, and stems are rich in flavonoid compounds. Green tea has gained a lot of attention for its high concentrations of the polyphenol epigallocatechin gallate (EGCG).^2^ Easily purchasable and simple polyphenols that you will work with in this lab are catechin (a flavanol) and gallic acid (a non-flavonoid, phenolic acid). The great variety in structures makes analyzing these molecules difficult without customizing or restricting the exact samples analyzed.

On a more microscopic scale, polyphenols are known primarily as antioxidants, meaning they inhibit oxidation. Uncontrolled oxidation inside the human body (*e.g.,* of DNA, proteins) results in cellular damage that can lead to downstream problems. Antioxidants react with these oxidizing species (*e.g.,* oxygen) to reduce their oxidative behavior and therefore reduce cellular oxidation in the body. Many people associate consuming antioxidants with reducing the effects of aging and mitigating risks of certain cancers and other diseases.

Building a Method (The Important Stuff)

The literature is a great place to find inspiration and pre-established methods. In the realm of chromatography, especially for polyphenols, there is a wide variety of methods to choose from. Typically, the method is selected by the researcher based on the equipment available in the lab. Thus, before we can select our method, we need to understand what we have available to us in the lab (and the theory behind each of those pieces of equipment). Fundamentally, chromatography is a general method for separating molecules of interest. Once separated, the molecules can be detected through an analytical measurement that is selected based upon the molecules of interest.

Most chromatographic separations have 2 phases that separate the molecules of interest: (1) a stationary phase and (2) a mobile phase. The stationary phase is, as you guess, stationary/fixed, while the mobile phase is “washed” over the stationary phase. Molecules of interest are given the chance to selectively interact with or ‘partition’ between the stationary or mobile phase. Essentially, this is an equilibrium. Thus, these 2 phases should be sufficiently different in some chemical property (*e.g.,* polarity, charge, *etc.*) to help the molecules select their preferred phase. The stationary phase could be put onto a piece of paper (*e.g.,* thin layer chromatography, Figure 1a), put into a glass or plastic tube (*e.g.,* flash column, Figure 1b, or solid phase extraction, respectively), or quantitatively packed in a commercial stainless-steel tube (*e.g.,* analytical column, Figure 1c), the latter being used for modern instrumentation. These analytical columns are generally made from 4.6 mm ID stainless steel tube with heavy sidewalls. The columns are typically 10 to 15 cm long, and packed with surface modified porous silica microspheres, while the flash columns and TLC plates are variable in length, depending upon the separation needed or the complexity of the sample. Analytical columns being coupled to modern day instruments make them more useful for real-world, complex samples both quantitatively and qualitatively, which the other 2 methods do not do well, making them more “preparatory” or done to provide a crude separation in preparation for another experiment.

*Figure 1: Pictures of the stationary phase and its support for different types of chromatography. (a) Plates used for Thin Layer Chromatography (TLC). (b) Flash column filled with a stationary phase (gray). (c) Analytical column packed with stationary phase of interest.*

If one of our goals is to quantify these molecules, the use of modern-day instruments and analytical columns seems to be a requirement. In our laboratories, we have two classes of chromatography instruments: gas chromatographs (GCs) and liquid chromatographs (LCs). Both instruments use columns that contain a solid stationary phase; the difference between GC and LC is the mobile phase (and thus the phase the molecules of interest need to be in to be analyzed). If your molecule is in liquid form, using LC is ideal; same principles should be used if the molecule can be volatized or prefers to be a gas for GC. Polyphenols are not known for being molecules that like to be gaseous without a modification^3^, so the use of liquid chromatography seems to be the best fit here. To improve separation and the time for separation, modern day LCs use high pressure, meaning the instruments are usually called high pressure liquid chromatographs (HPLCs). You could also see the “P” in this acronym be “performance”.

*Did You Know?*

Gas chromatography is commonly used in the food science industry for measuring flavor volatiles. Many compounds that are less volatile can be derivatized so they now exist in a gaseous state and are able to be analyzed through GC (*e.g.,* fatty acids).

In general, modern HPLCs can be relatively simple or complex. Overall, each system needs 5 things (Figure 2b):

(1) a supply of mobile phase (and a way to pump that mobile phase)
(2) a sample introduction/injection system, to place the sample in the flowing mobile phase ahead of the column
(3) a column or support containing the stationary phase, and through which the mobile phase can flow
(4) a detection system, to monitor component concentrations in the effluent
(5) a recording system (which is usually a computer!) to make a permanent record of the concentration of solute vs. time or volume of effluent

Some of these items can be manual vs. automatic (like the sample injection system). Some of these can have multiple versions (like the pumps or the detectors) in a single instrument to allow more control or more information, respectively, to be obtained. The detection system is critical; even if a molecule can be separated, it needs to be detected to be identified or quantified. Choosing the detection method depends upon how selective or specific you need it to be, and any analytical measurement technique available can be used as a detector. Also, the stationary phase and mobile phase should also be customized based upon the molecules being separated. In the realm of LC/HPLC, the most common property used to separate the molecules is polarity. If the stationary phase is polar in comparison to the mobile phase, this is considered a “normal-phase” (NP) separation. More commonly, though, for biological molecules, the polarity of the mobile phase and stationary phase are flipped (*i.e.,* polar mobile phase in comparison to stationary phase), which is called a “reversed-phase” (RP) separation. The stationary phase can be modified by adding octal, amino, nitrile, phenyl, or methyl groups to change the desired strength the polarity.

In our lab spaces, we have two HPLC options (**Figure 2a or 2c**), both of which are the instrument brand Shimadzu. The first system (**Figure 2a**), called the Shimadzu 10 series (which we will call the HPLC), is simple, only having 1 mobile phase pump (meaning the mobile phase does not change during the separation, which is called an isocratic elution), a manual injection system, a C8 column (meaning it has a C_8_H_17_ chain on the silica support), and an absorbance detector (mobile phase blank). The second system (**Figure 2c**), called the Shimadzu Nexera XR Series (which will we will call the ultraHPLC, or uHPLC), is a bit more complex, including 2 mobile phase pumps (meaning we can have the mobile phase change over the course of the separation, called a gradient elution), an autosampler (meaning we can inject 105 samples in a row without human intervention), a C18 column (meaning it has a C_18_H_37_ chain on the silica support), and both an absorbance (mobile phase blank) and fluorescence detectors available. So, they use the same chemistry (reversed-phase-based separation), but they couldn’t be more different otherwise! We do not say anything about the mobile phase here, but you can assume that the mobile phase, to have a reversed-phase separation, is a mix of polar solvents, such as water, acetonitrile, methanol, *etc*. For a gradient elution, it is most common that the mobile phase composition changes to be more like the stationary phase, or, for RP-HPLC, more polar, over the course of the separation.

*Figure 2: Schematic of our 2 different HPLC systems ((a) HPLC and (c) uHPLC) and the basic components they contain and how they are connected to each HPLC systems (b).*

Generally, the following steps are done for a chromatographic separation, no matter the equipment:

(1) Prepare the stationary phase *(if dry, it will be unavailable for molecules to interact with it)*
(2) Equilibrate the stationary phase *(so that we have a baseline reading/signal)*
(3) Bind *(to the stationary phase by injecting the sample)*
(4) Wash *(away the other stuff you don’t care about)*
(5) Elute *(meaning release or measure the molecules interest purposefully (e.g., to the detector))*

The data collected after elution is called a chromatogram (**Figure 3**), which is a plot of signal (absorbance, intensity, conductivity, *etc.*, depending upon the detector used) vs. retention time (seconds to hours). As a molecule is separated, comes off the column, and then is detected, this results in a signal on the chromatogram, shaped like a Gaussian peak. Ideally, each molecule would have its own peak and a consistent retention time (which can be used to tentatively identify the molecule), dependent upon the molecule’s interactions within the column. Once a retention time of a molecule has been established, the area under the curve or the peak height is thought to be correlated with the amount/concentration of the molecule present (assuming we are working in the linear range of that molecule, just like with Beer’s Law).^4^ That means we can use techniques like calibration curves, standard addition, and internal standards to perform the quantitative analysis. Check out Chapter 18 of your textbook^4^ for more information about each of these techniques. We traditionally use calibration curves for our HPLCs, meaning we inject 5 known, differing amounts (*e.g.,* 5 injections) of the pure standard, obtaining 5 chromatograms with single peaks at the approximately same retention times that have different peak areas/heights. If we plotted the area/height of the peaks vs. the known concentration, we would obtain a calibration curve (using “y=mx+b” equation) that could be used to quantify an amount of a molecule found in a sample. In determining the peak area and height, modern day software does a lot of the work for us by automatically measuring the height or integrating under the peak, but, before computers, one method was the “cut-and-weigh” method, where the chromatogram was physically recorded on a piece of paper, and each peak of interest was cut out and weighed^5^, although, this method is generally inconsistent due to the variations in paper thickness.^6^

*Figure 3: An example chromatogram^modified with permission from 7^ with key parameters, such as retention time, peak height, and peak area marked. Note that peak area has arbitrary units.*

While you will not be able to define the calibration curve range on your own (you’re welcome for doing that for you!), to analyze a real-world sample, a lot of choices must be made. What concentration are we going to assume our sample is at? What are we going to do to prepare samples to meet the linear calibration range? This is especially hard because “polyphenols” include a wide variety of molecules! If a reported concentration for a sample is 25 mg of polyphenols, which polyphenols could it be?! Do we assume it is composed of only one polyphenol? Or a mix based upon the literature? Whatever choices you make, it is important to note that method development involves many iterations of trial and error, and it’s okay to change your procedure on the fly if things aren’t working as you expect. In the end, your goal should be to get your sample in the MIDDLE of your calibration curve (to reduce experimental uncertainty, see Figure 4) and make sure you report appropriate units. We will have multiple calibration curves (at least one for each polyphenol of interest that we analyze).

*Did You Know?*

“99% of the time, you’re not testing necessarily for [general] polyphenols”

- *Dan Berg*

Testing for specific compounds in a product can be much more useful for a company. HPLC is a very common method to analyze content of specific polyphenols, particularly catechins.

*Figure 4: Example calibration curve.*

Since we have 2 different HPLCs we can use, why would we use one over another? Many times, researchers take the “path of least resistance”, meaning, if someone else has done it with a specific set up/instrument, they will just do the same. We also recommend using the least complicated instrument needed for your application. Some analytical considerations (to make that choice) could include costs (including the instrument purchase amount and any maintenance costs, or the cost-per-sample considering instrument consumables and preparation reagents), molecule sensitivity or selectivity, environmental impact, availability, and even personal preferences. Yes, it is VERY cool to use the newest and best instrument with all the fancy buttons and features, but, the more buttons or features, the more items that can fail. Instead, the KISS (Keep It Super Simple) principle is helpful to reducing your time, energy, and stress, as long as it meets your analytical goals and requirements!

Digging into polyphenol literature, RP-HPLC is by far the most prominent chromatographic technique. We are lucky to have found an article^7^ that shows multiple examples of RP-HPLC analysis (**Figure 5**). Let’s dissect this table a bit more:

(1) In the “Column” column of the table, we can see that all of the separations use a C18 column (*i.e.,* a non-polar stationary phase), meaning RP-HPLC was used.
(2) In the “Condition” column of the table, we see information about the mobile phase, what type of elution was used (gradient or isocratic) (including the time frame and mixtures of the different mobile phases used), the wavelength (which is the wavelength measured to detect analytes coming off), and the flow rate.
    a. Each mobile phase is a little different. 2 of the 3 separations use a gradient elution. One situation uses an isocratic gradient.
    b. The wavelengths measured are 210 or 270 nm, which is in the UV-Visible range of the electromagnetic spectrum. Most HPLC systems have an absorbance detector. How are these determined though? Many times these wavelengths are found in the literature or determined experimentally. It is worth noting that phenol in hexane alone absorbs between 200-300 nm.^8^
    c. The flow rate is really specific to the instrument. You want the flow rate to be set to make sure there is enough pressure (remember HPLC) to push the sample to the column, through the column, and then to the detector without impacting how well things separate (or damaging the instrument).

*Figure 5: Table^reprinted with permission from 7^ outlining different separation conditions for polyphenols, including catechin and gallic acid.*

Ok, so which separation parameters do we use (or at least optimize for our instrument)? And which instrument do we need to use? Well, it depends upon what we are trying to analyze. Two readily available standards are catechin (labeled “C” in **Figure 3**) and gallic acid (labeled “GA” in **Figure 3**). Their structures are shared in **Figure 3**. When working with these standards alone, we will only have 2 things, so we really don’t need a complex instrument. But, what about when we are analyzing a real sample? We will answer these questions (and more) as we complete this lab!

Building a Method (Challenges and Nuances)

Unlike the other instruments you have worked with in this class, the detail and care that needs to be put into the analysis is more time consuming and requires rigorous knowledge and training due to the sensitivity and specificity of the settings of the instrument. These instruments are also much more expensive than you would expect; the HPLC set up we have Figure 2a is ~$20,000, while our uHPLC Figure 2is ~$100,000! Some HPLC systems cost even more! Maintenance on these instruments also includes specialized parts, engineers, *etc.*, which also cost a lot of money. In contrast, the Vernier SpectroVis Plus instruments we use in lab are ~$400, and the Vernier pH probes are ~$100. The columns alone for our HPLC systems are ~$500-1000. The column packings are easily clogged by particulate material. If your samples include particulate or viscous material, you must filter them before injection. If the column becomes clogged, it isn’t useable anymore and must be replaced. One way to avoid this issue is by using a pre-column (which is a short column prior to the analytical column), but only our uHPLC has that currently installed.

One area that is greatly affected by mindful care of the instruments is the peaks themselves. Without peaks, we cannot really identify or quantify anything. Thus, the column and mobile phase need to be carefully equilibrated (*i.e.,* stabilized) and made, respectively. Column packing materials are susceptible to mechanical shock, so columns should be handled with care. It is common to allow several minutes for changes in the mobile phase flow rate or mobile phase composition so the column can re-equilibrate (to make sure enough solvent volume has been pumped through the column) before running samples. Non-uniform peak shapes, such as those in GA (gallic acid) in **Figure 3**, are an example of what a peak could look like if something isn’t fully optimized in the system. This leads to poor quantitation or possible overlapping peaks. Ideally, in a separation, you will have highly-resolved peaks, meaning they are fully separated at the baseline. Overlapping peaks means that quantitation may not be as accurate, especially when considering the area under the curve. High resolution is the goal; the better the peaks are separated or resolved, the more accurate the peak area. If you have 2 peaks overlapping, you likely have some error due to inaccurate integration of the peak area, meaning peak height may be a more accurate metric to use.

If the mobile phase isn’t consistent (or the stationary phase isn’t fully equilibrated with the mobile phase), the retention times of the molecules of interest can also fluctuate. This isn’t ideal, as the retention time is the only metric we have to identify a molecule with our current set up(s), and retention time is not unique to a molecule of interest. That means a change in retention time would mean that you could either not identify your molecule OR misidentify your molecule. Yikes! This is very prominent in our example chromatogram (**Figure 3**), where Caf (caffeine), GCG (gallocatechin-3-gallate), and EC (epicatechin) all have the exact same retention time (*i.e.,* co-elution). Interestingly enough, the only difference between catechin and epicatechin is their stereochemistry! In this situation, what we would identify is there? How can we quantify this species with confidence? To be able to answer those questions, we can either (a) change the separation method to confidently resolve these species or (b) use a detection method that can measure multiple molecules simultaneously but separately (meaning co-elution or unresolved peaks can be overcome). Beyond just retention time, more definitive identification can be done via other detectors after the column, such as a mass spectrometer (which analyzes the mass of an analyte; *see Appendix A*) or infrared spectrometer (to analyze the functional groups present).

While we only have access to the absorbance detector on our HPLC, the uHPLC has 2 detectors: (1) absorbance and (2) fluorescence. Fluorescence, by definition, is a type of emission (when a molecule becomes excited by absorbing a photon and then releases/emits energy that then can be measured). While many molecules can absorb light in the UV-Vis range, there is a smaller subset of molecules that can fluorescence. This means that, by using a fluorescence detector, we may be able to be more selective about what molecules we are analyzing. More selective means (hopefully) a cleaner chromatogram, which means (hopefully) more accurate quantitation. It is also generally true, due to the fundamental differences between fluorescence and absorbance^4 (Chapter 18)^ , that the lowest measurable concentration is smaller for fluorescence, meaning it will be more sensitive to smaller concentrations in your sample. When setting up the instrument for absorbance, we just need to set 1 wavelength: the absorbance wavelength. For the fluorescence detector, we need to set 2 wavelengths: the excitation wavelength (*i.e.,* which wavelength does the molecule become excited, meaning absorb light) and the emission wavelength (*i.e.,* which wavelength will we measure as our actual output). Because you cannot emit more energy than you absorb, naturally the 2^nd^ (emission) wavelength will be a longer, lower energy wavelength.^4 (Chapter 18)^

Especially with this complex instrumentation, we cannot ignore quality assurance metrics. To know that the method can accurately measure the analyte of interest, use a positive control, meaning create a solution of approximately the same concentration (using a standard) as you would expect of your sample to make sure you measure that expected amount. (If you make a solution that is “X” mg/L and it measures not “X” mg/L, something is likely wrong!) Another metric of quality is the ability to replicate the results. Without replicates (and thus having an average and standard deviation), we will never have a firm understanding of the population and cannot perform statistical testing. There are 2 different types of replicates: (1) technical replicates or (2) biological replicates. Technical replicates are (a) injecting the same exact solution multiple times or (b) repeatedly preparing the same sample (and analyzing each replicate). This is a measure of how well the instrument replicates results. Biological replicates are injecting different samples or solutions prepared the same way to determine the natural variation in a sample. (Food stuffs does have “biology” involved, remember.) For example, we can make 3 solutions at 5 mg/L each rather than just inject the same 5 mg/L solution each time. This is a better representation of the variation in the SAMPLE rather than the INSTRUMENT. When we say statistical testing, we want to prove (statistically) (or disprove) that our population (mean and standard deviation) either contains a specific point or is the same as another population. Please reference your textbook for good options for statistical tests.^4^ The type of statistical hypothesis we are trying to test will influence what type of replicates we collect.

Finally, preparing real samples is not straightforward, as many real samples are not a soluble powders or aqueous liquids. In this lab, the samples you are going to be working with are commercially-made tea bags (which are filled with loose tea). How will you prepare these to extract the polyphenols? How can you estimate the amount of polyphenols found in these tea bags?

Safety Considerations

Whenever you develop a method, you want to make sure it can be done safely while minimizing other issues. We can look up the [Safety Data Sheet (SDS)](https://chemicalsafety.com/sds-search/) to find more information on these chemicals, especially if you haven’t worked with those chemicals before.

Deliverables

The overall project lab has the following deliverables:

1. Recognize and compare/contrast authentic, modern analytical methods used to analyze polyphenols.
2. Construct and justify a method to analyze and statistically test a hypothesis focused on sample of personal interest.
3. Analyze and validate results with appropriate statistical methods and quality assurance and control metrics, including detection limits, controls, and the percent recovery of a spike.
4. Summarize and communicate findings in an oral presentation and written abstract.

This lab is framing your project lab experience and ability to complete the project lab deliverables. The specific deliverables of this lab are:

1. Adapt a previously published analytical method for 2 standard polyphenols (catechin and gallic acid) to our laboratory setting.
2. Create a coherent procedure by exploring undefined procedural steps and connecting appropriate conceptual knowledge.
3. Assess a chromatography method’s results via evaluating replicates for consistency in retention time and quantitative accuracy using statistics.
4. Identify the capabilities and limitations of different instruments and instrument settings for the success of a chromatographic separation.

**PRELABORATORY EXERCISES**

*We are going to do our pre-lab preparation to make sure we know how to create our solutions and make predictions about the results we will see!*

1. Come up with a timetable to be able to complete the lab below. What checklist items should be completed each day? Note that you will have 40-50 mg of both catechin and gallic acid supplied to you TOTAL for your GROUP. *(You will need WAY less than that!)*
2. This pre-lab question is designed for you to prepare for analyzing real samples in lab.
   a. Search through the literature (or Google) to find an estimate of how much catechin and gallic acid you will find in brewed black and green tea (since you can be given either!). Make sure you include your citation in your lab notebook; try to pick a citation that is reputable.
   b. Come up with a plan on how to prepare the green and black tea *(HINT: there is a standard procedure for this)* to extract and also dilute to the appropriate concentrations, based upon the calibration ranges for BOTH instruments shared below (**Table 1 & 2**). You will want at least 1 mL of your sample prepared for each instrument.
   c. Come up with a plan on how to prepare a positive control mixture (to confirm retention times) that includes both catechin and gallic acid using the calibration ranges in **Table 1 & 2** below as a guide. You will want at least 1 mL of your sample prepared for each instrument.
3. Search through the literature (or Google) to find wavelengths for excitation and emission for both catechin and gallic acid. Make sure you include your citation in your lab notebook; try to pick a citation that is reputable. Does it match what is shared below in **Table 1 & 2**? Suggest a reason why or why not.
4. Propose a reason why absorbance and fluorescence data would provide different results.

**BEFORE YOU TAKE THE QUIZ**

*The point of the pre-lab quiz is to make sure you are fully prepared for the experiment and understand the major concepts and calculations. Before taking the quiz, please review the posted equations and understand how to use them.*

**EXPERIMENTAL**

*Pro Tips*

- All solutions should be made fresh daily to avoid degradation of the reacting species via light and oxidation.
- You will be given a set amount of materials on the first lab period. You will not get more, so you need to think about how to plan those amounts BEFORE doing experiment. Also, you will sign up for shifts below for a set amount of time to run on the instrument. DO NOT GO INTO LAB UNTIL YOU HAVE A PLAN.

**Key Solutions & Instruments**

*These are designed to get you started on the first experimental step. Feel free to update or change these depending upon your method development goes or what you need that day!*

***Table 1****. HPLC parameters for the analysis of polyphenols catechin and gallic acid.*

| Column | Pinnacle DB C8 HPLC Column, 150 cm x. 4.6 mm I.D., 5 µm particle size |
| --- | --- |
| Mobile Phase(s) | Solvent A: 45% Methanol + 5% Acetonitrile + 50% 0.1% Acetic Acid  *(~50:50 Mix of (3) in* ***Figure 6****)* |
| Gradient Elution | N/A (Isocratic Elution) |
| HPLC System | Shimadzu 10-Series (SPD 10A UV-Vis Detector; LC-10 AT Pump) |
| Detector Systems | UV detection at 270 nm |
| Flow Rate | 0.1000 mL/min |
| Pressure | 140-200 PSI |
| Column Temp | 25 °C (Room Temperature) |
| Injection Volume | 20 uL (Sample Loop) |
| Run Time | Standards: 2 minutes  Tea Samples: 5 minutes |
| (Provided) Calibration Curve | Catechin: 10-1000 mg/L  Gallic Acid: 1-200 mg/L |

***Table 2****. uHPLC parameters for the analysis of polyphenols catechin and gallic acid.*

| Column | - Sigma-Aldrich TitanTM C18 UHPLC Column, 10 cm x 2.1 mm I.D., 1.9 μm particle size - TitanTM C18 HPLC Guard Cartridge, 5 mm x 2.1 mm I.D., 1.9 μm particle size |
| --- | --- |
| Mobile Phase(s) | Solvent A: 0.1% Acetic Acid  Solvent B: HPLC Grade Methanol  Solvent C: HPLC Grade Acetonitrile |
| Gradient Elution | 0-1 minutes 18% B/4% C, 1-3 minutes ramp to 90% B/10% C; 3-6 minutes hold 90%B/10%C; 6-7 minutes return back to 18% B/4% C; 7-20 minutes hold 18% B/4% C |
| HPLC System | Shimadzu Nexara XR uHPLC |
| Detector Systems | UV detection (D_2_ lamp) at 270 nm; Fluorescence excitation at 280 nm and emission at 310 nm |
| Flow Rate | 0.1300 mL/min |
| Pressure | 3000-5000 PSI *(depending upon column cleanliness)* |
| Column Temp | 25 °C (Room Temperature) |
| Injection Volume | 2 uL |
| Run Time | 20 minutes |
| (Provided)  Calibration Curve | Catechin (UV): 10-1000 mg/L  Catechin (Fl): 1-400 mg/L  Gallic Acid (UV): 10-500 mg/L |

1. Polyphenol Standard (Catechin & Gallic Acid Mix; Positive Control): Make this solution using your plan developed in your pre-lab questions.

*Pro Tips*

- Talk to the stockroom if you want to use other sized glassware that isn’t available in your drawer.
- All HPLC calibration standards will be ran fresh day-of student samples being run. Please make sure to use the correct calibration curve information for submitted/collected data.

**Computational Understanding of the Data**

*This exercise will help you to understand the different forms of data that exist and how to analyze each type of data set.*

You will receive the following information/data on Canvas:

1. Data processing software (and instructions for how to use software).
2. Individual spectra for the calibration curves for the HPLC and a report that outlines the results from the uHPLC (both detectors).
3. Green tea tablet (**Figure 6**) data for the HPLC and the uHPLC (both detectors).
   a. For the HPLC, the green tea tablet was prepared by dissolving the contents of the tablet in 100 mL of 10% ethanol using a volumetric flask. The following solution was filtered prior to being diluted further by taking 3 mL and diluting to 10 mL in a volumetric flask. This resulting sample was analyzed.
   b. For the uHPLC, the green tea tablet was prepared by dissolving the contents of the tablet in 100 mL of 10% ethanol using a volumetric flask. The following solution was filtered prior to being analyzed.

Figure 6: *Relevant photos of green tea tablets, the real sample for this lab. (a) Front of bottle. (b) Nutrition facts on back of bottle. Feel free to ask your TA or the stockroom to see the bottle if needed.*

You should show your TA the following information for each instrument/detector combination):

1. Average (and standard deviation) of retention times for the standards and samples.
2. Calibration curves (graphs and equations) using peak height as your instrument signal metric.
3. Quantified concentrations of catechin and gallic acid for the green tea tablet, if possible.

*Pro Tip*

If you make a template to analyze the data, it will save time later. Check out the appendix of this lab manual for tips on how to make a data analysis template (Appendix B).

Your TA will ask you the following questions to make sure you understand the data:

1. Were the retention times reproducible (for the HPLC and uHPLC separately) across all the standards in the calibration curve and the sample? What evidence do you have to support that?
2. How did you construct the calibration curves and quantify the concentrations of catechin and gallic acid in the green tea tablet?
3. Do the quantified amount of each polyphenol match via the different methods? (No statistics necessary.) Share a compelling reason why this would (or wouldn’t) be the case.

**Analysis of Samples on the HPLC**

*This analysis will allow you a chance for hands-on instrument time with the HPLC. You will be allowed to move to this step once you have approval from your TA after proving you can analyze the data.*

1. Sign up for a day/timeslot to run on one of the HPLC units assigned to your section. You will be given a 30-minute timeslot to analyze 2 samples (4 injections total), that you should have prepared in advance:
   a. 1 positive control (with both catechin and gallic acid)
   b. Brewed tea sample
2. Other things you should plan for include:
   a. For your positive control, make sure to consider the calibration range of each chemical. You only need to collect 1 technical replicate/injection of your control.
   b. For your brewed tea sample, please put the tea in boiling water to ‘brew’ for 3 minutes prior to diluting to 100 mL (the standard volume for a cup of tea). Filter the tea prior to analyzing on HPLC. Dilute the tea further upon the results found in your pre-lab questions.
   c. You will need to rinse the syringe at least 3 times prior to an injection.
   d. You will want to plan to collect at least 3 technical replicates/injections of the brewed tea.
   e. Calibration curves for each HPLC (#1-#4) will be posted on Canvas for each day of the experiment.
   f. You can save the brewed tea and positive control sample from the previous step in the 4 degrees C fridge in well labeled 125 mL bottles to use for the uHPLC.

**Analysis of Samples on the uHPLC**

*This analysis will allow you a chance for hands-on instrument time with the uHPLC. You will be allowed to move to this step once you have analyzed (and worked up the data) from the HPLC.*

1. Sign up for a day/timeslot to be introduced to the uHPLC and to add your samples to the sample queue. You will sign up for a 10-minute timeslot to be introduced to the following items:
   a. The configuration of the uHPLC
   b. The steps that were used to prepare the uHPLC for your samples
   c. How the instrument will be set up to collect data
   d. How the data will be analyzed
2. You should prepare the following samples to be put in 1 mL vials and analyzed by the uHPLC:
   a. 1 positive control (with both catechin and gallic acid)
   b. Brewed tea samples
3. Other things you should plan for include:
   a. Name your samples appropriately using the following structure:
      i. SectionNumber_Date_GroupName_TeaType
      ii. (*e.g.,* 621_02292024_CoolCucumbers_Green)
      iii. Make sure you make a note with the course instructor how you diluted your original, brewed tea sample. You will be expected to submit your undiluted mg polyphenol/tea bag once the results are returned to you.
   b. While you will only have 1 injection of each, in your data processing, you will analyze all of the submitted tea samples from your section, considering them each as biological replicates. You only need to consider your positive control.
   c. Calibration curves (including a blank) for uHPLC will be posted on Canvas for each day of the experiment.
   d. You can use the brewed tea and positive control sample from the previous step if stored correctly.
   e. The results will not be provided instantaneously. Just like for the HPLC, the calibration curves (and blank) will be posted for you, and the sample results will be posted on Canvas once they are analyzed by the instrument and then processed by the course staff. Your lab report will not be due until the entire class has received their data. That due date you will be announced to the class on the LMS.

*Pro Tip*

uHPLC is more sensitive than HPLC; any contamination will be easily seen. Be careful to clean your glassware carefully, especially if you reuse it.

**RESULTS/CALCULATIONS**

*Fill out the answer sheet for this experiment completely. Attach copies of your notebook pages and submit them to the LMS. Make sure your notebook pages include the following:*

1. With the sample data (HPLC and uHPLC), work up the calibration curves, including the “y=mx+b” equation and the R^2^. Assess the linearity of these calibration curves. Are there any points you would remove or add? Justify that change. *Do not force your calibration curve through 0.*
2. For the standard sample data, were the retention times reproducible (for the HPLC and uHPLC separately) across all the standards in the calibration curve and the sample? What evidence do you have to support that?
3. For the sample data, can you (visually, no statistics necessary) say the amount of polyphenols matches what is found on the bottle, assuming the amount listed on the green tea tablet bottle is one type of polyphenol (**Figure 6**)? If not, what is a compelling reasoning for this difference?
4. What were the sticking points about analyzing the sample data? Write down reminders for yourself now just in case you use this technique in the future.
5. Using your own prepared, samples (NOT the supplied data)…
   a. What quantity of gallic acid and catechin were determined in your tea sample using both the uHPLC (using the class’s biological replicates) and HPLC (your technical replicates)? Include an average and standard deviation for both instruments.
      i. Statistically, how did these compare to the value you found in the literature?
   b. For both the HPLC and uHPLC data specifically, were the retention times of catechin and gallic acid the same for the positive control as for the individual calibration standards? If they are different, justify why this may be the case.
   c. For the uHPLC specifically, what differences between the fluorescence and absorbance measurements did you see? Were the chromatograms different? The same? Justify any potential changes you see.
6. If you were forced to pick one instrument and analysis configuration, which would you choose? Justify that choice.
7. Write a 200-word abstract in paragraph format that captures the following items (1-2 sentences for each) for the data YOU/the class collected (not the sample data):
   a. What scientific (or general) background is important for us to understand and care about these experiments?
   b. What are we trying to determine? What technique/instruments are we using to answer that question?
   c. What were the concentrations (± standard deviation) of catechin and gallic acid found in your type of tea sample (*i.e.,* green or black, making sure you mention which one)? Were they the same or different (statistically, *e.g.,* t-value comparison, *etc.*) from the literature? Make sure to report the statistics used to frame that statement.
   d. What (if any) differences were found between the HPLC and the uHPLC?
   e. What (if any) differences were found between the uHPLC’s absorbance and fluorescence detectors?
   f. Which instrument do you think is the best instrument for this application, and why?
   g. What future directions do you see yourself taking this experiment? Are there changes you would make, for example? Or samples you think would be best analyzed via this method?

**CHALLENGE QUESTIONS FOR YOUR LAB NOTEBOOK**

Challenge questions are designed to make you think deeper about the concepts you learned in this lab. There may be multiple answers to these questions! Any honest effort at answering the questions will be rewarded.

1. Whoops! Instead of the C18 column, you put a pure silica column into the uHPLC, which means that stationary phase is VERY polar.
   a. If we didn’t change the mobile phases, predict what the chromatogram would look like, including a drawing.
   b. If we changed the mobile phase to hexane and isopropyl alcohol, predict what the chromatogram would look like, including a drawing.

**REFERENCES**

(1)	El-Saadony, M. T.; Yang, T.; Saad, A. M.; Alkafaas, S. S.; Elkafas, S. S.; Eldeeb, G. S.; Mohammed, D. M.; Salem, H. M.; Korma, S. A.; Loutfy, S. A.; Alshahran, M. Y.; Ahmed, A. E.; Mosa, W. F. A.; Abd El-Mageed, T. A.; Ahmed, A. F.; Fahmy, M. A.; El-Tarabily, M. K.; Mahmoud, R. M.; AbuQamar, S. F.; El-Tarabily, K. A.; Lorenzo, J. M. Polyphenols: Chemistry, Bioavailability, Bioactivity, Nutritional Aspects and Human Health Benefits: A Review. *Int. J. Biol. Macromol.* **2024**, *277*, 134223. https://doi.org/10.1016/J.IJBIOMAC.2024.134223.

(2)	Câmara, J. S.; Albuquerque, B. R.; Aguiar, J.; Corrêa, R. C. G.; Gonçalves, J. L.; Granato, D.; Pereira, J. A. M.; Barros, L.; Ferreira, I. C. F. R. Food Bioactive Compounds and Emerging Techniques for Their Extraction: Polyphenols as a Case Study. *Foods 2021, Vol. 10, Page 37* **2020**, *10* (1), 37. https://doi.org/10.3390/FOODS10010037.

(3)	Anli, R. E.; Vural, N.; Kizile, E. An Alternative Method for the Determination of Some of the Antioxidant Phenolics in Varietal Turkish Red Wines. *Journal of the Institute of Brewing* **2008**, *114* (3), 239–245. https://doi.org/10.1002/J.2050-0416.2008.TB00334.X.

(4)	Harris, D. C. .; Lucy, C. A. . *Quantitative Chemical Analysis*; Macmillan Learning, 2020.

(5)	*12.4: Gas Chromatography - Chemistry LibreTexts*. https://chem.libretexts.org/Bookshelves/Analytical_Chemistry/Analytical_Chemistry_2.1_(Harvey)/12%3A_Chromatographic_and_Electrophoretic_Methods/12.04%3A_Gas_Chromatography (accessed 2026-04-29).

(6)	Ball, D. L.; Harris, W. E.; Habgood, H. W. Errors in Manual Integration Techniques for Chromatographic Peaks. *J. Chromatogr. Sci.* **1967**, *5* (12), 613–620. https://doi.org/10.1093/CHROMSCI/5.12.613.

(7)	Wangkarn, S.; Grudpan, K.; Khanongnuch, C.; Pattananandecha, T.; Apichai, S.; Saenjum, C. Development of HPLC Method for Catechins and Related Compounds Determination and Standardization in Miang (Traditional Lanna Fermented Tea Leaf in Northern Thailand). *Molecules 2021, Vol. 26, Page 6052* **2021**, *26* (19), 6052. https://doi.org/10.3390/MOLECULES26196052.

(8)	*Phenol*. https://omlc.org/spectra/PhotochemCAD/html/072.html (accessed 2026-04-29).

***APPENDIX A****: Unknown Peaks and Determination via Mass Spectrometry (This document was developed using data collected by Thao Duong, a graduate student in the University of Wisconsin-Madison Chemistry Department and Martha Vestling, Ph.D., the Director of the Mass Spectrometry Facility in the Chemistry Department.)*

During the lab^1^, you learn very quickly (or maybe even realized in advance) that real world samples are much more complex than our standards. Each of our standards produces 1 peak in the chromatogram (theoretically), although we do see some residual “gunk” at the end (~15 minutes) of our chromatograms (**Figure 1**), likely impurities in the standards, solvents, or column we are using (~98% pure, as provided by the chemicals/equipment’s companies). If you wish to see more information about how the purity of gallic acid is assayed by Chem-Impex International, ask the course staff! *(Fun Fact: They use a titration!)*

***Figure 1****: Representative uHPLC chromatograms of catechin (blue = 1 ppm; pink = 200 ppm) using the (a) UV-Vis absorbance detector or (b) fluorescence detector.*

When analyzing the green tea tablet or any of the steeped teas, we certainly see many other peaks in the chromatogram. Research in the literature supports more than gallic acid and catechin being present in the tea samples. In fact, the literature article we used in the lab as the basis for our chromatographic separation highlighted many species that were all derivatives of catechin and gallic acid (as well as caffeine) in their own tea sample (Camellia sinensis, the parent species that is derived to make white, yellow, green, oolong, dark, and black tea). Thus, it is natural to think that, qualitatively, the species found in this article will also be found in our sample!

As of right now, just looking at the chromatograms, we have no ability to confirm or deny the previous statement. Absorbance nor fluorescence will be specific enough to determine the differences between these similarly structured. Some of the only structural differences are isomeric, such as catechin and epicatechin, but it is quite possible that those differences do cause slight differences in the retention times of the molecules. Instead of absorbance/fluorescence, we must use another detector that can give us structural information that is detailed enough to distinguish the VERY similar structures above (or at least try). One common detector used after HPLC separation is mass spectrometry (MS), which can analyze the mass of a molecule. MS has really obtained public importance after the creators of 2 techniques, electrospray ionization (ESI) and matrix-assisted laser desorption/ionization (MALDI), won the 2002 Nobel Prize. Both of these techniques are considered “soft” and thus have allowed the analysis of proteins and other biological molecules by MS. Thus, in comparison to other techniques, like spectrophotometry, MS is incredibly young!

MS works by taking a molecule, ionizing (*i.e.,* charging it) it, and then measuring the resulting mass-to-charge (*m/z*) ratio to backtrack to the actual mass of the original molecule. The charge can be a single or multiple charges; it can also be either positive or negative. Both of these options depend upon the type of ionization that is occurring; examples include adding a H^+^ to removing an electron. Once charged and the initial *m/z* values have been analyzed, the instrument can select a single *m/z* (ideally a single species!), bombard it with gas to break it apart, and then analyze the fragments (which is called tandem MS, or MS/MS). Once analyzed by the MS, sophisticated software “deconvolutes” the mass and charges as well as can rebuild the fragmented species. (Remember that someone had to do this by HAND before computers or software to do this existed!) The overall process is described in **Figure 2**.

***Figure 2****: General process of MS analysis.*

The University of Wisconsin-Madison Chemistry Department has its own MS core facility (<https://massspec.chem.wisc.edu/>) that allows others in and out of the department to analyze samples either through being trained to use the instrument or submitting the sample. We prepared 3 samples to be analyzed: (1) green tea tablet; (2) brewed green tea; (3) brewed black tea. Dr. Vestling analyzed the 3 samples using MS following chromatographic separation. **Figure 3** shows the resulting chromatograms (and thus peaks that were selected for MS analysis). **Table 1** shows the instrument and HPLC parameters used by Dr. Vestling. **Table 2** shows the resulting identifications from MS/MS. Looking at these results, it isn’t possible, using MS alone, to determine which peak is the “epi” vs. the non-“epi” versions of these polyphenols. We would need to analyze standards and obtain the retention times (and ideally the resulting MS/MS spectra) to match to either the “epi” vs. the non-“epi” versions of these polyphenols.

***Figure 3****: Chromatograms obtained by the MS Center of each sample.*

***Table 1****. HPLC parameters for the analysis of the tea samples submitted to the MS Center.*

| Column | Agilent PLRP-S Column, 300 Å Pore Size, 3 μm Particle Size, 50 mm Length x 2.1 mm Internal Diameter |
| --- | --- |
| Mobile Phases | Solvent A 0.1% Formic Acid in Water  Solvent B 0.1% Formic Acid in Acetonitrile |
| Gradient Elution | 0-2 minutes 5% B, 2-7 minutes ramp to 25% B, 7-12 minutes hold at 25% B, 12-15 minutes ramp to 80% B, 15-16 minutes drop to 5% B, 16-20 minutes hold at 5% B |
| HPLC System | Thermo Fischer Dionex UltiMate 3000 System |
| Detector Systems | Bruker Impact II (*This purchase of this instrument was made possible by the Bender legacy gift to the Department of Chemistry.)* |
| Flow Rate | 0.300 mL/min |

***Table 2****. HPLC parameters for the analysis of the tea samples submitted to the MS Center.*

| **Retention Time (min)** | **m/z** | **Identified Species** |
| --- | --- | --- |
| 4.7-4.9 | 307.083 | (Epi-)catechin |
| 5.4-5.5 | 307.083 | (Epi-)catechin |
| 5.8-6.0 | 195.088 | Caffeine |
| 6.4-6.5 | 291.088 | (Epi-)gallocatechin |
| 7.3-7.4 | 459.095 | (Epi-)gallocatechin Gallate |
| 8.2-8.3 | 443.100 | (Epi-)catechin Gallate |

So, why don’t we analyze more than just gallic acid and catechin in our lab? Great question! Unfortunately, it all comes down to cost; these species are very hard to isolate, making getting pure standards (even just a milligram!) is thousands of dollars. For context, the catechin ((2R,3S)-2-(3,4-Dihydroxyphenyl)-3,4-dihydro-1(2H)-benzopyran-3,5,7-triol) we use in the lab is, currently, $152 for 10 grams (≥98% purity by HPLC), while epi-catechin ((2R,3R)-2-(3,4-Dihydroxyphenyl)-3,4-dihydro-1(2H)-benzopyran-3,5,7-triol) (≥98% purity by HPLC, from green tea) is $199 for 5 milligrams! Notice that the only difference is in the S or R in the 3^rd^ position, highlighted in red. We are not a research lab, and, thus, we have a much smaller budget. Instead, we try to expose students to potentially cutting-edge research within the limitations of our space and budget; thus, why we are only able to analyze gallic acid and catechin standards.

**REFERENCES**

1. “The Two Phases of Separation: Using Liquid Chromatography and Polarity to Analyze Polyphenols.” A Manual of Experiments for Analytical Chemistry. Department of Chemistry at UW- Madison, Spring 2024.

***APPENDIX B****: How to Build a Template to Analyze “Big Data” (This document was developed by Dr. Amanda Buchberger.)*

Replicate analysis is an important part of being an analytical chemist. Whether it is reproducibility (you doing the same experiment multiple times) or repeatability (others doing the same experiment), you may need to analyze almost the exact same data set multiple times…which can be tedious and boring! To make your life easier, if you need to analyze the data set multiple times, it is a good idea to develop a template spreadsheet that, when the newly collected data is entered, can calculate all the numbers and create all the graphs you would need! The template is then copied and modified for each individual replicate.

For many projects, it is a good idea to organize your template spreadsheet as follows:

- **The first tab is a cover sheet that organizes the relevant data.** This could be graphs or the most important numbers before or after analyzing the data. This could also be where you perform statistics on the information from multiple data sets.
- **Keep a blank version of the template that can be copied and then modified**. It helps to make sure you don’t delete anything super important by accident.
- **The following tabs are the copied (and filled out) template**. Label each tab appropriately with the replicate number or person that replicate came from, for example. If you could also organize by date, feature, or anything else that is relevant and makes it easy to find the right tab!

Good templates have the following features:

- **Cells that need to have experimentally collected data entered are visibly highlighted, such as coloring them.** Include a legend that articulates what is put into each colored cell.
- **Cells and graphs are formatted as detailed as possible (axes, significant figures, *etc.*) in the template before copying to enter data.** This includes any conditional formatting, text size or color, or calculations you want to be present. It can be frustrating if you need to modify one thing on 40 tabs!
- **Everything is labeled and organized so that another person can look at the template and know what is put in each cell.** These other people could be your project members, classmates, TA, or course instructor. One way to do this is to include column headers (and units as needed!).

Here is an example below (with a note of what this template needs and calculates):

- Values that should be entered include:
  - Time (of reaction, not retention time)
  - Peak Area (of each compound, 6 total)
  - Calibration curve information
- Values that are calculated include:
  - Concentration (and manipulations of concentrations for graphs)
  - Slope and R^2 of graphs
    i. The highest R^2 for each compound is conditionally formatted.
- Graphs that are made include:
  - 3 plots per compound (18 total)
