# Supplementary material for: Pick Your Own Adventure: Orthogonal Methods for Analyzing Polyphenols
Source: J Chem Educ. 2026 Jul 17;103(8):4243–54. doi: 10.1021/acs.jchemed.5c01459 (PMC13480120; doi:10.1021/acs.jchemed.5c01459)
Supplement: Supplementary file 9 [file ed5c01459_si_008.docx]

**Pick Your Own Adventure: Orthogonal Methods for Analyzing Polyphenols**

Amanda Rae Buchberger^1^*, Kristel M. Forlano^1^, Madeline J. Lund^1^, Pamela S. Doolittle^1^, Dominic M. Colosi^1^, Thao U. Duong^1^, Brian J. Esselman^1^, Kyoung-Shin Choi^1^, Robert J. Hamers^1^

^1^Department of Chemistry, University of Wisconsin-Madison, 1101 University Avenue, Madison, WI 53706

*Email Addresses:

[amanda.buchberger@wisc.edu](mailto:amanda.buchberger@wisc.edu)

# Lab Manual for Spectrophotometric Analysis of Polyphenols using Vanillin (VS) Lab Module

# SPECTROPHOTOMETRIC QUANTITATION OF POLYPHENOLS LEVERAGING VANILLIN CHEMISTRY

*In this lab, students will explore the reactivity, specificity, and sensitivity of vanillin (made with different solvents) for different polyphenol species. Once the reactivity of vanillin and a variety of polyphenols have been characterized, the calibration curve range(s) for quantitation will be defined and then applied to a real sample. Quality assurance metrics, including a spike recovery, detection limit estimate, and a positive control analysis will confirm that the method developed is appropriate for the sample of interest.*

Visual Abstract:

To cite this lab manual: “Spectrophotometric Quantitation of Polyphenols Leveraging Vanillin Chemistry.” A Manual of Experiments for Analytical Chemistry. Department of Chemistry at UW- Madison, Spring 2024.

*Did You Know?*

Keep an eye out for these green boxes throughout your lab manuals! They will contain little tidbits connecting the experiments you’re completing with real research and industrial applications, provided by Dr. Bradley Bolling in the Department of Food Science at UW-Madison and Dan Berg, an Analytical Services Manager at Eurofins Food Testing Chemistry in here in Madison!

BACKGROUND

Just like there are many tools in a toolbox, the analytical chemist has a plethora of chemistries, techniques, and instrumentation to identify and quantify analytes of interest. Qualitatively, we must first discern WHAT needs to be measured, and knowing that, can select and characterize an appropriate method for analysis. In this lab series, we will explore a variety of techniques used to quantify polyphenols. This manual focuses on spectrophotometric methods for quantifying polyphenols.

Importance

Generally, polyphenols are a large family of molecules ([>8,000 identified](https://www.chhs.colostate.edu/krnc/monthly-blog/what-are-polyphenols-another-great-reason-to-eat-fruits-and-veggies/)) that (as you guessed) contain many phenol (C_6_H_5_-OH) groups (which is the main structural feature that most spectrophotometric assays take advantage of!).^1^ They are incredibly diverse, ranging in size from ~100 to ~800 Daltons (~g/mol). There are different classes (and structural motifs). Generally, non-flavonoids contain only one phenol ring, and flavonoids contain two phenol rings. Polyphenols are naturally found in many fruits, vegetables, and teas. Interestingly, the pulp of fruits/vegetables is rich in non-flavonoid compounds, while the skins, seeds, and stems are rich in flavonoid compounds. Green tea has gained a lot of attention for its high concentrations of the polyphenol epigallocatechin gallate (EGCG).^2^ Easily purchasable and simple polyphenols that you will work with in this lab are catechin (a flavanol) and gallic acid (a non-flavonoid, phenolic acid). The great variety in structures makes analyzing these molecules difficult without customizing or restricting the exact samples analyzed.

On a more microscopic scale, polyphenols are known primarily as antioxidants, meaning they inhibit oxidation. Uncontrolled oxidation inside the human body (*e.g.,* of DNA, proteins) results in cellular damage that can lead to downstream problems. Antioxidants react with these oxidizing species (*e.g.,* oxygen) to reduce their oxidative behavior and therefore reduce cellular oxidation in the body. Many people associate consuming antioxidants with reducing the effects of aging and mitigating risks of certain cancers and other diseases.

Building a Method (The Important Stuff)

The literature is a great place to find inspiration and pre-established methods. In the realm of spectrophotometry, two methods are very popular for the analysis of polyphenols: (a) the Folin-Ciocalteu (FC) assay and (b) the vanillin assay. When comparing these 2 methods, they have a similar mechanism (*i.e.,* a specific reagent reacts with a polyphenol molecule to create a color that correlates to concentration using the Beer-Lambert Law). How do we choose? Begin by considering the 3S’s: specificity, sensitivity, and “spendiness” (*i.e.,* cost). In all 3 categories, the vanillin assay is the most attractive, depending upon what molecules you want to quantify. In full clarity, the assay is based upon reducing phenolic hydroxyl groups (*i.e.,* phenols, which all polyphenols have), but vanillin mainly has publications focusing on flavanols and other related molecules.^3^

*Did You Know?*

The FC assay is the most common method used in industry for measuring polyphenol content. The FC method is used because it is very generic, easy, and consistent. The vanillin assay, on the other hand, is more sensitive to flavan-3-ols (which include catechin).

Before we jump into building the method, it is important to understand the *chemistry* behind the assay. Vanillin isn’t a large molecule, especially compared to many polyphenols. The reaction between vanillin and a condensed tannin is illustrated in Figure 1.^4^ As you can see, the structure of the polyphenol can have a huge impact on how much vanillin is needed (see red circle for second possible reactive site for this polyphenol (a condensed tannin)) and if vanillin can react at all. The stoichiometry of the reaction appears to be 1:1 from the figure, but the stoichiometry will vary depending upon the number of phenolic groups present (and their positions on the aromatic ring). The H^+^ (also known as H_3_O^+^) added acts as a catalyst, thus keeping an acidic environment is key for this reaction to work quickly and efficiently.^5^

*Figure 1: Reaction of vanillin with a (tannin) polyphenol to create a colored solution that can be quantified via spectrophotometry. ^Modified from 4 with permission^ The H^+^ acts a catalyst to react vanillin with the polyphenol at a variable stoichiometric ratio dependent upon the structure of the polyphenol.*

Next, we need to find the BEST (for us) paper to perform the vanillin method. The method was first developed in the 1960s, and there exist several “flavors” of the experiment in the literature. When reading a paper, it is good to start with the “methods” section to make sure they are even feasible in our lab spaces. One of the simplest methods is described in a paper by Kalidass *et. al.*.^6^ On Page 137, it shows their methodology (Figure 2). This (oversimplified) procedure seems to be:

1. Make vanillin (and make it cold).

*Figure 2: Excerpt from paper of interest describing the Vanillin assay.^6^ Reproduced with permission under CC BY 4.0.*

2. Prepare polyphenol sources.
3. Mix together X mL of polyphenol source & 6.5 mL of vanillin.
4. Dilute to 10 mL.
5. Let color develop for 15 minutes.
6. Collect absorbance at 500 nm (for both calibration curve and sample of interest).

Let’s start with Step 1. Vanillin exists in your kitchen cupboard as artificial vanilla (smells great!).^7^ The paper has us dissolve solid vanillin fully in 70% sulfuric acid. Based upon other publications, the sensitivity of the assay is based upon the pH of the solution, meaning the more acidic the solution, the more sensitive the assay.^3^ Interestingly, the use of sulfuric acid is better than HCl!^3^ 70% sulfuric acid is ~13 M….seems acidic enough to me!

Next (Step 2), preparing polyphenol sources is complex. Let’s start talking about the standards first. A calibration curve is a scientific tool that allows us to correlate concentration to a specific signal from an instrument and, in our case, that will be absorbance (see Figure 3). Calibration curves and how they are used depend upon the relationship between those 2 variables. In our case, absorbance and concentration are connected via the Beer-Lambert Law, also known as Beer’s Law.^8 (Chapter 18)^

$\boldsymbol{A}_{\boldsymbol{\lambda}}\boldsymbol{=}\boldsymbol{\epsilon}_{\boldsymbol{\lambda}}\boldsymbol{bc}$	(1)

A is absorbance. ε is absorptivity of the molecule at a specific wavelength, which creates a wavelength-specific response for the absorbance. b is the pathlength of the instrument. c is the concentration of the molecule of interest. Typically, the wavelength for analysis is chosen for a few reasons:

1. because the molecule has a high molar absorptivity at that wavelength,
2. no or very little interferences occur at the chosen wavelength, and
3. the instrument source and detector work well for the wavelength choice.

When a reaction occurs, methods will use the wavelength of maximum absorbance, or λ_max_, for the wavelength of analysis, as it produces the most sensitive assay. Beer’s Law assumes a linear relationship between the absorbance and concentration, thus, our calibration will have a linear equation overlayed onto its points using a “y=mx+b” equation, where the slope (m) is equivalent to εb. There exists limits of linearity on the higher (see Point 3 in Figure 3 (left)) and lower (see Point 1 in Figure 3 (left)) ends of the calibration curve; in other words, the signal stops increasing or decreasing proportionally to the concentration, respectively. Beer’s Law isn’t valid outside the linear range (see Region 4 in Figure 3 (left)) (which is ~0.1-1 absorbance units); thus, method development is necessary to ensure you’re working inside the linear range of the assay. Please note that you cannot report a concentration above or below your determined calibration curve; instead, you would say “above/below the upper/lower limit”, respectively. As a starting point, the authors describe using standard samples that were between 5 mg/L and 25 mg/L. We cannot guarantee that range will work for us since we have different instrumentation in our labs. But it is a start! You should confirm linearity BOTH visually and using a statistical metric, like R^2^. *If you want to see an interesting case study about calibration curves. Check out Appendix B.*

*Figure 3: Example calibration curve used for Beer’s Law showing the full dynamic range (left) and the linear range (right). 1 = Limit of Detection. 2 = Noise. 3 = Limit of Linearity. 4 = Linear Range. 5 = Dynamic Range.*

Spectrophotometers do not actually measure absorbance directly. Instead, it measures the percentage of light transmitted (also known as percent transmittance) at a specific wavelength. This is valuable to us, as the instrument naturally outputs more significant figures when measuring percent transmittance compared to absorbance. Thus, once we confirm the λ_max_ (the wavelength with the highest ε), which the paper states is ~500 nm for catechin, we can collect single values for each solution rather than several hundred data points (*i.e.,* a full wavelength spectrum). The conversion between transmittance and absorbance is:

$\boldsymbol{A=-log T}$	(2)

Next, while we know the concentrations of our standard samples, to analyze a real-world sample, a lot of choices must be made. What concentration are we going to assume our sample is at? What are we going to do to prepare samples within the calibration range? This is especially hard because “polyphenols” include a wide variety of molecules! If a reported concentration for a sample is 25 mg of polyphenols, which one(s) could it be?! Whatever choices you make, it is important to note that method development involves many iterations of trial and error, and it’s okay to change your procedure on the fly if things aren’t working as you expect. In the end, your goal should be to get your sample in the MIDDLE of your calibration curve (to reduce experimental error, see Figure 3) and make sure you report appropriate units. You will notice the authors of the paper report in catechin equivalents since they used a catechin calibration curve. If you build your calibration curve using gallic acid, for example, you should report in gallic acid equivalents.

Adapting a Method (Challenges and Nuances)

While the method seems simple, there are a few things to pay attention to. For example, why is it that vanilla extract we have at home is dissolved in ethanol? Great question! The solubility of vanillin in water isn’t well defined^7^, but it’s clear the solubility is MUCH higher in ethanol. Depending upon the source cited^6^, a 1% vanillin aqueous solution is at the solubility limit. If we add ethanol to help the vanillin dissolve, does it make a difference in vanillin assay chemistry? Does it make the assay better? Worse? That is something for you to explore!

On top of that, most of the papers published only use catechin as their standard. Why? Gallic acid is so much cheaper than catechin (and also likely more stable!). Could it work as a standard? One paper says it doesn’t work^3^, but, it is worth testing for the cost difference! For context, 100 grams of gallic acid is ~$100, compared to ~$100 for 10 grams of a (+)-catechin hydrate.

To have confidence in our method, we need to do quality assurance to make sure it is working properly. Firstly, make sure you are using the right blank. The blank should account for everything but the sample of interest. That means that, the vanillin solution you use/make will change the blank composition. Beyond the linear range, it is a good practice to know the detection limit of the assay, as it is quite possible that we will work with samples that do not contain any polyphenols, and, we need to ensure the signal we are measuring is “real” versus not. The limit of detection is usually lower than the limit of linearity (see Point 1 in Figure 3 (left)). The detection limit (also known as the method detect limit (MDL) or limit of detection (LOD)), is the smallest quantity (*i.e.,* concentration) of an analyte “significantly different” from the blank. Anything below the detection limit we report as either “0” or “below the detection limit”; although we cannot technically confirm the absence of the analyte (since below the detection limit does not mean not present), reporting “0” is useful when trying to perform statistics.^8 (Chapter 4)^ The LOD is defined by that “wiggle” in the spectrum or the baseline noise (highlighted in Figure 4 and Figure 3-Region 2 (left)). That systematic error is captured in our calibration curve as the b-term, which is also why we do not want to force our calibration curve through 0.

Instead of going through an in-depth procedure like the Environmental Protection Agency’s method^9^, an estimate is ‘good enough’ in many cases. By definition, the detection limit is at a signal-to-noise ratio of ~2.5-5 for an assay. If we know what the signal to noise is for X concentration (Figure 4), we should be able to proportionally calculate the concentration at the estimated detection limit signal-to-noise range (Equation 3).^7^

$\frac{{S/N}_{1}}{{Concentration}_{1}}=\frac{{S/N}_{2}}{{Concentration}_{2}}$	(3)

To know that the method can accurately measure the analyte of interest, use a positive control, meaning create a solution of approximately the same concentration of a standard as you would expect of that molecule in your sample *to make sure you measure that expected amount*. (If you make a solution that is 10 mg/L and it measures not 10 mg/L (*e.g.,* 8 mg/L), something is likely wrong with your assay or how you made solutions!) Before you move onto another step, you should stop and make sure any differences are not due to human error by repeating the measurement.

*Figure 4: Example spectrum showing how signal and noise are estimated.*

Next, you should perform a spike recovery (meaning adding a known amount of the analyte of interest to your sample and seeing if we measure that expected concentration increase) to confirm that the *matrix* doesn’t impact the accuracy of our measurements.^8 (Chapter 5)^ To determine the percent recovery of a spike, the sample is split into two portions and a known amount of a standard solution of analyte is added to one portion. The concentration of the analyte is determined for both the spiked and un-spiked portions. The percent recovery is calculated as:

$\% recovery=\frac{C_{spiked sample}-C_{unspiked sample}}{C_{added}}*100\%$	(4)

where *C* stands for concentration. A generic workflow for spike recovery is shared in Figure 5. Note that the spike can be added before the dilution to the mark on the volumetric flask, making your life a little easier! To perform a spike recovery, practical criteria you will want to consider when designing your spike include:

(a) add an amount of standard to the sample that increases the concentration of your sample to still be within the calibration curve, considering the dilution effects.
(b) add a small amount of volume with your spike so that the matrix of the sample is mainly preserved.

*Figure 5: Example workflow for performing a spike recovery. Each volumetric flask is 100.0 mL.*

Another (smaller) note is that the linear range, calibration curve metrics (*e.g.,* slope), method detection limit, *etc.* are all specific to an instrument. Meaning, due to manufacturing differences, age, or cleanliness, even different versions of the exact same instrument could have slightly different measured values. Does that mean we cannot trust any of our values from the previous day? Absolutely we can, but, it is a good idea to confirm values or accept that we are making an assumption about the validity or accuracy of our data.

*Did You Know?*

Dietary supplements are the most commonly analyzed product at Eurofins. Supplements are tested on a regular basis, sometimes even batch to batch. Food products don’t need to be tested as often. “A blueberry is a blueberry!”

- *Dan Berg*

Finally, preparing real samples is messy…the sample you are going to be working with is a green tea powder tablet that can be commercially purchased from a local chain drugstore (Figure 6). Interestingly, these “pills” are just gelatin capsules that can be popped open, and they contain a loose powder, appearing to just be green tea leaves crushed up. This is helpful for us, because gelatin could make our solutions thick, and, instead, we can just take the powder and process that. Next, according to the label, each tablet is standardized to contain a promised amount of polyphenols. That is a bit complicated for us, because we are not even sure what the exact polyphenols are…remember we can make an estimate and change later if needed. Luckily, we can make an educated choice in assuming that most if not all the polyphenol species in the green tea tablets are catechin or catechin-like species due to online sources.^2^

*Figure 6: Relevant photos of green tea tablets, the real sample for this lab. (a) Front of bottle. (b) Nutrition facts on back of bottle. (c) Pill, appearing to be powder in a gelatin casing.*

Safety Considerations

Whenever you develop a method, you want to make sure it can be done safely while minimizing other issues. We can look up the [Safety Data Sheet (SDS)](https://chemicalsafety.com/sds-search/) to find more information on these chemicals, especially if you haven’t worked with those chemicals before. In particular, we are working with *concentrated* sulfuric acid. Make sure you look into how to handle this safely.

Deliverables

The overall project lab has the following deliverables:

1. Recognize and compare/contrast authentic, modern analytical methods used to analyze polyphenols.
2. Construct and justify a method to analyze and statistically test a hypothesis focused on sample of personal interest.
3. Analyze and validate results with appropriate statistical methods and quality assurance and control metrics, including detection limits, controls, and the percent recovery of a spike.
4. Summarize and communicate findings in an oral presentation and written abstract.

This lab is framing your project lab experience and ability to complete the project lab deliverables. The specific deliverables of this lab are:

1. Adapt a previously published analytical method for 2 standard polyphenols (catechin and gallic acid) to our laboratory setting.
2. Create a coherent procedure by exploring undefined procedural steps and connecting appropriate conceptual knowledge.
3. Construct calibration curves and determine a method’s detection limit under a variety of different conditions to probe the linear range and sensitivity of a method.
4. Assess the validity of the quantitative results for a provided, commercial sample via a spike recovery and positive control.

**PRELABORATORY EXERCISES**

*Since the paper just lists concentrations and volumes, we will use the pre-lab preparation to create detailed instructions for making the solutions.*

1. Come up with a timetable to be able to complete the lab below. What checklist items should be completed each day? Note that you will have 40-50 mg of both catechin and gallic acid supplied as well as 500 mL of 70% H_2_SO_4_ to you TOTAL for all days for your group. Make sure to include in your plan if you are/are not going to make a new calibration curve each day. (HINT: You should!!!)
2. Determine how to make two 1% vanillin solutions: (1) made by dissolving vanillin in only 70% H_2_SO_4_ and (2) made by dissolving in vanillin in 10:90 ratio mixture of ethanol (EtOH) and 70% H_2_SO_4_. Assume you are going to make 100 mL for the first day (which can be scaled later, if needed). Remember that 70% H_2_SO_4_ will be supplied to you.
3. If we assume that all of the polyphenols found in the tablet are purely one polyphenol (*e.g.,* catechin or gallic acid), determine an initial plan for preparing the tablet. *Think about how you will create a solution from the tablet contents so that you can make a quantitative measurement of the contents. To make that measurement, you will react with the 1% vanillin to form the colored product.* (HINT: Read the label in **Figure 6**.)
4. Use the table below to determine a plan for making standards that range between 30 mg/L and 1 mg/L. You will make ~100 mg/L catechin and gallic acid solutions.
5. Put together a basic plan for how you prepare samples to perform a spike recovery. Be as specific on the volumes as possible on your prepared green tea sample.

*Pro Tip*

This plan will likely change based upon your final calibration curve or experiences with the tablet, but having an initial plan is important!

| *Final Concentration of Polyphenol Standard (mg/L)* | 1.0 | 2.5 | 5 | 10 | 15 | 20 | 25 | 30 |
| --- | --- | --- | --- | --- | --- | --- | --- | --- |
| Polyphenol Standard Added (mL) |  |  |  |  |  |  |  |  |
| Vanillin Added (mL) |  |  |  |  |  |  |  |  |
| Final Volume Diluted to (mL) |  |  |  |  |  |  |  |  |

**BEFORE YOU TAKE THE QUIZ**

*The point of the pre-lab quiz is to make sure you are fully prepared for the experiment and understand the major concepts and calculations. Before taking the quiz, please review the posted equations and understand how to use them.*

**EXPERIMENTAL**

USE CAUTION!

- ***The sulfuric acid used in this experiment will burn you badly. Be attentive to adhering drops on the glassware. Remove sulfuric acid with a lot of tap water. Don’t*** ***drink it. In case of eye contact wash the eyes with flowing tap water for 15 minutes, then go to the doctor.***

*Pro Tips*

- All solutions should be made fresh daily to avoid degradation of the reacting species via light and oxidation. For these solutions, a change in color as they are sitting for the day is not concerning.
- You will be given a set amount of materials on the first lab period. You will not get more, so you need to think about how to plan those amounts BEFORE doing experiment. DO NOT GO INTO LAB UNTIL YOU HAVE A PLAN.

**Key Solutions (as a Starting Point)**

*These are designed to get you started on the first experimental step. Feel free to update or change these depending upon your method development goes or what you need that day!*

1. Vanillin Solution (1%): Make these solution(s) using the plan developed in your pre-lab questions.
2. Polyphenol Standard #1 (Catechin, 100 mg/L): Weight out 0.01 grams of catechin and dissolve in 15 mL of ethanol prior to diluting to 100.0 mL with DI water.
3. Polyphenol Standard #2 (Gallic Acid, 100 mg/L): Weight out 0.01 grams of gallic acid and dilute to 100.0 mL with DI water.

*Pro Tip*

Talk to the stockroom if you want to use other sized glassware than what is suggested!

**Exploring Vanillin Solubility & Polyphenol Reactiveness with Vanillin & Determining λ_max_**

*This exploration will reveal the solubility of vanillin in an aqueous environment. The experiment will also determine whether the controls we've designed will react to perform a colored product we can then measure.*

1. Create the following solutions, following the procedure outlined in the paper:
   a. 6.5 mL 70% H_2_SO_4_ Vanillin, diluted to 10.00 mL with DI water
   b. 1 mL of Catechin + 6.5 mL 70% H_2_SO_4_ Vanillin, diluted to 10.00 mL with DI water
   c. 3 mL of Catechin + 6.5 mL 70% H_2_SO_4_ Vanillin, diluted to 10.00 mL with DI water
   d. 1 mL of Gallic Acid + 6.5 mL 70% H_2_SO_4_ Vanillin, diluted to 10.00 mL with DI water
   e. 3 mL of Gallic Acid + 6.5 mL 70% H_2_SO_4_ Vanillin, diluted to 10.00 mL with DI water
   f. 6.5 mL 10:90 EtOH:70% H_2_SO_4_ Vanillin, diluted to 10.00 mL with DI water
   g. 1 mL of Catechin + 6.5 mL 10:90 EtOH:70% H_2_SO_4_ Vanillin, diluted to 10.00 mL with DI water
   h. 3 mL of Catechin + 6.5 mL 10:90 EtOH:70% H_2_SO_4_ Vanillin, diluted to 10.00 mL with DI water
   i. 1 mL of Gallic Acid + 6.5 mL 10:90 EtOH:70% H_2_SO_4_ Vanillin, diluted to 10.00 mL with DI water
   j. 3 mL of Gallic Acid + 6.5 mL 10:90 EtOH:70% H_2_SO_4_ Vanillin, diluted to 10.00 mL with DI water
2. Collect a full spectrum of each sample. You only have enough solution to rinse the cuvette and analyze the sample ONCE (unless you remake it). You will want to blank the instrument with DI water, collect a spectrum of that blank, and then collect the spectra of the other related samples.

**Defining our Calibration Curve Range**

*This experiment, after defining (a) which polyphenols we can analyze, (b) which vanillin solution is appropriate, and (c) the λ_max_ of that vanillin complex(es) of interest, you will define the working range of your calibration curve.*

1. Build the calibration curve for the polyphenol standard(s) that worked in the previous mini-experiment as you planned in your pre-lab question. Analyze it in percent transmittance at the maximum wavelength that you determined in the previous mini-experiment. Determine the linear range. *You may need to add more points than originally planned to fill in the calibration curve with a minimum of 5 points within a linear range, not including your blank. Your blank does not count as a point on your calibration curve and should not be included.*
   a. Make sure you blank with a REAGENT blank, not just DI like above.

**Analysis of Real Sample**

*This experiment will be the application of the methods you developed in the previous mini-experiments.*

1. Obtain a green tea tablet from the stockroom or your TA. Using your notes from the pre-lab question and results from the calibration curve mini-experiment, prepare the contents of the tablet to be within the middle of the linear range of your calibration curve. Analyze it in percent transmittance at the maximum wavelength that you determined in the previous mini-experiment.

**Method Validation**

*This exploration will define the confidence you have in the concentration of your real sample previously analyzed.*

1. Collect a full spectrum of your highest calibration standard in the linear range. Determine the signal at λ_max_ and the noise at the baseline. You will use these values to estimate the detection limit.

*You will need to do some calculations after working with your real sample before completing the next steps.*

2. Using you pre-lab work as a starting point, perform a spike recovery of the polyphenols that you were able to measure using the vanillin assay. It is recommended that you add a spike that is ~1-2% (volume/volume) of the solution to avoid changing the matrix of the sample.
3. Create a positive control solution that is approximately the same concentration you planned to prepare your real sample, as determined in lab. See if you can replicate the matrix of your sample. Analyze that solution.

**RESULTS/CALCULATIONS**

*Fill out the answer sheet for this experiment completely. Attach copies of your notebook pages and submit them to the LMS. Make sure your notebook pages include the following:*

1. Justify which vanillin solution you decided to use and why.
2. Justify which polyphenol(s) you were/weren’t able to analyze using vanillin and why.
3. State the maximum wavelength for each polyphenol reacted with vanillin, making sure to include the plotted spectra as part of your submission.
4. Where did you determine the approximate upper limit of your calibration curve to be? The approximate lower limit? Include a copy of your final calibration curve(s). *Do not force your calibration curve through 0.*
5. How many milligrams of the polyphenol(s) were found in a single green tea tablet?
6. Determine the concentration of your positive control; was your assay able to accurately measure your polyphenol(s)?
7. Estimate the detection limit using the highest standard’s (from your linear range) signal-to-noise ratio.
8. Determine the spike recovery of your tea sample.
9. Write a 200-word abstract (in paragraph form) that captures the following items (1-2 sentences for each):
   a. What scientific (or general) background is important for us to understand and care about these experiments?
   b. What are we trying to determine? What technique are we using to answer that question?
   c. What polyphenols are able to be analyzed by the vanillin assay? Does the solvent make a difference?
   d. What range of concentrations were you able to analyze for the polyphenol(s), and at what wavelength? What is the detection limit we can measure accurately at? (Don’t forget units!)
   e. What amount of which polyphenols were found in a single green tea tablet? Was the method able to measure the concentration of your sample accurately? Did the matrix of the tea tablet impact the concentration we measured?
   f. What future directions do you see yourself taking this experiment? Are there changes you would make, for example? Or samples you think would be best analyzed via this method?

**CHALLENGE QUESTIONS FOR YOUR LAB NOTEBOOK**

Challenge questions are designed to make you think deeper about the concepts you learned in this lab. There may be multiple answers to these questions! Any honest effort at answering the questions will be rewarded.

1. One thing this lab is missing is replicates. There are 2 different types of replicates: (a) technical replicates and (b) “biological” replicates. Technical replicates are, as you anticipate, just the sample analyzed over and over again. This makes sure that the instrument is technically working well and is giving us the same answer (within an acceptable margin based upon uncertainty propagation) each time. But, this sort of replication gives us the variation in the measurement, not the actual sample. Instead, you would ideally measure “biological” replicates, which is another sample put through the exact same process separately, just like a titration! If we were to incorporate more “biological” replicates, describe how you would modify this procedure and estimate the amount of time you would need to complete that experiment. Also, mention any limitations of your plan.
2. [The Food & Drug Administration (FDA) does not approve supplements before they come to market.](https://www.fda.gov/consumers/consumer-updates/fda-101-dietary-supplements) Thus, it is on the company to make sure their claims are correct. The FDA instead reviews supplements as needed, especially if they are found to be unsafe. Based upon your results today:
   a. Would you say that the green tea tablet company is being truthful about their claims? Use statistical (meaning test if the 2 values/populations are the same using statistics) backing to confirm your claims.
   b. What is one suggestion you would give the company to make their labeling more accurate and/or precise?
3. Propose one reason for the difference in vanillin reactivity with gallic acid and catechin. The structures of each are provided below for your convenience. *Propose an answer to this question, and then refer to Appendix A included at the end of this lab manual.*

|  |  |  |
| --- | --- | --- |
| vanillin | gallic acid | (+)-catechin |

**REFERENCES**

(1)	El-Saadony, M. T.; Yang, T.; Saad, A. M.; Alkafaas, S. S.; Elkafas, S. S.; Eldeeb, G. S.; Mohammed, D. M.; Salem, H. M.; Korma, S. A.; Loutfy, S. A.; Alshahran, M. Y.; Ahmed, A. E.; Mosa, W. F. A.; Abd El-Mageed, T. A.; Ahmed, A. F.; Fahmy, M. A.; El-Tarabily, M. K.; Mahmoud, R. M.; AbuQamar, S. F.; El-Tarabily, K. A.; Lorenzo, J. M. Polyphenols: Chemistry, Bioavailability, Bioactivity, Nutritional Aspects and Human Health Benefits: A Review. *Int. J. Biol. Macromol.* **2024**, *277*, 134223. https://doi.org/10.1016/J.IJBIOMAC.2024.134223.

(2)	Câmara, J. S.; Albuquerque, B. R.; Aguiar, J.; Corrêa, R. C. G.; Gonçalves, J. L.; Granato, D.; Pereira, J. A. M.; Barros, L.; Ferreira, I. C. F. R. Food Bioactive Compounds and Emerging Techniques for Their Extraction: Polyphenols as a Case Study. *Foods 2021, Vol. 10, Page 37* **2020**, *10* (1), 37. https://doi.org/10.3390/FOODS10010037.

(3)	Sun, B.; Ricardo-da-Silva, J. M.; Spranger, I. Critical Factors of Vanillin Assay for Catechins and Proanthocyanidins. *J. Agric. Food Chem.* **1998**, *46* (10), 4267–4274. https://doi.org/10.1021/JF980366J.

(4)	Schofield, P.; Mbugua, D. M.; Pell, A. N. Analysis of Condensed Tannins: A Review. *Animal Feed Science and Technology*. May 16, 2001, pp 21–40. https://doi.org/10.1016/S0377-8401(01)00228-0.

(5)	Cucciniello, R.; Tomasini, M.; Russo, A.; Falivene, L.; Gambuti, A.; Forino, M. Experimental and Theoretical Studies on the Acetaldehyde Reaction with (+)-Catechin. *Food Chem.* **2023**, *426*, 136556. https://doi.org/10.1016/J.FOODCHEM.2023.136556.

(6)	Kalidass, S.; Daiyarvijaya, K.; Kumar, R. R. Comparative Study on Quantification of Total Catechins Using UV-Vis Spectrophotometric Method and High Performance Liquid Chromatography Techniques. *Oriental Journal Of Chemistry* **2021**, *37* (1), 136–142. https://doi.org/10.13005/ojc/370118.

(7)	*Vanillin | C8H8O3 | CID 1183 - PubChem*. https://pubchem.ncbi.nlm.nih.gov/compound/Vanillin (accessed 2026-04-27).

(8)	Harris, D. C. .; Lucy, C. A. . *Quantitative Chemical Analysis*; Macmillan Learning, 2020.

(9)	*Procedures for Detection and Quantitation - Documents | US EPA*. https://www.epa.gov/cwa-methods/procedures-detection-and-quantitation-documents (accessed 2026-04-29).

***APPENDIX A****: A Structural Explanation of the Chemistry Happening Between Vanillin, Catechin, and Gallic Acid*

*(This document was developed under the direction of Dr. Brian Esselman, Associate Organic Lab Director in UW-Madison Chemistry Department.)*

In the lab manual^1^, one challenge question asks you to do the following:

*Propose one reason for the difference in vanillin reactivity with gallic acid and catechin. The structures of*

*each are provided below for your convenience.*

|  |  |  |
| --- | --- | --- |
| vanillin | gallic acid | (+)-catechin |

In the background information, we share, generally, where we expect the vanillin and (+)-catechin to react (see **Figure 1** in the lab manual)^1^, but WHY is the real question? Using computational chemistry and concepts from CHEM 344/345, Dr. Esselman has developed the following explanation:

The vanillin is protonated in the acidic reaction conditions, which is a common way of activating carbonyl containing compounds to make them more reactive (more acidic and more electrophilic at the carbonyl carbon atom. The electrophilicity of the carbon atom in protonated vanillin results in a large lobe of its acceptor orbital, its lowest energy unoccupied orbital (LUMO), on the reactive carbon atom **Figure A1**. This makes this carbon the most reactive with negatively charged particles or nucleophiles.

***Figure A1:*** *LUMO Protonated vanillin with large lobe circled in blue.*

(+)-Catechin contains an electron-rich aromatic ring, due to its three oxygen atom substituents. Each of these oxygen atoms has a p-orbital lone pair that conjugates to the aromatic ring π system (**Figure A2**).

|  |  |  |
| --- | --- | --- |
|  |   −0.38  −0.38 | |
|  | | |

***Figure A2****: Oxygen-atom p-orbitals available to conjugate with the aromatic p system of (+)-catechin. The resulting NPA charges of the nucleophilic carbons are shown along with the resonance structures used to rationalize them.*

As a result of the buildup of negative charge via π conjugation, two carbon atoms in (+)-catechin are sufficiently electron-rich that they can serve as nucleophiles and react with the activated vanillin (electrophile). One of these possible reactions is shown in **Scheme A1**. Though not shown, it is likely that some portion of the molecules are reacting at the other nucleophilic carbon atom of (+)-catechin, designated with a pink question mark, and thus have is able to donate electrons from its electron-rich carbon atom to the activated carbonyl carbon forming a new σ_C–C_ bond.

Scheme A1: Activation of vanillin and EAS attachment of (+)-catechin

**Scheme A1** depicts the first step of an electrophilic aromatic substitution (EAS) reaction, which is reasonably favorable due to the activation of the nucleophile from three oxygen atom. Each oxygen atom is an *ortho*/*para*, activating group for EAS reactions. In the case of (+)-catechin, all three oxygen atoms cooperate to build up electron-density at those two carbon atoms. The rate-determining step of EAS reactions is the formation of an arenium cation. Due to the presence of three oxygen atoms the cationic charge of the resulting arenium cation is highly delocalized as depicted by the resonance structures in **Scheme A2**.

***Scheme A2:*** *Important resonance structures of a potential arenium cation generated in this reaction.*

After loss of an H^+^ from the arenium intermediate, the EAS reaction is complete. The base in this reaction is likely to be water, due to the aqueous reaction conditions. These reactions are nearly always kinetically controlled (irreversible reactions). As a result, the product formation and relative amount of product generated by attachment at each nucleophilic carbon atom are governed by the relative stabilities of each potential arenium cation.

Importantly, the EAS product is not compatible with the acidic reaction conditions and reacts further by a unimolecular elimination (E1) loss of water. All of the O-atoms in the molecule can and do act as bases with the acidic environment. They will form water leaving groups through protonation from the acid catalyst, best represented as H_3_O^+^. The protonated alcohol on the carbon atom between the two aromatic rings is most able to dissociate generating a carbocation. Both of the aromatic rings can stabilize the carbocation by delocalizing the positive charge throughout both aromatic rings. All of the oxygen atoms on each ring are positioned to be able to donate electron-density to the rings and further delocalize the positive charge. The extended conjugated π system, enhances the rate of water dissociation, which is the rate-limiting step for the E1 reaction.

*Scheme 3. Complete reaction of vanillin and (+)-catechin*

Once the benzyl cation forms, there is no β-hydrogen atom to be deprotonated. The benzyl cation, however, can form a *para*-quinone derivative by loss of H^+^ from the *para*-hydroxyl group. This is made more favorable by the creating of an extended conjugated π system which spans a large portion of the molecule. This same conjugated p system is responsible for the color used to detect the product via UV/Vis spectroscopy.

Experimentally, no reaction is observed for gallic acid with vanillin on a similar timescale, Scheme A5. No reaction is possible at the equivalent carbon atom to (+)-catechin that has an additional –OH group in gallic acid, but there are two available carbon atoms where an EAS reaction could potentially take place. The rate of reaction at that position is slowed by the presence of a s and p electron withdrawing group (–COOH).

***Scheme A4.*** *EAS rate-determining step between vanillin and gallic acid*

**REFERENCES**

1. “Spectrophotometric Quantitation of Polyphenols Leveraging Vanillin Chemistry.” A Manual of Experiments for Analytical Chemistry. Department of Chemistry at UW- Madison, Spring 2024.

***APPENDIX B****: A Case Study on Calibration Curves – Do Different Regions Have Different Trends?*

*(This document was developed in collaboration with Emily Reasoner, a current graduate student in the Ge & Jin Labs in UW-Madison Chemistry Department.)*

In the lab manual^1^, you are prompted to determine or use a (singular) linear range of the assay. Interestingly enough, Dr. Emily Reasoner (a previous analytical TA and graduate student in our chemistry program) found a scenario where the same molecule can have multiple linear ranges! See her email message below she shared with her students:

As I am going through grading your labs, I am seeing a common "trend" in your reported data. I would like to point this out to you using my own data from a side project of mine.

When you are developing a quantitative method, you need to test a range of concentrations for your calibration curve. The more data points the better. If you are extensive enough (and with some luck), you may be able to see one or more regions with a linear trend. It is common to have different regions with different linear trends!

Please see the example I am attaching. I am working with a collaborator to quantify PFAS (PFAS is bad! read about it if you are curious). We are using a mass-spectrometer to do so. Here, I have identified a lower-concentration linear region (**Figure 1a**) and a higher-concentration linear region (**Figure 1b**). When applying a trendline to these areas separately, I can obtain and R-square value that is very good (>0.99). If I tried to apply a trendline to the entire data set at once, my R-square value would be much lower, and this trendline would not be truly representative of this method! Hope this helps.

***Figure 1****: Two different calibration curves showing 2 different linear ranges, one at low concentrations (a) and one at higher concentrations (b).*

**REFERENCES**

1. “Spectrophotometric Quantitation of Polyphenols Leveraging Vanillin Chemistry.” A Manual of Experiments for Analytical Chemistry. Department of Chemistry at UW- Madison, Spring 2024.
